# Contemporary intergeneric hybridization and backcrossing among birds-of-paradise

**DOI:** 10.1093/evlett/qrae023

**Published:** 2024-06-08

**Authors:** Filip Thörn, André E R Soares, Ingo A Müller, Martin Päckert, Sylke Frahnert, Hein van Grouw, Pepijn Kamminga, Valentina Peona, Alexander Suh, Mozes P K Blom, Martin Irestedt

**Affiliations:** Department of Bioinformatics and Genetics, Swedish Museum of Natural History, Stockholm, Sweden; Department of Zoology, Stockholm University, Stockholm, Sweden; Museum für Naturkunde—Leibniz Institute for Evolution and Biodiversity Science, Berlin, Germany; Department of Medical Biochemistry and Microbiology, National Bioinformatics Infrastructure Sweden, Science for Life Laboratory, Uppsala University, Uppsala, Sweden; Department of Bioinformatics and Genetics, Swedish Museum of Natural History, Stockholm, Sweden; Department of Zoology, Stockholm University, Stockholm, Sweden; Museum für Naturkunde—Leibniz Institute for Evolution and Biodiversity Science, Berlin, Germany; Section Ornithology, Senckenberg Natural History Collections, Museum für Tierkunde, Dresden, Germany; Museum für Naturkunde—Leibniz Institute for Evolution and Biodiversity Science, Berlin, Germany; Bird Group, Department of Life Sciences, Natural History Museum, Tring, Herts, United Kingdom; Naturalis Biodiversity Center, Leiden, the Netherlands; Department of Bioinformatics and Genetics, Swedish Museum of Natural History, Stockholm, Sweden; Swiss Ornithological Institute—Vogelwarte, Sempach, Switzerland; Centre for Molecular Biodiversity Research, Leibniz Institute for the Analysis of Biodiversity Change, Bonn, Germany; Department of Organismal Biology—Systematic Biology, Uppsala University, Uppsala, Sweden; Museum für Naturkunde—Leibniz Institute for Evolution and Biodiversity Science, Berlin, Germany; Department of Bioinformatics and Genetics, Swedish Museum of Natural History, Stockholm, Sweden

**Keywords:** hybridization, sexual selection, lekking, introgression, birds

## Abstract

Despite large differences in morphology, behavior and lek-mating strategies the birds-of-paradise are known to hybridize occasionally, even across different genera. Many of these bird-of-paradise hybrids were originally described as distinct species based on large morphological differences when compared to recognized species. Nowadays, these specimens are generally recognized as hybrids based on morphological assessments. Having fascinated naturalists for centuries, hybrid specimens of birds-of-paradise have been collected and the specimens kept in Natural History Collections. In the present study, we utilize this remarkable resource in a museomics framework and evaluate the genomic composition of most described intergeneric hybrids and some intrageneric hybrids. We show that the majority of investigated specimens are first-generation hybrids and that the parental species, in most cases, are in line with prior morphological assessments. We also identify two specimens that are the result of introgressive hybridization between different genera. Additionally, two specimens exhibit hybrid morphologies but have no identifiable signals of hybridization, which may indicate that minor levels of introgression can have large morphological effects. Our findings provide direct evidence of contemporary introgressive hybridization taking place between genera of birds-of-paradise in nature, despite markedly different morphologies and lek-mating behaviors.

## Teaser

Bird-of-paradise hybrids from different genera can be fertile! Using museomics, we investigate 37 bird-of-paradise hybrids from Natural History Collections. We show that the majority of these specimens are first-generation hybrids and that the parental species, in most cases, are in line with prior morphological assessments. However, two specimens are identified as intergeneric backcrosses. These are the first direct evidence that gene flow between divergent bird-of-paradise species is possible, despite large differences in morphology and lekking behavior.

## Introduction

Hybrids have historically been regarded as evolutionary dead-ends in animal taxa as species boundaries were believed to be rigid (reviewed in Dowling & Secor, 1997). However, several studies have shown that introgressive hybridization has evolutionary implications and occurs at a broad evolutionary timescale across the Tree of Life (Eberlein et al., 2019; Fu et al., 2022; Sankararaman et al., 2014; Svardal et al., 2019; Zhang et al., 2016). Today, species boundaries are regarded as semipermeable, where certain genomic regions are more susceptible to introgression than others (Harrison & Larson, 2014). Yet, the development of genomic incompatibilities that make the hybrid offspring sterile or nonviable will increase with the evolutionary distance between hybridizing species and will result in postzygotic barriers to introgression (Dasmahapatra et al., 2012; Huerta-Sánchez et al., 2014). In addition, the prevalence of hybridization can also be reduced by the emergence of prezygotic isolating mechanisms such as strong sexual selection and assortative mating. Surprisingly, hybridization between distant species is occasionally observed even in organismal groups with lek-mating behaviors. Lek-mating behavior is arguably one of the most extreme forms of sexual selection. Species in such systems often have markedly different phenotypic decorations and mating behaviors, which should act as prezygotic barriers to gene flow (Coughlan & Matute, 2020; Coyne & Orr, 2004). In such systems, males do not hold high-quality territories or construct nests but instead, aggregate in groups and attempt to attract females through male–male competition and/or elaborate courtship displays to demonstrate their value (Balmford, 1991; Rathore et al., 2023). As such, males do not provide any external resource, i.e., parental care or territory, to attract females.

In birds, hybridizing species that reproduce at leks include capercaillie with black grouse (*Tetrao urogallus* × *Lyrurus tetrix*; Kleven et al., 2020), sage grouse with dusky grouse (*Centrocercus urophasianus* × *Dendragapus obscurus*; Rensel & White, 1988), and some species of the genus *Manacus* (Bennett et al., 2021). Mayr (1963) suggested that the lack of pair formation prior to copulation in species with lekking systems may explain why hybrids are unexpectedly common in systems with these kinds of mating behaviors, but no other biological explanation for this phenomenon was provided. In birds, hybrid males are more likely to be viable compared to female hybrids, according to Haldane’s rule (Haldane, 1922). However, in lekking species, the males are simultaneously under strong sexual selection, and it remains unclear to what extent hybrid characteristics are at a disadvantage in systems with this extreme form of sexual selection. Thus, even though hybridization may take place between lekking species, it is unclear if and to what extent contemporary hybridization can lead to introgression.

The birds-of-paradise (*Paradisaeidae*) are a well-known example of sexual selection where female choice has resulted in the development of extreme plumages and complex courtship behaviors in males among species reproducing in leks (Ligon et al., 2018). Despite apparent strong prezygotic reproductive barriers, birds-of-paradise hybridize occasionally even across genera, and more than 20 hybrid combinations have been described based on morphology alone (see Frith & Beehler, 1998 and Supplementary Material for a more in-depth history of bird-of-paradise hybrids). Using whole-genome resequence data for almost all birds-of-paradise species, Blom et al. (in press) recently demonstrated that interspecific hybridization has been a recurring theme throughout the evolutionary history of this group. Moreover, ancestral hybridization has repeatedly led to introgression despite extreme forms of sexual selection. They present 10 morphological hybrids and determine them to be F_1_ hybrids. Whether interspecific hybridization between current species of birds-of-paradise can still lead to introgression or whether contemporary hybrids are sterile remains undetermined.

This study aims to further investigate hybrids from natural contemporary bird-of-paradise populations in an attempt to find direct evidence of backcrossing hybrids. We use museomics to sequence 27 contemporary hybrid specimens, which, together with the ten contemporary hybrids from Blom et al. (in press) cover the most known intergeneric bird-of-paradise hybrid combinations. We confirm the hybrid identity of 24 of them and provide the first genomic evidence of hybrid fertility by reporting two specimens that are the outcome of backcrossing between genera. We discuss the occurrence of hybrids in this lek-mating system in relation to pre- and postzygotic barriers.

## Methods

### Sampling and presequencing processes

This study utilized bird-of-paradise specimens morphologically identified as hybrids hosted in Natural History Collections. The sampling included at least one specimen from all described intergeneric hybrids (Frith & Beehler, 1998; Stresemann, 1930), except the Mysterious Bird of Bobairo [a supposed hybrid between *Epimachus fastuosus* and *Lophorina superba* (Fuller, 1997)], which could not be located. We also included some intrageneric hybrids of species combinations where the parental species were morphologically clearly divergent. Since we detected a putative intergeneric hybrid in a population genomic study of *Astrapia* and *Paradigalla* (Thörn et al., under review), this sample was added to the study.

In total, toepads from 37 Bird-of-Paradise hybrids were obtained from study skins in Natural History Collections (Table 1), of which 10 were included in Blom et al. (in press). All pre-PCR processes were carried out in separate laboratory facilities, which are exclusively used for historical DNA and follow the cleaning regimes and standards in the field of museomics. DNA extractions were carried out using the QIAmp DNA Micro Kit (Qiagen), and libraries were built using a modified Illumina sequencing library preparation protocol by Meyer and Kircher (2010). For detailed laboratory procedures, extraction protocols and library preparation methods, see Irestedt et al. (2022). Four independent, dual-indexed libraries were prepared for each sample and 12 individuals (or 48 indexed libraries) were pooled on a S4 flow-cell (2 × 100 bp) on the Illumina Nova-seq 6000 platform, which were sequenced at the Science for Life Laboratory in Stockholm. All new raw reads, as well as the raw reads for the reference data (Blom et al., in press) have been deposited at the European Nucleotide Archive (PRJEB64275, PRJEB74433, and PRJEB73831).

**Table 1.** Summary of all hybrids investigated and the result of our assessment.

Sample ID	DoC	Vernacular name	Morphological assessment	Sex	mtDNA identity	PCAngsd identity	NGSadmix identity	Morphology = genetic	Hybrid level
DipxPar90521	14.28	Ruys’ Bird-of-Paradise	*Cicinnurus magnificus* × *Paradisaea minor*	Male	*Cicinnurus magnificus*	*Cicinnurus* × *Paradisaea*	*Cicinnurus* × *Paradisaea*	Yes	F1
Eelliot1881511696	21.63	Elliot’s Bird-of-Paradise	*Epimachus fastosus* × *Astrapia nigra*	Male	*Paradigalla carunculata*	*Epimachus* × *Paradigalla*	*Epimachus* × *Paradigalla*	No	F1
EpiAst118	24.64	False-lobed Astrapia	*Paradigalla carunculata* × *Epimachus fastosus*	Male	*Astrapia nigra*	*Astrapia* × *Paradigalla*	*Astrapia* × *Paradigalla*	No	F1
EpiAst119	8.34	Astrapian Sicklebill	*Astrapia nigra* × *Epimachus fastosus*	Male	*Astrapia nigra*	*Astrapia* × *Epimachus*	*Astrapia* × *Epimachus*	Yes	F1
LopAst113	16.48	Rotschild's Lobe-billed Bird-of-Paradise	*Paradigalla carunculata* × *Lophorina superba*	Male	*Lophorina superba*	*Lophorina/Ptiloris* × *Paradigalla*	*Lophorina/Ptiloris* × *Paradigalla*	Yes	F1
LopCic115	27.2	Wilhelmina’s Bird-of-Paradise	*Lophorina superba* × *Cicinnurus magnificus*	Male	*Cicinnurus magnificus*	*Lophorina*/*Ptiloris* × *Cicinnurus*	*Lophorina*/*Ptiloris* × *Cicinnurus*	Yes	F1
LopPti116	20.54	Duivenbode’s Riflebird	*Lophorina superba* × *Ptiloris (magnificus) intercedens*	Male	*Ptiloris magnificus*	*Lophorina superba* × *Ptiloris magnifcus*	*Lophorina superba* × *Ptiloris magnifcus*	Yes	NA
Lptilor19084101	49.21	Sharpe’s Lobe-billed Riflebird	*Paradigalla carunculata* × *Parotia sefilata*	Male	*Parotia sefilata*	*Paradigalla* × *Parotia*	*Paradigalla* × *Parotia*	Yes	F1
LsuxPca331103	14.76	Stresemann’s Bird-of-Paradise	*Parotia carolae* × *Lophorina superba*	Female	*Parotia carolae*	*Parotia* × *Diphyllodes*	*Parotia* × *Diphyllodes*	No	F1
LsuxPsef1910227	16.31	Six-wired Bird-of-Paradise	*Parotia sefilata* × *Lophorina superba*	Male	*Parotia sefilata*	*Lophorina/Ptiloris* × *Parotia*	*Lophorina/Ptiloris* × *Parotia*	Yes	F1
Lwillhe14280	11.07	Wilhelmina’s Bird-of-Paradise	*Lophorina superba* × *Cicinnurus magnificus*	Male	*Cicinnurus magnificus*	*Lophorina/Ptiloris* × *Cicinnurus*	*Lophorina/Ptiloris* × *Cicinnurus*	Yes	F1
Parblood037682	15.34	Captain Blood’s Bird-of-Paradise	*Paradisaea raggiana* × *Paradisornis rudolphi*	Male	*Paradisornis rudolphi*	*Paradisornis* × *Paradisaea*	*Paradornis* × *Paradisaea*	Yes	NA
ParLop117	18.45	Six-wired Bird-of-Paradise	*Parotia sefilata* × *Lophorina superba*	Male	*Parotia sefilata*	*Lophorina/Ptiloris* × *Parotia*	*Lophorina/Ptiloris* × *Parotia*	Yes	F1
ParxPtil90583	8.73	Bensbach´s Bird-of-Paradise	*Ptiloris magnificus* × *Paradisaea minor*	Male	*Paradisaea minor*	*Lophorina/Ptiloris* × *Paradisaea*	*Lophorina/Ptiloris* × *Paradisaea*	Yes	F1
PbreX075320	9.25	Short-tailed Paradigalla	*Paradigalla brevicauda*	Male	*Paradigalla brevicauda*	*Astrapia* × *Paradigalla*	*Paradigalla*	No	BC
PmaxLsu19391171	12.13	Duivenbode’s Riflebird	*Lophorina superba* × *Ptiloris (magnificus) intercedens*	Male	*Ptiloris magnificus*	*Lophorina superba* × *Ptiloris magnifcus*	*Lophorina superba* × *Ptiloris magnifcus*	Yes	NA
PmaxSme19841218	11.59	Mantou’s Riflebird	*Seleucidis melanoleucus* × *Ptiloris magnificus*	Male	*Seleucidis melanoleucus*	*Lophorina/Ptiloris* × *Seleucides*	*Lophorina/Ptiloris* × *Seleucides*	Yes	F1
SelLop109	26.06	Mantou’s Riflebird	*Seleucidis melanoleucus* × *Ptiloris magnificus*	Male	*Seleucidis melanoleucus*	*Lophorina/Ptiloris* × *Seleucides*	*Lophorina/Ptiloris* × *Seleucides*	Yes	F1
SelLop112	21.83	Mantou’s Riflebird	*Seleucidis melanoleucus* × *Ptiloris magnificus*	Male	*Seleucidis melanoleucus*	*Lophorina/Ptiloris* × *Seleucides*	*Lophorina/Ptiloris* × *Seleucides*	Yes	F1
SelPar100	19.49	Wonderful Bird-of-Paradise	*Seleucidis melanoleucus* × *Paradisaea minor*	Male	*Seleucidis melanoleucus*	*Paradisaea* × *Seleucides*	*Paradisaea* × *Seleucides*	Yes	F1
SelPar101	14.27	Wonderful Bird-of-Paradise	*Seleucidis melanoleucus* × *Paradisaea minor*	Male	*Seleucidis melanoleucus*	*Paradisaea* × *Seleucides*	*Paradisaea* × *Seleucides*	Yes	F1
SelxLoph679110	28.23	Mantou’s Riflebird	*Seleucidis melanoleucus* × *Ptiloris magnificus*	Male	*Seleucidis melanoleucus*	*Lophorina/Ptiloris* × *Seleucides*	*Lophorina/Ptiloris* × *Seleucides*	Yes	F1
SelxLoph679111	9.46	Mantou’s Riflebird	*Seleucidis melanoleucus* × *Ptiloris magnificus*	Male	*Seleucidis melanoleucus*	*Lophorina/Ptiloris* × *Seleucides*	*Lophorina/Ptiloris* × *Seleucides*	Yes	F1
SelxPti90520	18.64	Mantou’s Riflebird	*Seleucidis melanoleucus* × *Ptiloris magnificus*	Male	*Seleucidis melanoleucus*	*Lophorina/Ptiloris* × *Seleucides*	*Lophorina/Ptiloris* × *Seleucides*	Yes	F1
SelxPtiZMUC	5.95	Mantou’s Riflebird	*Seleucidis melanoleucus* × *Ptiloris magnificus*	Male	*Seleucidis melanoleucus*	*Lophorina/Ptiloris* × *Seleucides*	*Lophorina/Ptiloris* × *Seleucides*	Yes	F1
SmexLma271158	8.6	Mantou’s Riflebird	*Seleucidis melanoleucus* × *Ptiloris magnificus*	Male	*Seleucidis melanoleucus*	*Lophorina/Ptiloris* × *Seleucides*	*Lophorina/Ptiloris* × *Seleucides*	Yes	F1
SmexLma3297	6.21	Mantou’s Riflebird	*Seleucidis melanoleucus* × *Ptiloris magnificus*	Male	*Seleucidis melanoleucus*	*Lophorina/Ptiloris* × *Seleucides*	*Lophorina/Ptiloris* × *Seleucides*	Yes	F1
CmaxReg22131	26.48	Lyre-tailed king Bird-of-Paradise	*Diphyllodes magnificus* × *Cicinnurus regius*	Male	*Diphyllodes magnificus*	*Diphyllodes* × *Cicinnurus*	*Diphyllodes* × *Cicinnurus*	Yes	BC
CmaxReg303	16.45	King-of-Holland’s Bird-of-Paradise	*Diphyllodes magnificus* × *Cicinnurus regius*	Male	*Diphyllodes magnificus*	*Diphyllodes* × *Cicinnurus*	*Diphyllodes* × *Cicinnurus*	Yes	F1
DipxCic783	18.44	King-of-Holland’s Bird-of-Paradise	*Diphyllodes magnificus* × *Cicinnurus regius*	Female	*Cicinnurus regius*	*Cicinnurus regius*	*Cicinnurus regius*	No	Not a Hybrid
DipxCic782	9.2	King-of-Holland’s Bird-of-Paradise	*Diphyllodes magnificus* × *Cicinnurus regius*	Male	*Diphyllodes magnificus*	*Diphyllodes magnificus*	*Diphyllodes magnificus*	No	Not a Hybrid
PgulxRag20001135	20.91	Maria’s Bird-of-Paradise	*Paradisaea guilielmi* × *Paradisaea raggiana*	Male	*Paradisaea guilielmi*	*Paradisaea guilielmi* × *Paradisaea*	*Paradisaea guilielmi* × *Paradisaea*	Yes	NA
PguxRag31049	21.12	Maria’s Bird-of-Paradise	*Paradisaea guilielmi* × *Paradisaea raggiana*	Male	*Paradisaea guilielmi*	*Paradisaea guilielmi* × *Paradisaea*	*Paradisaea guilielmi* × *Paradisaea*	Yes	NA
PmfinxGuil19128663	24.53	Duivenbode’s Bird-of-Paradise	*Paradisaea minor* × *Paradisaea guilielmi*	Male	*Paradisaea guilielmi*	*Paradisaea guilielmi* × *Paradisaea*	*Paradisaea guilielmi* × *Paradisaea*	Yes	NA
Schodde40100	0.04	Schodde's Bird-of-Paradise	*Parotia lawesii* × *Paradisaea rudolphi margaritae*	Female	NA	NA	NA	NA	NA
CmaxReg18835241	9.7	King-of-Holland's Bird-of-Paradise	*Diphyllodes magnificus* × *Cicinnurus regius*	?	NA	NA	NA	NA	NA
Eellot9933	0.14	Elliot's Bird-of-Paradise	*Epimachus fastosus* × *Astrapia nigra*	Male	NA	NA	NA	NA	NA

*Note.* Table containing: sample ID, depth of coverage (DoC), vernacular name, morphological assessment, sex, mtDNA identity, PCAngsd identity, NGSadmix identity, morphological assessment = genetic assessment, and hybrid level. An extended table including voucher, original description, and source of morphological assessment can be seen in Supplementary Table S1.

To identify the parental combinations and genomic composition for each hybrid, we used a data set that includes genomes for all bird-of-paradise species (Supplementary Table S2; Blom et al., in press; Prost et al., 2019) as reference material. The nomenclature in the present study follows the taxonomy of International Ornithological Congress World Bird List (IOC: Gill et al., 2023).

### Read cleaning

We have used the nf-polish pipeline built specifically for postsequencing processing of historical DNA (https://github.com/MozesBlom/nf-polish). In short, raw reads were used to produce a Fastqc report (v. 0.11.8; Andrews, 2010) of each library to assess the success of the sequencing as well as an initial assessment of the sequence quality. The pipeline then removes PCR duplicates (v. 1.3.3; HTStream/hts_SuperDeduper; https://s4hts.github.io/HTStream/), trims adapters (v. 0.39; Trimmomatic; Bolger et al., 2014), merges overlapping forward and reverse reads (v. 0.9.11; PEAR; Zhang et al., 2014), conducts quality trimming (v. 0.39; Trimmomatic; Bolger et al., 2014), and removal of low complexity reads (custom script; removes reads consisting of more than 50% of a single nucleotide).

### Mitochondrial genome assembly

The mitochondrial scaffolds were assembled using a custom Nextflow pipeline (https://github.com/FilipThorn/nf_mito-mania). In short, a random subset of 5 million cleaned reads was used to assemble a de-novo mitochondrial genome backbone using MitoBIM (v. 1.9.1; Hahn et al., 2013). MitoBIM requires a scaffold to use as a starting seed for the de novo assembly algorithm and we used the mitochondrial reference of *Lycocorax obiensis*. As *L. obiensis* is a phylogenetic outgroup to the “core” birds-of-paradise (the focal group of this study), it minimizes the introduction of potential reference biases. All cleaned reads were then mapped against the mitochondrial genome backbone using the BWA-mem algorithm following the same process as for the nuclear DNA. Variants were called from the mapped mitochondrial genome (freebayes v. 1.3.1; Garrison & Marth, 2012) and added to the mitochondrial genome backbone to obtain consensus sequences (bcftools v. 1.12; Danecek et al., 2021). Positions with a read depth below 20× and above three times the average depth were masked.

### Mitochondrial phylogeny

The mitochondrial genome of all hybrids, as well as the mitochondrial genome of at least one representative from each bird-of-paradise species, were aligned with MAFFT (MAFFT v. 7.407; Katoh et al., 2002). The “pure” bird-of-paradise mitochondrial genomes were obtained from (Blom et al., in press). MAFFT was run using the parameter settings—globalpairs and—maxiterate of 1,000. The resulting alignment was then used to construct a mitochondrial phylogeny using RAxML-NG with the GTR-G model and 100 bootstrap iterations (RAxML-NG v. 1.1.0; Stamatakis, 2014). The phylogeny was rooted using all individuals from the genera *Lycocorax*, *Phonygammus*, and *Manucodia*.

### Nuclear genome mapping

The cleaned reads were mapped against the *L. (pyrrhopterus) obiensis* reference genome (Peona et al., 2021). The mapping was carried out using the BWA-mem algorithm (v. 0.7.17; Li & Durbin, 2009). Read groups were added with Picard (v. 2.10.13; https://broadinstitute.github.io/picard/), and the SAM files were converted to BAMs before merging the unpaired and paired-end reads (Picard v. 2.10.13; https://broadinstitute.github.io/picard/) of each individual. Mapped genomes were indexed using samtools index (samtools v. 1.2; Danecek et al., 2021), and QualiMap (v. 2.2.1; Okonechnikov et al., 2016) was used to assess the success of the genome reconstruction. The extent of postmortem damage was visually assessed with mapdamage2 (Jónsson et al., 2013).

### Genotype likelihoods, PCA, and admixture

Genotype likelihoods were called in ANGSD (v. 0.933; Korneliussen et al., 2014) for all autosomes using the BAM files (parameter sets in Supplementary Material). The dataset was linkage pruned to every 50th SNP and was used for principal component analyses (PCAngsd v. 0.982; Meisner & Albrechtsen, 2018) and admixture analyses (NGSadmix v. 0.933; Skotte et al., 2013). The pipeline was run for multiple subsets of our dataset, each subset consisting of one hybrid and its morphologically assigned parental genera. The pipeline was repeated with different parental genera for samples deviating from the predicted 50% admixture content for first-generational hybrids. The genotype likelihood calling, linkage pruning, PCAngsd and NGSadmix calculations were implemented with https://github.com/FilipThorn/nf-GL_popstructure.

### Variant calling, Ancestry Informative Markers (AIMs), and hybrid indices

Using the results from the PCA and admixture analyses and mitochondrial identity, we obtained putative parental genera for each hybrid. However, PCA and admixture, in general, lack the resolution to distinguish between F_1_, F_2_/F_3_-hybrids, and backcrossing hybrids (Fitzpatrick, 2012). Therefore, we calculated AIMs to verify F_1_-hybrids and scan for signs of recent introgression. AIMs are loci that exhibit large allele frequency differences between divergent populations (Shriver et al., 2003), which at the phylogenetic level are manifested as substitutions between species or genera (Blom et al., in press). In addition to the AIMs, we estimated interspecific heterozygosity and hybrid indices to identify potential backcrosses with a triangle plot. To extract AIMs and estimate hybrid indices based on allele proportions, we called variants with freebayes (v. 1.3.1; Garrison & Marth, 2012) using *L. obiensis* as a reference genome. The variants were called using the core birds-of-paradise as a population prior to using reads with a minimum mapping quality of 10. The resulting VCF files were filtered (parameters settings in Supplementary Material) using vcftools (v. *0.1.16;*Danecek et al., 2011), allelic primitives were split using vcfallelicprimitives (vcflib v. 2017-04-04; Garrison et al., 2022). Only bi-allelic sites were kept, and indel variation was removed.

AIMs were extracted for sites that were fixed between the parental genera, i.e., *F*_ST_ = 1, using the filtered VCF with the Weir and Cockerham method (Danecek et al., 2011). *F*_ST_ was then calculated between each candidate parental genera and the hybrid individual, respectively. Parental alleles present at each AIM site in the hybrid were extracted based on the *F*_ST_ value for the hybrid and each of the parental genera. We then estimated hybrid indices based on the ratio of parental alleles present in each hybrid (sum of counts of parent1 alleles from homozygote and heterozygous AIMs divided by the total number of alleles) and plotted it against interspecific heterozygosity (counts heterozygous AIMs divided by the total number of AIMs) (Valencia-Montoya et al., 2020). Sex chromosomes were excluded from these calculations. These plots are used to identify first-generation hybrids and potential backcrosses. The hybrids’ AIMs identities were plotted along chromosomes; bins of 100 consecutive AIMs positions were assigned a parental identity as either being homozygote for parent one, homozygote for parent two, or heterozygote. A 75% majority of identity to one of these groups was required to assign the bin identity; otherwise, the bin was labeled “mix” to display uncertainty. Since at least two individuals per group are required to obtain fixed sites, the AIMs analyses were only applicable to a subset of the hybrids. Hybrids between *Lophorina* and *Ptiloris* had to be excluded as only one sample from *Lophorina* was present in the dataset. Intrageneric hybrids within *Paradisaea*, of which one parental species was *Paradisaea guilielmi* had to be excluded for the same reason. This also included one hybrid where one parent was *Paradisornis rudolphi.* Hybrids where one parent was *Seleucides melanoleucus* were grouped together with the genera *Drepanornis* as their phylogenetic relationship allowed them to be grouped together (Blom et al., in press). As such, 28 hybrid combinations fulfilled the phylogenetic requirements for further analyses with AIMs.

### hPSMC

To estimate the initial end of gene flow between the parental genera, i.e., population divergence time, we used F_1_-hybrid PSMC (hPSMC; Cahill et al., 2016). hPSMC calculates PSMC on an artificially created F_1_-hybrid, as the TMRCA between the haplotypes in the artificial F_1_-hybrid constitutes the population divergence time. A pseudo-haplotype was created for each parental genus for all autosomal chromosomes using samtools and bcftools (v. 1.2; Danecek et al., 2021) on the *L. obiensis* reference genome. Bases were kept if the depth was between 8 and 50×, base quality was above 15, and mapping quality was above 15. The haplotypes were then converted to a single psmcfa-file using a bin size of 100 bases and a minimum coverage of 50% per bin. PSMC (Li & Durbin, 2011), was implemented on the artificial F_1_-hybrid with the parameter set -N25, -t15, -r6, -p “4 + 25 * 2 + 4 + 6.” The PSMC curves were plotted using a mutation rate of 1.4e-09 (Nadachowska-Brzyska et al., 2016) and a generation time of 8 years (BirdLife International, 2023). The robustness of the divergence time estimate was tested with 100 bootstrap replicates. PSMC curves for the parental species were calculated and added to each hybrid, respectively. All plotting was done with R using the ggplot2 library (R Core Team, 2022; Wickham, 2016).

## Results

### Sequencing results

Since DNA from museum samples is fragmented and generally occurs in low concentrations, there is an increased risk of contamination compared to fresh samples. Additionally, footpad samples from relatively large birds, such as several species of birds-of-paradise, often have lower quantities of endogenous DNA than smaller birds (Irestedt et al., 2022). To control for potential cross-contamination, the presence of heterozygous sites in the mitochondrial genomes was assessed. The majority of the samples had close to zero heterozygous sites and were thus included in downstream analyses. However, three samples had an excess of heterozygous sites in their mitochondrial genome and were excluded from all downstream analyses (see Supplementary Material for excluded samples). The samples included in the study have a nuclear genomic coverage between 35.55% and 97.18% (mean: 84.85%, median: 91.26%), an average read depth between 4 and 49× (mean: 17×, median: 16×) and an average read length between 66 and 133 bp (mean: 111 bp, median: 113 bp).

### Mitochondrial phylogeny

**Figure 1.**
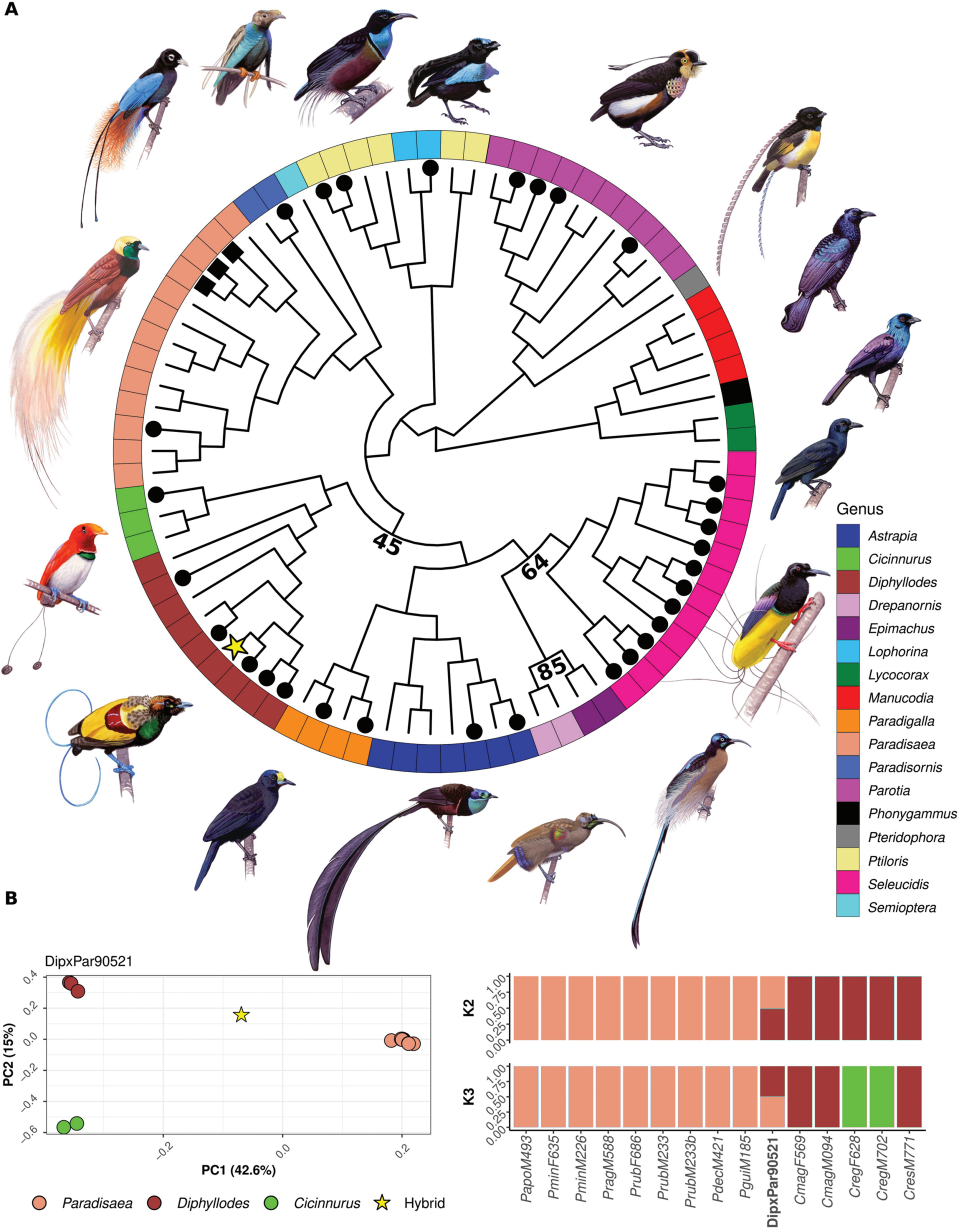
(A) Mitochondrial phylogeny of *Paradisaeidae* and morphologically identified hybrids. Intergeneric hybrids are marked with a terminal black orb, intrageneric hybrids are marked with a terminal black square, and genera are colored and given in an outer circle. DipxPar90521 is marked with a yellow star. The phylogeny was rooted using all species from *Lycocorax*, *Phonygammus*, and *Manucodia*. All samples with index “mt” in column “Subset indices” in Supplementary Table S2 were used to generate the phylogenetic tree. All nodes between genera received full bootstrap support with three exceptions that are marked with bootstrap values. Bird illustrations by ©Szabolcs Kokay and used with permission. (B) Example of PCA (left) and NGSadmix results (right) for one of the hybrids (DipxPar90521) included in the study. Samples marked with index 7 in column “Subset indices” in Supplementary Table S2 were used in combination with DipxPar90521 for these analyses. Both the PCA and NGSadmix (*K* = 2–3) confirm *Diphyllodes* and *Paradisaea* as the parental genera for the hybrid known as Ruys’ Bird-of-Paradise “*Neoparadisea ruysi*” (DipxPar90521), parental genera inferred through PCAngsd and NGSadmix. DipxPar90521 is marked with a yellow star in the PCA plot.

Mitochondrial DNA is maternally inherited in birds; hence, the identity of the maternal species can be obtained from the position of the hybrid specimens within the mitochondrial phylogeny (Figure 1A, Table 1). All bifurcations between genera received full bootstrap support with three exceptions displayed in Figure 1A. The mitochondrial placement of *Lophorina* falls within *Ptiloris*, as *Ptiloris victoriae*/*Ptiloris paradiseus* form a sister clade to *Ptiloris magnificus*/*L. superba*. The maternal identity based on the mitochondrial placement of all hybrids is presented in Table 1.

### Parental genera confirmations

The hybrids’ parental genera were confirmed with PCA and admixture analysis for subsets of each hybrid and morphologically assigned parental species. The subsets were chosen based on the morphological assessment of each hybrid, as well as their mitochondrial identity. As such, 34 subsets consisting of one hybrid and the individuals from its two putative parental genera (or the putative parental species for intrageneric hybrids), were plotted as illustrated in Figure 1B. The results for all the 34 hybrids are seen in Supplementary Figures S1–S34. The morphological assessment did not seem to match the genetic assessment for six hybrids, which were reinvestigated with different parental genera combinations (Supplementary Figures S64–S66). The genetic parental assessment based on PCA and admixture is presented in Table 1.

### Ancestry informative markers

**Figure 2.**
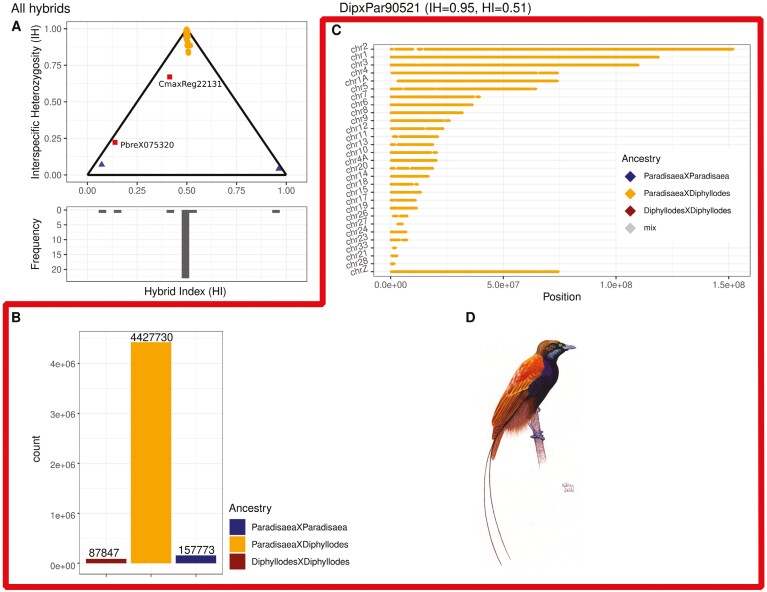
(A) Triangle plot of all hybrids included in this study. Proportion of parental alleles (hybrid index) is presented on the *x*-axis and the proportion of heterozygous AIMs (interspecific heterozygosity) is displayed on the *y*-axis. Frequency distribution of points in the plotting area is presented underneath. Red boxes indicate putative backcrosses, and blue triangles indicate potentially misidentified hybrids. (B) Counts of homozygous and heterozygous AIMs on autosomes in DipxPar90521 indicating its F_1_-hybrid status. Samples marked with index 7 in column “Subset indices” in Supplementary Table S2 were used to produce this plot. (C) AIMs in bins of 100 positions along autosomes and Z-chromosome in DipxPar90521. The high proportion of heterozygous AIMs indicates its F_1_-hybrid status. Samples marked with index 7 in column “Subset indices” in Supplementary Table S2 were used to produce this plot. (D) Illustration based on the hybrid known as Ruys’ Bird-of-Paradise “*Neoparadisea ruysi*” (DipxPar90521) painted by ©Szabolcs Kokay and used with permission.

Admixture proportions by themselves are a coarse estimate of hybridization. Consequently, an F_1_-hybrid can exhibit the same admixture proportion as a F_2_-hybrid (Fitzpatrick, 2012). However, F_1_- and F_2_-hybrids will differ in their proportion of heterozygous sites that are fixed between their parental species. F_2_-hybrids will have a lower proportion of these heterozygous sites. Thus, the proportion of heterozygous AIMs can be used in combination with a hybrid index (HI) to identify F_1_, F_2_/F_3_-hybrids and backcrosses (Bouchemousse et al., 2016; Valencia-Montoya et al., 2020). In Figure 2A, each filled circle represents an intergeneric hybrid. Data points at the top of the triangle have a high proportion of interspecific heterozygous (AIMs) and a balanced HI, which is expected of F_1_-hybrids (most of the hybrids investigated). Data points with an intermediate proportion of interspecific heterozygosity and a balanced HI indicate F_2_- and F_3_-hybrids (none of the hybrids investigated exhibited this pattern). A low proportion of interspecific heterozygosity and an extremely unbalanced HI (where basically only one of the parental species AIMs are detected) suggests that an individual has been misidentified as a hybrid or that the individual has a hybrid proportion that is extremely low. An intermediate proportion of interspecific heterozygosity and an unbalanced HI indicate a potential backcross (two backcrosses). The putative hybrids that were not identified as F_1_-hybrids had prior been morphologically assessed to be hybrids between *Diphyllodes magnificus* and *Cicinnurus regius* (CmaxReg22131: Supplementary Figure S2; DipxCic783: Supplementary Figure S3; DipxCic782: Supplementary Figure S4 in Supplementary Material) and a putative cross between *Paradigalla* and *Astrapia* morphologically assessed as a *Paradigalla brevicauda* (PbreX075320: Supplementary Figure S7).

**Figure 3.**
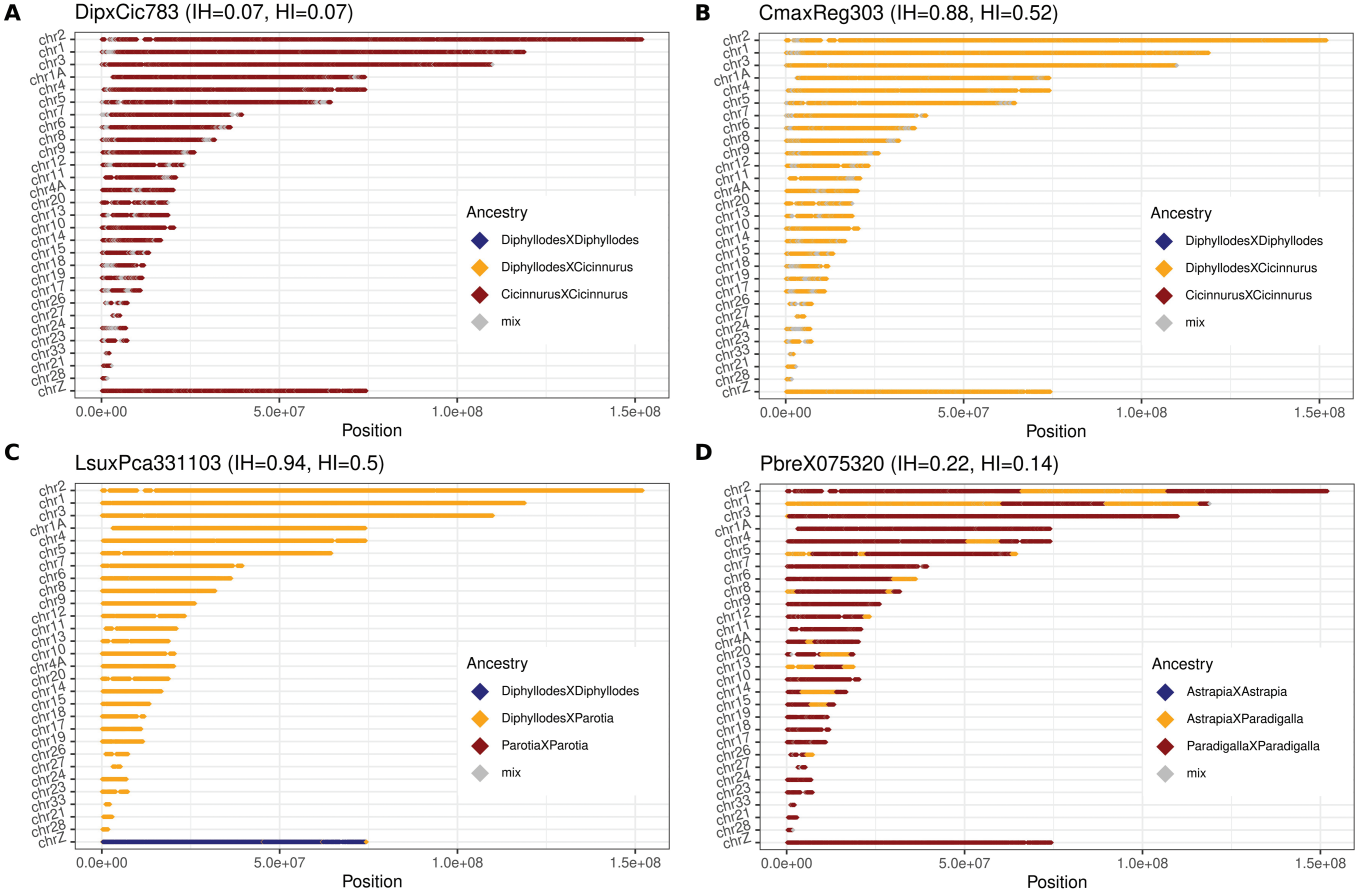
Comparisons of chromosomal patterns of AIMs between a potentially misidentified hybrid, two F_1_-hybrids (♂/♀), and a recent backcrossed hybrid. (A) AIMs in bins of 100 positions along autosomes and Z-chromosome for DipxCic783. The relative frequency of homozygous AIMs with *Cicinnurus* identity indicates no clear signs of contemporary hybridization, and the sample is either a misidentified nonhybrid with an aberrant morphology or has too low levels of hybrid contents to be detected with our current data set. Samples marked with index 3 in column “Subset indices” in Supplementary Table S2 were used to produce this plot. (B) AIMs in bins of 100 positions along autosomes and Z-chromosome for CmaxReg303. The high amount of heterozygous AIMs indicates the sample to be an F_1_-hybrid between *Diphyllodes* and *Cicinnurus*. The sample is a male (♂); as such, the Z-chromosome also display a high amount of heterozygous AIMs. Samples marked with index 3 in column “Subset indices” in Supplementary Table S2 were used to produce this plot. (C) AIMs in bins of 100 positions along autosomes and Z-chromosome for LsuxPca331103. The high amount of heterozygous AIMs along the autosomes indicates the sample to be an F_1_-hybrid between *Diphyllodes* and *Parotia*. The sample is a female (♀). The Z-chromosome displays a high amount of homozygous AIMs inherited from the paternal species. In this case, the paternal genus was *Diphyllodes*. Samples marked with index 11 in column “Subset indices” in Supplementary Table S2 were used to produce this plot. (D) AIMs in bins of 100 positions along autosomes and Z-chromosome for PbreX075320 (♂). The distribution of runs of heterozygous and homozygous AIMs along the autosomes indicates that the sample is the offspring of a hybrid between *Astrapia* and *Paradigalla*, which has backcrossed with *Paradigalla*. The Z-chromosome displays a high amount of homozygous AIMs inherited from the paternal species. In this case, the paternal Z-chromosome identity was *Paradigalla*. Samples marked with index 1 in column “Subset indices” in Supplementary Table S2 were used to produce this plot.

**Figure 4.**
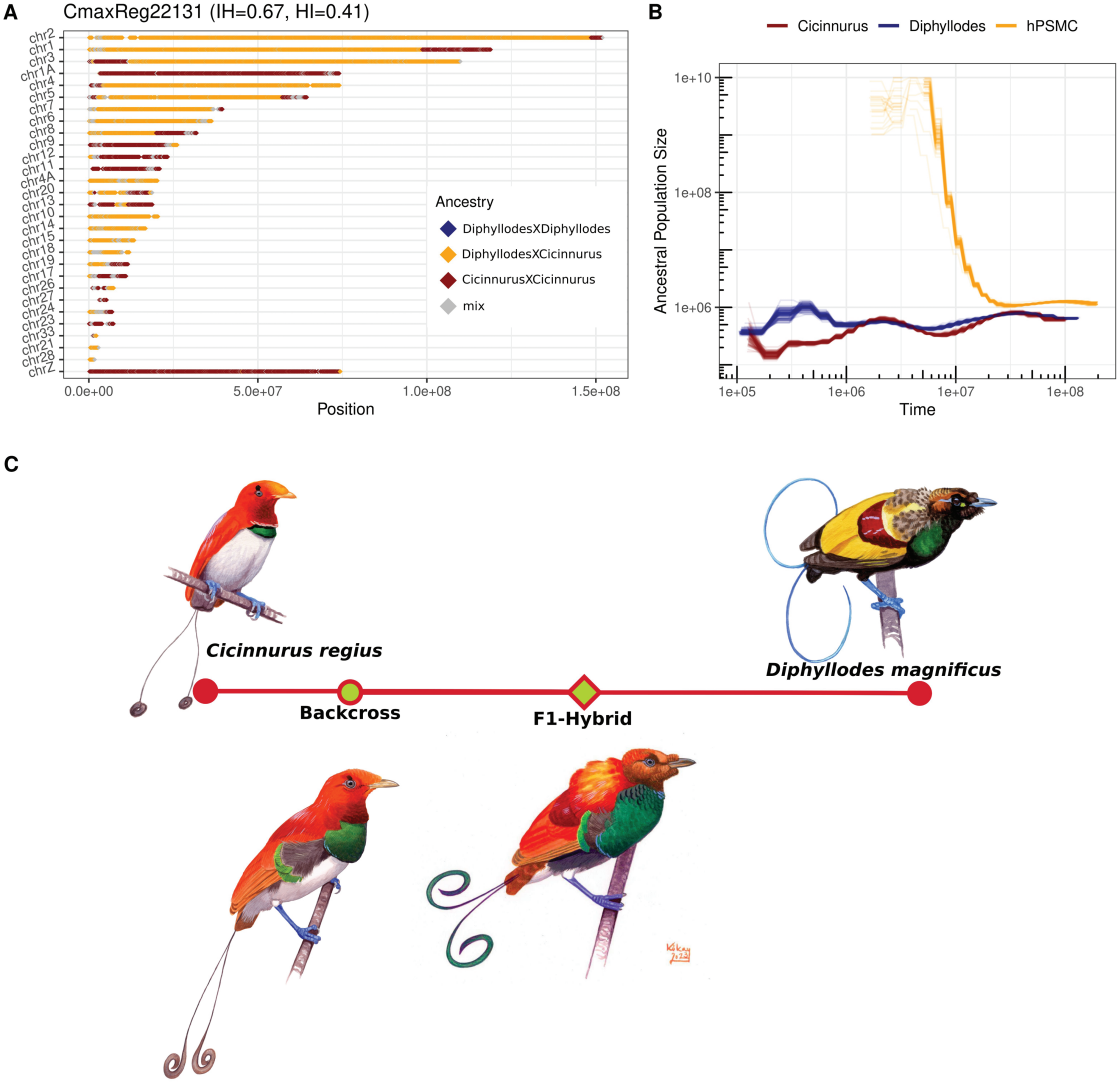
(A) AIMs in bins of 100 positions along autosomes and the Z chromosome in CmaxReg22131 indicating it has experienced recent introgression. Samples marked with index 3 in column “Subset indices” in Supplementary Table S2 were used to produce this plot. (B) hPSMC curve of 100 bootstraps for an artificial F_1_-hybrid between *Diphyllodes* and *Cicinnurus*, indicating that the initial end of gene flow occurred more than 10 Mya (Millions of years ago). PSMC curves of the demography of *Diphyllodes* and *Cicinnurus* were included as well. Plots were generated using a mutation rate of 1.4e-9 and a generation time of 8 years. Samples marked with index 21 in column “Subset indices” in Supplementary Table S2 were used to produce this plot. (C) Illustration of King Bird-of-Paradise (top left), Magnificent Bird-of-Paradise (top right) and these two species’ morphologically diverse hybrid forms, the Lyre-tailed king Bird-of-Paradise (bottom left) and the King of Holland’s Bird-of-Paradise (bottom right). The axis in the middle indicates the hybrid content in the two-hybrid specimens that have been illustrated (CmaxReg22131 and CmaxReg303). As the coloration of bare-parts in museum study skins is partly lost, color of bare-parts in the illustrations of the hybrids has been estimated based on the parental species. Illustrations painted by ©Szabolcs Kokay and used with permission.

Furthermore, we investigated the identity of AIMs along chromosomes in bins of 100 AIMs positions. F_1_-hybrids are expected to have the vast majority of their bins as heterozygous, while misidentified hybrids will consist mainly of homozygous AIMs from one of the parental species. In contrast, recently backcrossed hybrids are expected to have blocks of bins that are heterozygous with otherwise regions of homozygous bins from the recipient population. The length of heterozygous blocks will reflect the recombination pattern's strength of selection and will break down over time (Pool & Nielsen, 2009), long heterozygous blocks indicate fewer generations since the initial hybridization event. The F_1_-hybrids identified in Figure 2A were all consistent with the expected pattern for F_1_-hybrids, with each chromosome being dominated by heterozygous bins (Table 1; Figure 2B, C, Supplementary Figures S35–S58). DipxCic783 (bottom right corner in Figure 2A), which was a female morphologically identified as an intergeneric hybrid between *D. magnificus* and *C. regius* turned out to have a *C. regius* genotype; thus, the specimen was misidentified as a hybrid (Supplementary Figure S60). Likewise, DipxCic782 (bottom left corner in Figure 2A), which was a male morphologically assessed as an intergeneric hybrid between *D. magnificus* and *C. regius* appeared to be a *D. magnificus* misidentified as a morphological hybrid (Supplementary Figure S59). Lastly, both CmaxReg22131 and PbreX075320 have blocks of heterozygous bins within regions of otherwise homozygous bins on some chromosomes (Figure 3D and Supplementary Figure S62: PbreX075320; Figure 4A and Supplementary Figure S61: CmaxReg22131). This mosaic block structure indicated that CmaxReg22131 was the offspring of a hybrid between *D. magnificus* and *C. regius* that had backcrossed with *C. regius*, and PbreX075320 was the offspring of a hybrid between the genera *Paradigalla* and *Astrapia* that had backcrossed with a *Paradigalla s*pecies. The initial hybridization event for CmaxReg22131 occurred fewer generations ago relative to PbreX075320, as indicated by the lengths of the heterozygous blocks (Figure 3D: PbreX075320; Figure 4A: CmaxReg22131). A comparison between the identity of AIMs along chromosomes for one of the misidentified species, two F_1_-hybrids (male and female), and one of the backcrosses can be seen in Figure 3. Since the Z-chromosome is inherited paternally in birds, a female F_1_-hybrids’ Z-chromosome indicates paternal identity (Figure 3A). Out of the 34 putative hybrids investigated, two are the result of recent backcrosses (Figure 3D and Supplementary Figure S62: PbreX075320; Figure 4A and Supplementary Figure S61: CmaxReg22131), two appear to be misidentified nonhybrids (Figure 3A: DipxCic783; Supplementary Figure S59; DipxCic782), and the remaining 24 are F_1_-hybrids (Supplementary Figures S35–S58). However, it should be noted that DipxCic782 is a male sample that morphologically clearly deviates from *D. magnificus*, which suggests a hybrid origin. It is thus possible that this sample has some hybrid contents but that this content is too low to be detected with our current data set.

### Estimated divergence time between backcrossing species

Divergence times between the parental populations were estimated for CmaxReg22131 and PbreX075320 based on hPSMC. The parental genomes were decided based on the PCA plots and mitochondrial identity (CmaxReg22131: Supplementary Figure S2; PbreX075320: Supplementary Figure S7). hPSMC divergence time estimates are both around the magnitude of 10 Mya, seemingly consistent with the demographic dynamics of the parental species (CmaxReg22131: Figure 4B; PbreX075320: Supplementary Figure S63D). As the generation time and mutation rate influence the phase and amplitude of the PSMC curves, the relative divergence times are more informative.

### Congruence between molecular and preassessed morphology affinity

Due to a limited number of individuals for each reference species, the nuclear assessments of parental species generally identified the hybrids at the genus level rather than at the species level. However, given that the mitochondrial genomes identify maternal lineages to species level and the sampling localities for most hybrids are known (only one species per genus normally occurs in the same region), a likely parental species combination can generally be inferred. Overall, our molecular estimations of hybrids’ parental identity match the morphological assessments well, as only six hybrids were assigned to different parentages (Table 1 and Supplementary Material for detailed descriptions).

## Discussion

The birds-of-paradise are one of the most well-known examples of sexual selection in the animal kingdom (Ligon et al., 2018), and their elaborate courtship behaviors and spectacular plumage ornaments have fascinated naturalists for centuries. As these prominent differences should act as prezygotic barriers to gene flow (Coughlan & Matute, 2020; Coyne & Orr, 2004), it is intriguing that birds-of-paradise are known to occasionally produce hybrids (Blom et al., in press; Stresemann, 1930; Fuller, 1979; Frith & Beehler, 1998). This suggests that behavioral and plumage cues, assumed to form prezygotic barriers to gene flow, sometimes fail to assure complete reproductive isolation in this system. Here, we present the first empirical evidence of contemporary intergeneric introgression between morphological and behavioral divergent birds-of-paradise species and demonstrate that these hybrids are able to overcome the barriers imposed by the lek-mating strategy and reproduce.

### The genomic landscape of introgression and weak barriers to gene flow

The length of introgressed tracts across chromosomes is informative on how many generations an individual has backcrossed, as recombination will break down and shorten introgressed tracts over time (Pool & Nielsen, 2009; Racimo et al., 2015). In the two backcross individuals in this study, the longer tracts in CmaxReg22131 (Figure 4A) suggest that fewer backcrossing events have occurred since the initial hybridization compared to PbreX075320 (Figure 3D). This is further supported by the level of interspecific heterozygosity (Figure 2A) and by morphology. The more recent backcross (CmaxReg22131) shows clear morphological signs of hybrid origin (Figure 4C), in contrast to the individual that has backcrossed additional times (PbreX075320) that show no obvious morphological deviations from the species with which it has been backcrossed (*P. brevicauda*).

Our hPSMC divergence estimates suggest that these two examples of introgressive backcrossing have occurred between species that diverged roughly 10 Mya (Figure 3). Although inaccurate estimations of generation time may inflate (or reduce) the divergence time in hPSMC analyses, previous divergence estimates based on mitochondrial data support these results by estimating the divergence time between both these hybridizing species pairs to around 8 Mya (Irestedt et al., 2009). As genomic structural differences can cause genetic conflicts and postzygotic barriers (Kirkpatrick & Barton, 2006), it is intriguing that birds-of-paradise are able to occasionally hybridize across genera that diverged many million years ago and also produce offspring that at least occasionally are fertile. A study comparing satellite DNA between crows (*Corvus*) and birds-of-paradise found that repetitive elements are more conserved across birds-of-paradise (Peona et al., 2023) than between other avian species, which may have implications regarding genetic compatibility as satellite DNA are important components in structural domains of chromosomes (Brajković et al., 2012; Kuhn et al., 2011). According to Haldane’s rule, hybrids from the heterogametic sex, which are females in birds, will be less viable (Haldane, 1922; Schilthuizen et al., 2011). The vast majority of bird-of-paradise individuals who have been identified as hybrids are males (Frith & Beehler, 1998), which is in accordance with Haldane’s rule. However, there is likely a heavy sampling bias as the prominent differences in coloration and ornamentations in males make hybrid males more easily detectable and more desirable to collect. Therefore, neither this study nor the ratio of female and male bird-of-paradise hybrids in museum collections is reliable evidence to determine if female hybrids are more uncommon than male hybrids in birds-of-paradise. However, from the mitochondrial phylogeny (Figure 1A, Table 1), from which the maternal identity of hybrids can be assigned, some interesting patterns emerge. For the 11 hybrids that include *Seleucidis* as one of the parental genera (hybrids between either the *Ptiloris/Lophorina* or *Paradisaea* linages), *Seleucidis* was always the maternal genus (Figure 1A, Table 1). This may indicate that genomic incompatibilities between *Seleucidis* and *Ptiloris*/*Lophorina* or *Paradisaea* make hybrids between male *Seleucidis* and female *Ptiloris*/*Lophorina* or *Paradisaea* nonviable. Genomic incompatibilities are often facilitated by parts of the genome that accumulate changes in allele frequencies faster, i.e., mitochondria or sex chromosomes, which could be drivers of this pattern (Lopez et al., 2021). Alternatively, these patterns could also be driven by prezygotic behavioral mechanisms. In black-capped and mountain chickadees, social dominance is suggested to drive sex biases in interspecific mating (Grava et al., 2012), and *Ptiloris*/*Lophorina* and *Paradisaea* males might likewise be socially dominant to *Seleucidis.* However, the relative frequency of hybrid combinations could be heavily influenced by sampling biases, like more easily identifiable hybrid combinations or breeding ranges being more accessible for collection.

Male hybrids are expected to have higher fitness than female hybrids in birds (Haldane, 1922) and most studies on avian introgression adhere to this pattern (Ottenburghs, 2022), which should dictate introgressive hybridization being facilitated through male hybrids. Many theories also predict female choice as vital in interspecific matings in birds-of-paradise (Christidis & Schodde, 1993; Martin, 2015; Mayr, 1963). For the recent backcross CmaxReg22131, we identify *D. magnificus* as the maternal species based on the mitochondria identity and find the identity of the Z-chromosome to be *C. regius* (Figure 4A). We know this specimen to be a male hybrid, therefore it has two Z-chromosomes, which both have the identity of *C. regius*. This specific combination of Z chromosomes and mitochondria can only occur if the maternal side is a hybrid. In contrast to general predictions based on Haldane’s rule, we thus here provide evidence that a female hybrid has facilitated introgressive hybridization. Therefore, we tentatively suggest that introgressive hybridization between lekking birds-of-paradise might be driven to at least some extent by female hybrids.

### Morphological variation in backcrosses

For hybrids between *D. magnificus* and *C. regius*, two different morphs have been described (Figure 4C): the more common King-of-Holland’s Bird-of-Paradise (also known as King William III’s bird-of-paradise) and the rarer Lyre-tailed king Bird-of-Paradise (Frith & Beehler, 1998). Our specimen of Lyre-tailed king Bird-of-Paradise (CmaxReg22131) is genetically a backcross with *C. regius*, which is corroborated by a plumage that more closely resembles that of *C. regius* than that of *D. magnificus.* The plumage of the King-of-Holland’s Bird-of-Paradise is essentially intermediate between its two parental species, which is in line with that one of the King of Holland’s Bird-of-Paradise specimens (CmaxReg303) is genetically identified as an F_1_-hybrid (Supplementary Figure S42). However, two other individuals morphologically assessed as King of Holland’s Bird-of-Paradise are genetically determined to be either a pure *D. magnificus* (DipxCic782; Supplementary Figures S4 and S63) or a pure *C. regius* (DipxCic783; Supplementary Figures S4 and S63). One of these samples is a female (DipxCic783), which makes it difficult to use morphology to confirm these results (females of these two species are similar). However, the male sample (DipxCic782) is morphologically clearly deviating from *D. magnificus* and morphologically very similar to the F_1_-hybrid King-of-Holland’s Bird-of-Paradise. It is possible that this individual does have low levels of hybrid contents that cannot be confidently detected with our current data set. In contrast, the *Astrapia* × *Paradigalla* backcross shows no obvious morphological deviation from *P. brevicauda*. These results are in line with several recent studies of hybrids showing that the level of hybrid content does not always co-vary with morphological variation (Natola et al., 2023; Toews et al., 2016; Wang et al., 2020). Thus, minor levels of introgression can have large effects on the morphology of an individual and similarly substantial introgression may not result in large morphology deviation.

### Mate choice and hybridization in lekking birds-of-paradise

The large number of viable intergeneric hybrid combinations present in birds-of-paradise raises questions regarding the conditions needed for interspecific mating to occur. In Darwin’s finches (*Geospiza* sp.), species with low relative abundance are more likely to hybridize in accordance with the desperation hypothesis (Grant & Grant, 1997). However, as modeled by Qvarnström et al., (2023), the rate of hybridization increases with one species in low abundance only if mate choice errors are low as well. In contrast, if mate choice errors between species are high the rate of hybridization is higher when the species occur at equal proportions. As the hybridizing bird-of-paradise species tend to have slightly different altitude preferences, it is not unlikely that large differences in relative abundance in overlapping distributions to some degree may explain hybridization. Despite this, it is intriguing that so many viable intergeneric hybrid combinations can be observed in the wild, given their large differences in morphology and courtship behavior during lekking. The lack of pair formation in lekking species (Mayr, 1963), signature of historical reinforcement in birds-of-paradise (Martin, 2015) and choosing novel partners occasionally might be beneficial for females (Christidis & Schodde, 1993) are some proposed explanations for why assortative mating is not perfect in birds-of-paradise. As female plumages often are similar across species (in contrast to male plumages) and as sexual coercion is a well-known phenomenon in birds (McKinney & Evarts, 1998), one may speculate that the frequent occurrence of hybridization in birds-of-paradise to some degree stems from random encounters, where unpaired males mate with interspecific females outside lekking sites.

### Concluding remarks

In this study, we utilized museomics to investigate a remarkable set of morphologically assessed bird-of-paradise hybrids. We provide evidence that birds-of-paradise are capable of hybridizing across deep evolutionary scales and confirm their hybrid identity with genomic methods. Moreover, we present evidence that contemporary intergeneric introgression occurs between species with markedly different morphologies and lekking behaviors. We also find indications that female hybrids are involved in driving intergeneric introgression in this system with an extreme form of sexual selection.

## Supplementary material

Supplementary material is available online at *Evolution Letters*.

qrae023_suppl_Supplementary_Table_S1

qrae023_suppl_Supplementary_Table_S2

qrae023_suppl_Supplementary_Material

## Data Availability

The raw reads for the morphological hybrids and the *Paradigalla* and *Astrapia* hybrid (PbreX075320) have been uploaded to European Nucleotide Archive (ENA) and are accessible through the accession numbers PRJEB74433 and PRJEB73831. Accession numbers for the raw reads of the samples used as reference material are given in Supplementary Table S2. The code used for data processing is available from the github repository (https://github.com/MozesBlom/nf-polish), mitochondrial genome assembly (https://github.com/FilipThorn/nf_mito-mania), genotype likelihood analyses (https://github.com/FilipThorn/nf-GL_popstructure), and Ancestry Informative Markers analysis (https://github.com/FilipThorn/nf-AIMs).
