# Supplementary material for: Contemporary intergeneric hybridization and backcrossing among birds-of-paradise
Source: Evol Lett. 2024 Jun 8;8(5):680–94. doi: 10.1093/evlett/qrae023 (PMC11424083; doi:10.1093/evlett/qrae023)
Supplement: qrae023_suppl_Supplementary_Material [file qrae023_suppl_supplementary_material.pdf]

Supplementary Material

History of birds-of-paradise hybrids

Inspired by an encounter with Lord Rothschild and with the support of Ernst Stresemann, Mayr himself had traveled Papua New Guinea in 1928 in search of some enigmatic bird-of-paradise taxa he had examined in bird collections. All of them had been formally described as a species-level taxon or a subspecific taxon at that time, however, doubts had been raised whether some of those taxa actually represented rare hybrid forms. Mayr's expectation was to discover more of those specimens which Stresemann had named "homeless birds-of-paradise" in the wild (Haffer 1997, p. 234: "heimatlose Paradieser"). Mayr arrived on the Vogelkop Peninsula on 5th April 1928, however after a three months' expedition he returned unsuccessfully without having found a single specimen of those "unica" he had known from bird collections (Gedeon & Kleinstäuber 2004). Mayr considered his journey a failure, however, his unsuccessful attempt encouraged his contemporaries to re-examine the rare specimens documented from bird collections. Later, Stresemann revisited a large series of specimens in the collection of Walter (Lord) Rothschild at Tring Museum, and summarized his diagnosis on a hybrid origin of 18 bird-of-paradise taxa in a review paper: He listed 14 intergeneric combinations for 17 taxon names that were in use at that time plus one intrageneric combination (Stresemann 1930; compare Table 1). This review received great attention among ornithologists and earned the full agreement of Lord Rothschild (1930) in his comment on Stresemann's paper.

References:
Gedeon K, Kleinstäuber G. 2004, Auf den Spuren von Ernst Mayr – Eine Reise des Vereins Sächsischer Ornithologen 2003 nach West-Papua. Mitteilungen des Vereins Sächsischer Ornithologen 9: 279–305.

Haffer J. 1997. Ornithologen-Briefe des 20. Jahrhunderts. Ökologie der Vögel 19: 1–980.

Rothschild Lord W. 1930. Notes on the preceding article of Dr. Stresemann. Novitates Zoologicae. 36:16–17.

Stresemann, E. (1930) Welche Paradiesvogelarten der Literatur sind hybriden Ursprungs? Novitates Zoologicae 36, 6-15.

Library preparations

DNA fragments were cut at uracil sites using USER enzyme followed by blunt-end repair, adapter ligation, and adapter fill in. The length and quality of each library were assessed using agarose gel electrophoresis following a trial PCR at 12 cycles. After the length and concentration assessment of the trial PCR, an index PCR was carried out in four separate independent reactions for each sample, with individual indices. The number of cycles per sample was decided based on the trial PCR and ranged between 8-12 cycles. The number of cycles was kept low to decrease PCR duplicates in the sequencing data. The four separate reactions were used to achieve an even amplification across each sample's genome, as well as facilitating post sequencing processing per library instead of limiting the filtering to individual samples. The PCR products were then pooled together per sample followed by magnetic bead cleaning. The concentrations of the pooled PCR products were measured using Qubit 3.0 broad range and diluted to 10 ng/ul when above. The fragment length distribution and exact concentration were measured with bioanalyser before equimolar

pooling of individual samples.

ANGSD parameter settings

The parameter set used to run ANGSD v.0.933
-uniqueOnly 1
-minMapQ 20
-minQ 20
-doGlf 2
-doMajorMinor 1
-skipTriallelic 1
-doMaf 1
-minMaf 0.05
-SNP_pval 1e-6
-doCounts 1
-minInd (parameter set to the number of individuals in the subset)
-setMinDepthInd 2
-setMinDepth 20

Variant call filtering

The parameter set used to filter VCF using vcftools v.0.1.16 and vcflib v.2017-04-04
–minQ 20
–min-meanDP 2
–max-meanDP 160
–minDP 10
–maxDP 75
–max-missing 0.9
–remove-indels
–min-alleles 2
–max-alleles 2
–maf 0.02

Excluded samples

Three samples had an excess of heterozygous sites in their mitochondrial genome and were excluded from all downstream analyses. The excluded samples were; Schodde’s birds of paradise (Schodde40100; depth: 0.04X, covered bases: 0.65%), a sample of King of Holland’s bird of paradise (CmaxReg18835241; depth: 9.7X, covered bases: 61%) and a sample of Elliot’s bird of paradise (Eellot9933; depth:0.14X, covered bases: 15%). The study included replicates of the King of Holland’s bird of paradise and a sample of Elliot’s bird of paradise. However, Schodde’s bird of paradise is an unreplaceable and unique sample, which has been morphologically identified as a hybrid between *Parotia wahnesi* and *Paradisornis rudolphi*.

Congruence between molecular and pre-assessed morphology affinity

The Elliot's Bird-of-Paradise is morphologically assessed as a hybrid between *Epimachus fastosus* and *Astrapia nigra*, but we find it to be a hybrid between *Epimachus* and *Paradigalla*. Given that the sampling locality is presumably the Vogelkop Peninsula, and the mitochondrial genome is of *Paradigalla carunculata* identity, the Elliot's Bird-of-Paradise is most certainly a hybrid between *Epimachus fastosus* and *Paradigalla carunculata*. The False-lobed Astrapia is morphologically estimated as a hybrid between *Paradigalla carunculata* and *Epimachus fastosus*, but as the mitochondrial genome is of *Astrapia nigra* identity and the sampling locality is the Vogelkop Peninsula it is a hybrid between *Paradigalla carunculata* and *Astrapia nigra*. The Stresemann´s Bird-of-Paradise is morphologically characterised as a hybrid between *Parotia carolae* and *Lophorina superba*. However, the mitochondrial identity is Parotia carolae and the nuclear assessment assigned it as a cross between *Parotia* and *Diphyllodes*. The specimen was collected at Mount Hunstein in the Sepik district, Stresemann´s Bird-of-Paradise is therefore a hybrid between *Parotia carolae* and *Diphyllodes magnificus*.

Supplementary Figures

PCA and admixture analysis

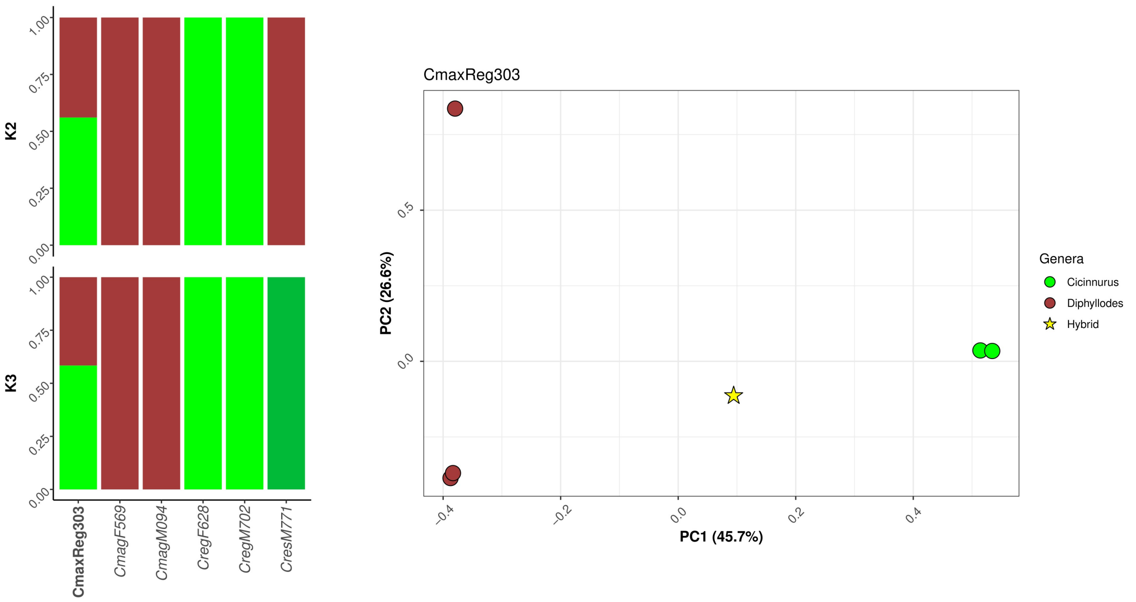

FigureS1. PCA and admixture analysis K=2..3 confirming parental genera implemented through PCAngsd and NGSAdmix. Hybrid is marked with a yellow star in the PCA plot. Samples marked with index 3 in table

S2 were used in combination with the hybrid CmaxReg303 to produce this plot.

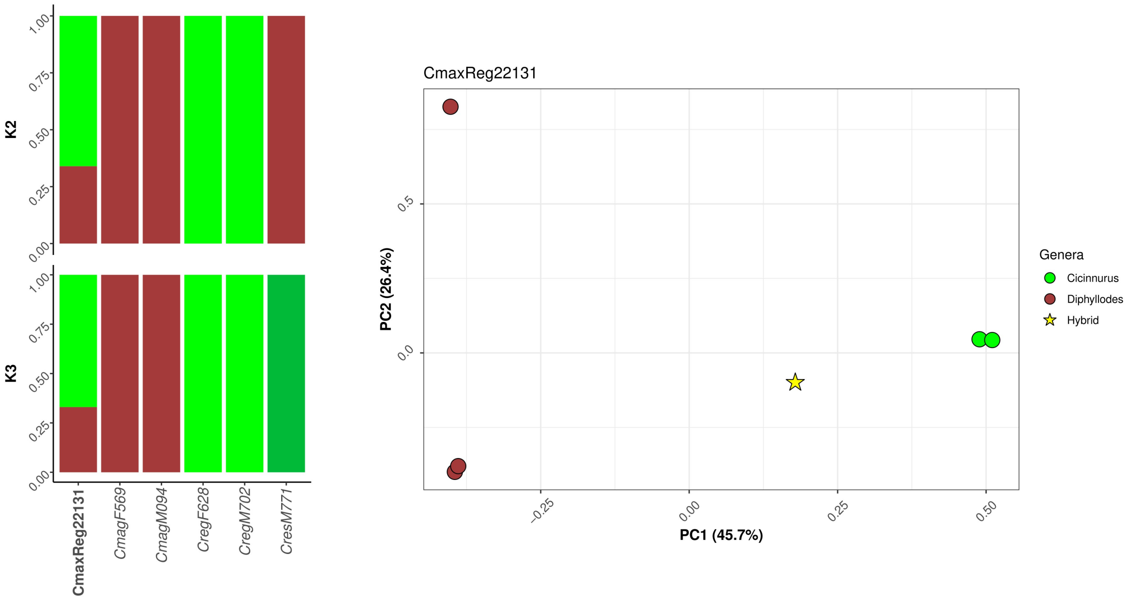

FigureS2. PCA and admixture analysis K=2..3 confirming parental genera implemented through PCAngsd and NGSAdmix. Hybrid is marked with a yellow star in the PCA plot. Samples marked with index 3 in table S2 were used in combination with the hybrid CmaxReg22131 to produce this plot.

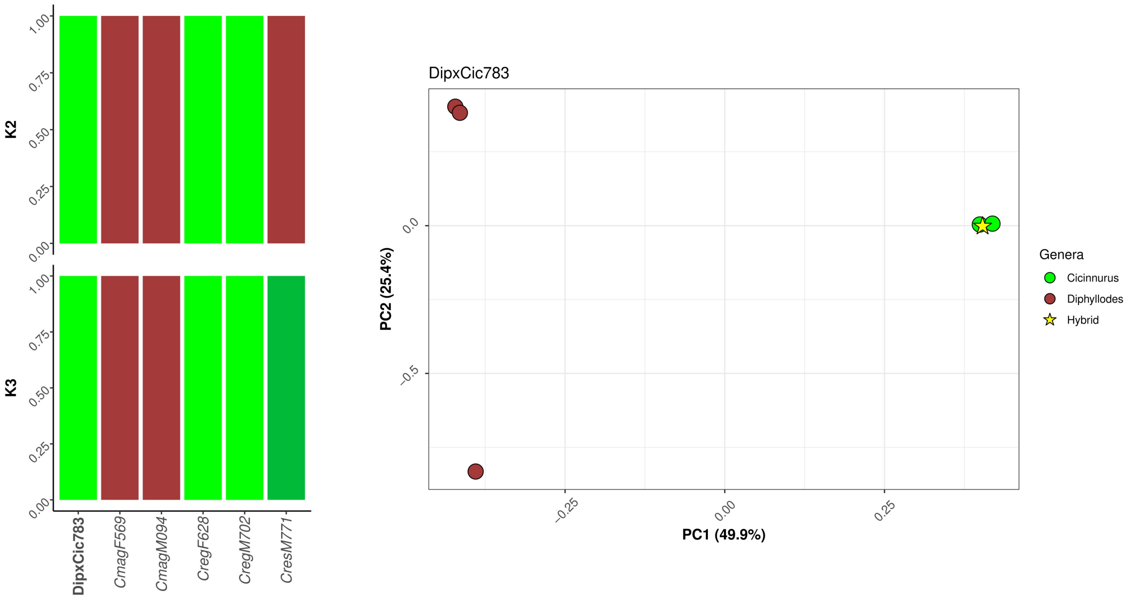

FigureS3. PCA and admixture analysis K=2..3 confirming parental genera implemented through PCAngsd and NGSAdmix. Hybrid is marked with a yellow star in the PCA plot. Samples marked with index 3 in table S2 were used in combination with the hybrid DipxCic783 to produce this plot.

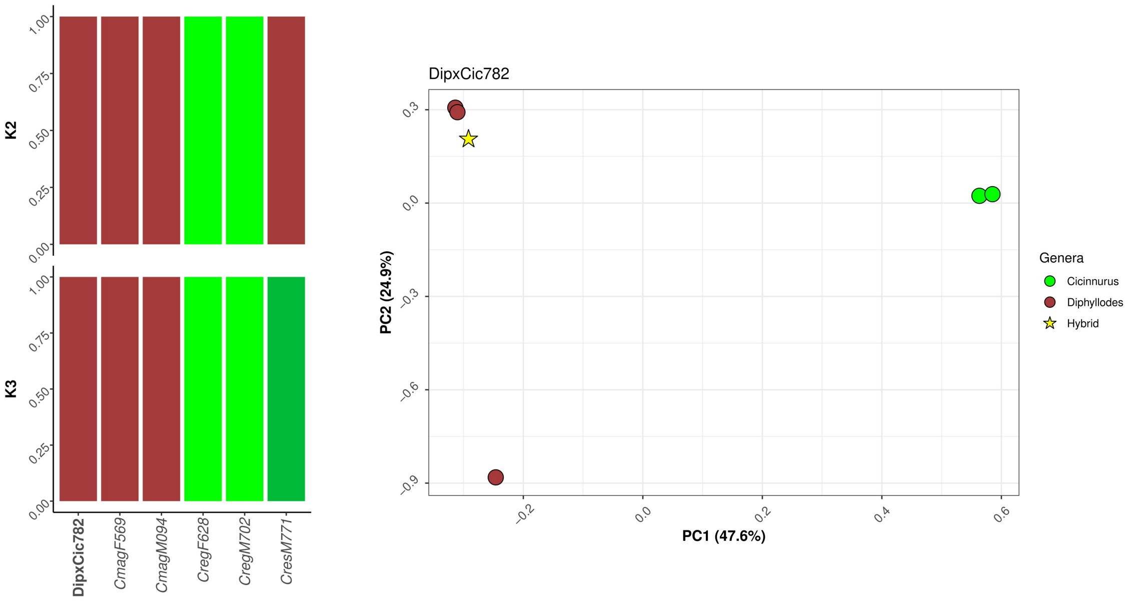

FigureS4. PCA and admixture analysis K=2..3 confirming parental genera implemented through PCAngsd and NGSAdmix. Hybrid is marked with a yellow star in the PCA plot. Samples marked with index 3 in table S2 were used in combination with the hybrid DipxCic782 to produce this plot.

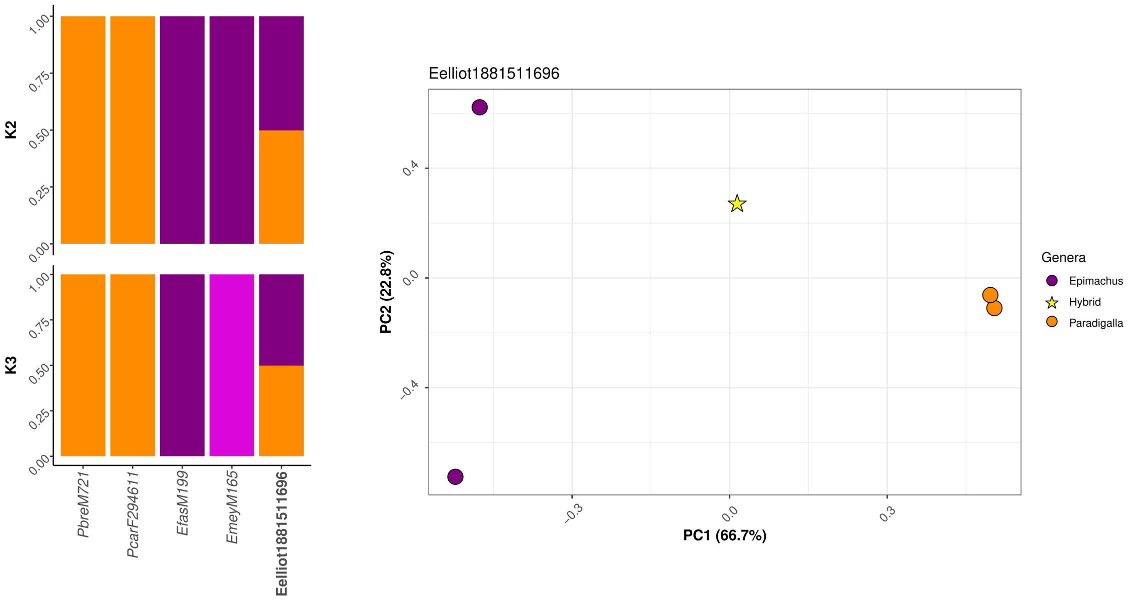

FigureS5. PCA and admixture analysis K=2..3 confirming parental genera implemented through PCAngsd and NGSAdmix. Hybrid is marked with a yellow star in the PCA plot. Samples marked with index 4 in table S2 were used in combination with the hybrid Eelliot1881511696 to produce this plot.

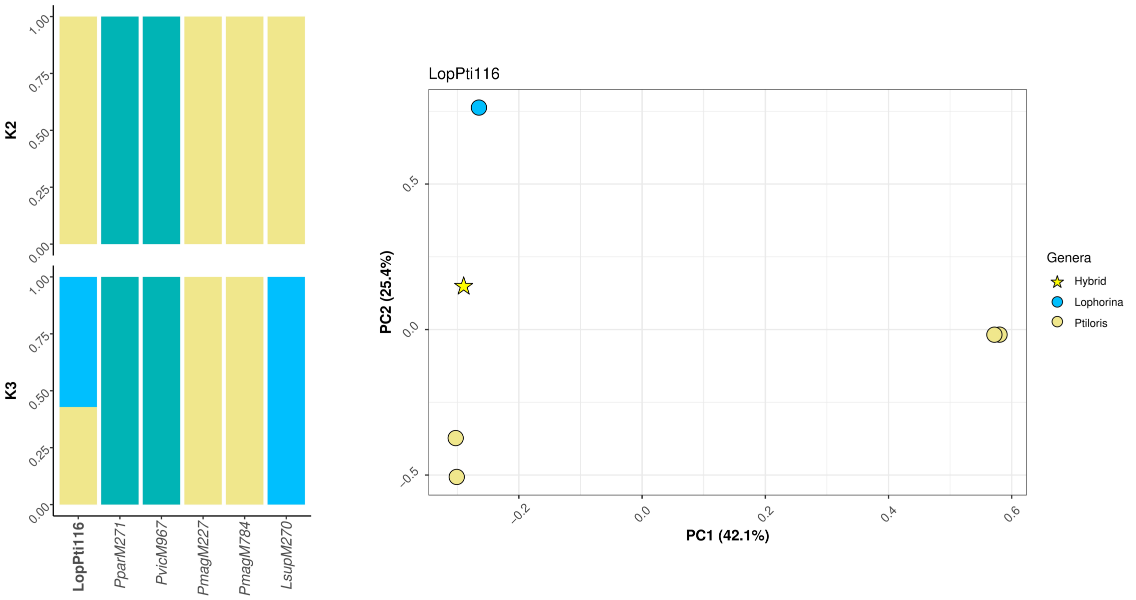

FigureS6. PCA and admixture analysis K=2..3 confirming parental genera implemented through PCAngsd and NGSAdmix. Hybrid is marked with a yellow star in the PCA plot. Samples marked with index 5 in table S2 were used in combination with the hybrid LopPti116 to produce this plot.

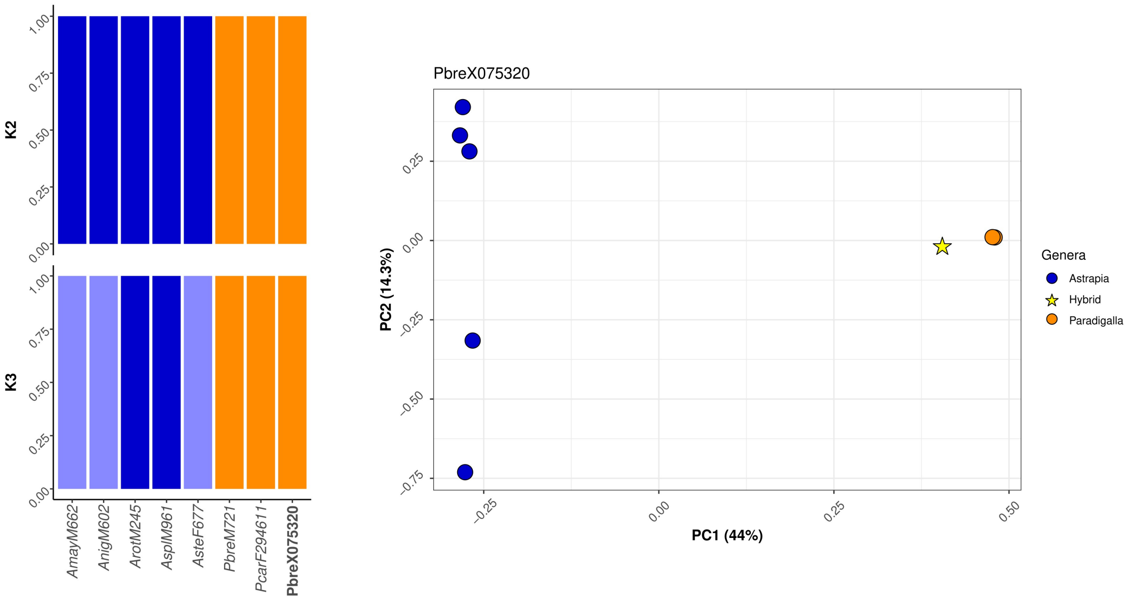

FigureS7. PCA and admixture analysis K=2..3 confirming parental genera implemented through PCAngsd and NGSAdmix. Hybrid is marked with a yellow star in the PCA plot. Samples marked with index 1 in table S2 were used in combination with the hybrid PbreX075320 to produce this plot.

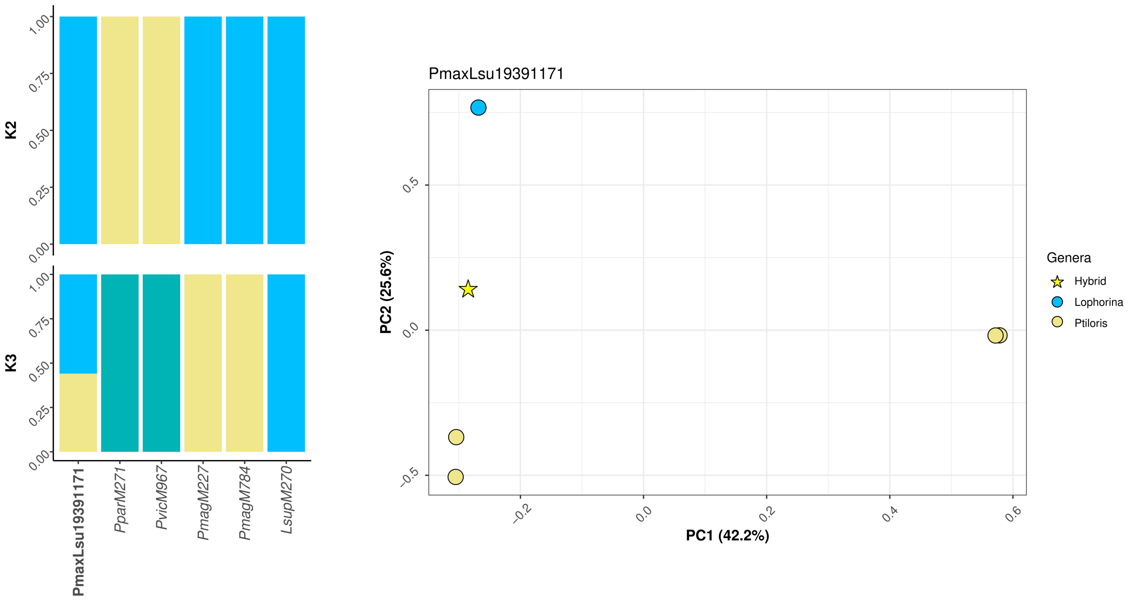

FigureS8. PCA and admixture analysis K=2..3 confirming parental genera implemented through PCAngsd and NGSAdmix. Hybrid is marked with a yellow star in the PCA plot. Samples marked with index 5 in table S2 were used in combination with the hybrid PmaxLsu19391171 to produce this plot.

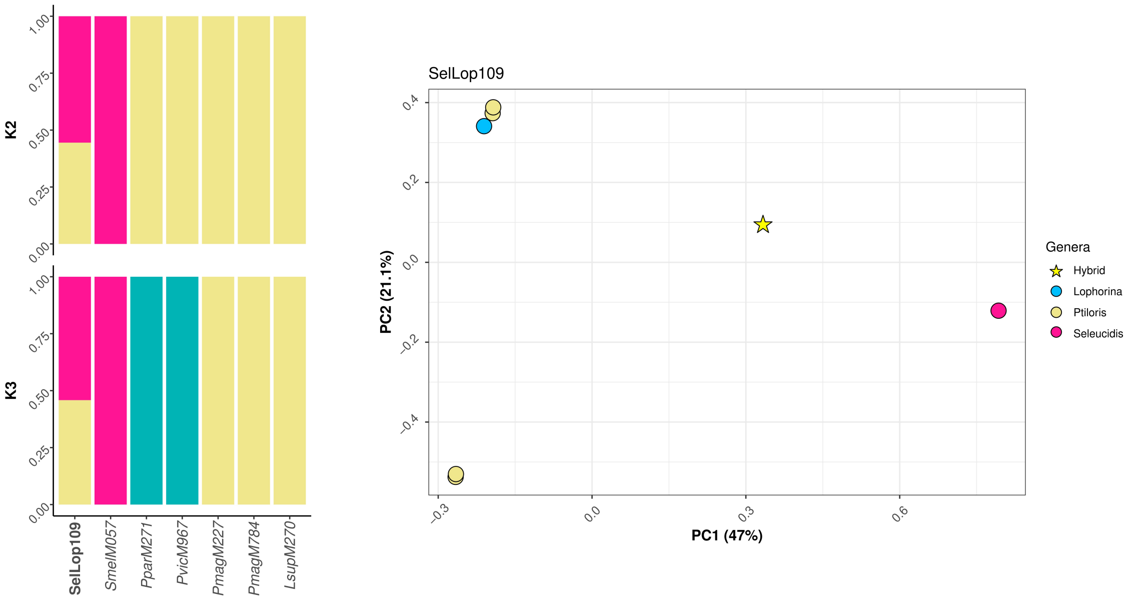

FigureS9. PCA and admixture analysis K=2..3 confirming parental genera implemented through PCAngsd and NGSAdmix. Hybrid is marked with a yellow star in the PCA plot. Samples marked with index 6 in table S2 were used in combination with the hybrid SelLop109 to produce this plot.

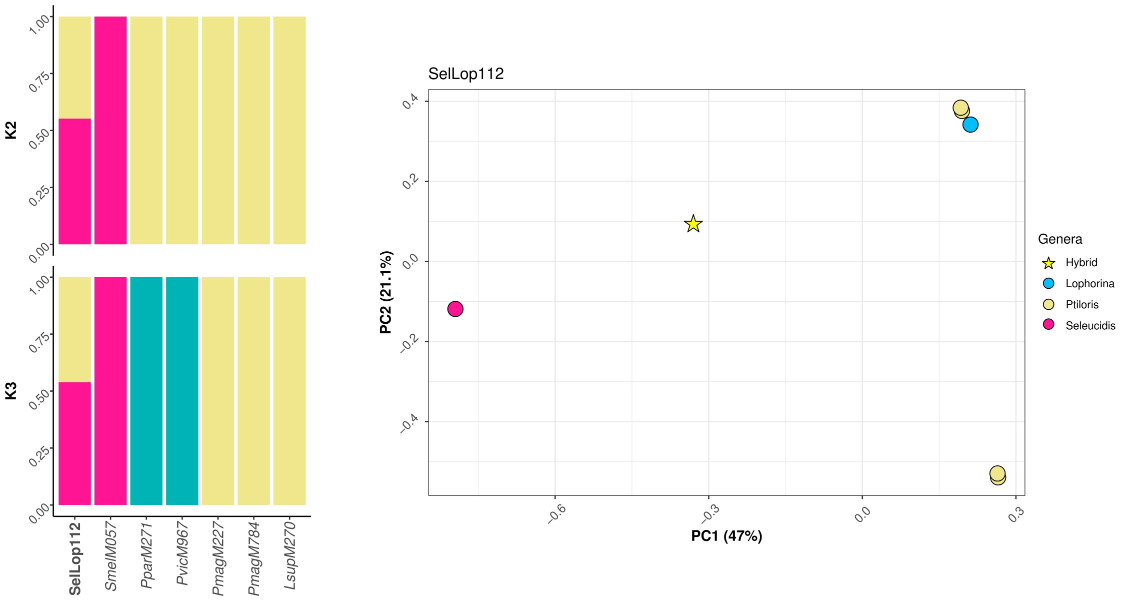

FigureS10. PCA and admixture analysis K=2..3 confirming parental genera implemented through PCAngsd and NGSAdmix. Hybrid is marked with a yellow star in the PCA plot. Samples marked with index 6 in table S2 were used in combination with the hybrid SelLop112 to produce this plot.

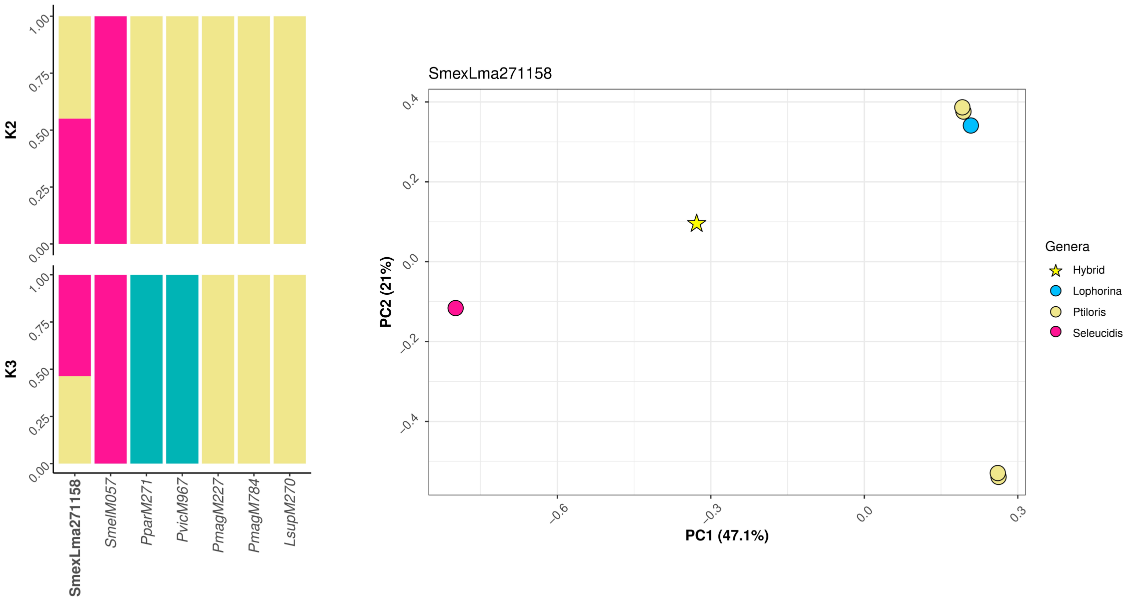

FigureS11. PCA and admixture analysis K=2..3 confirming parental genera implemented through PCAngsd and NGSAdmix. Hybrid is marked with a yellow star in the PCA plot. Samples marked with index 6 in table S2 were used in combination with the hybrid SmexLma271158 to produce this plot.

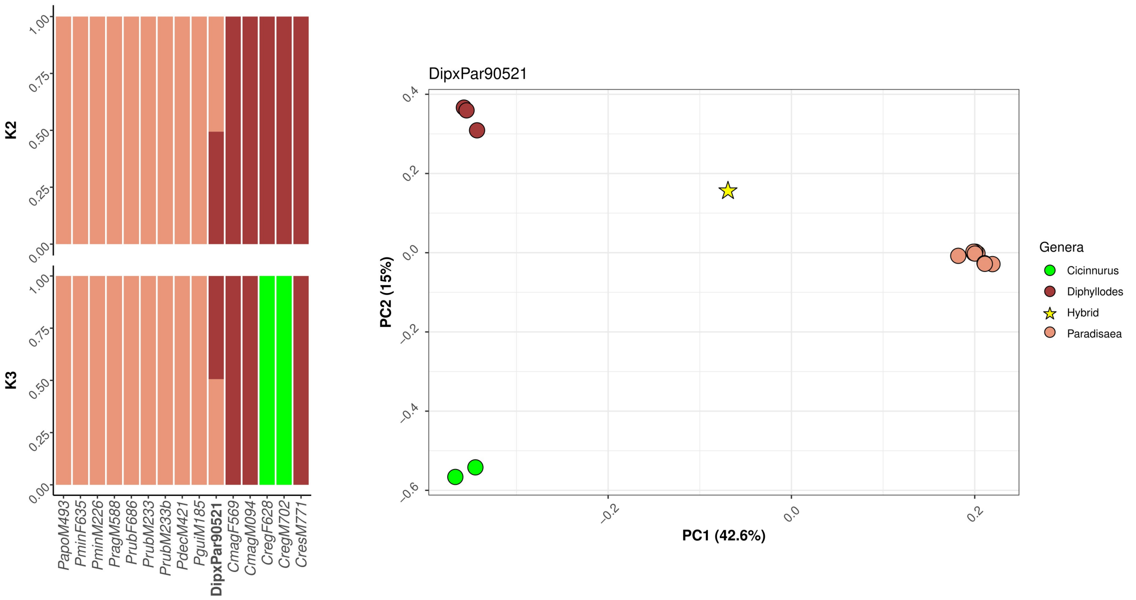

FigureS12. PCA and admixture analysis K=2..3 confirming parental genera implemented through PCAngsd and NGSAdmix. Hybrid is marked with a yellow star in the PCA plot. Samples marked with index 7 in table S2 were used in combination with the hybrid DipxPar90521 to produce this plot.

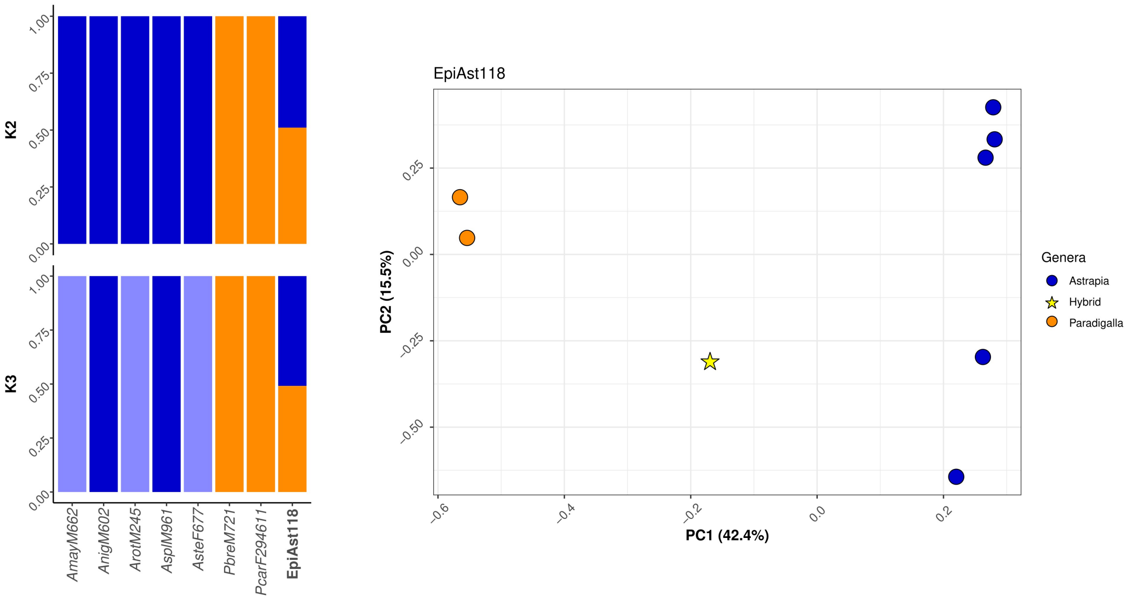

FigureS13. PCA and admixture analysis K=2..3 confirming parental genera implemented through PCAngsd and NGSAdmix. Hybrid is marked with a yellow star in the PCA plot. Samples marked with index 1 in table S2 were used in combination with the hybrid EpiAst118 to produce this plot.

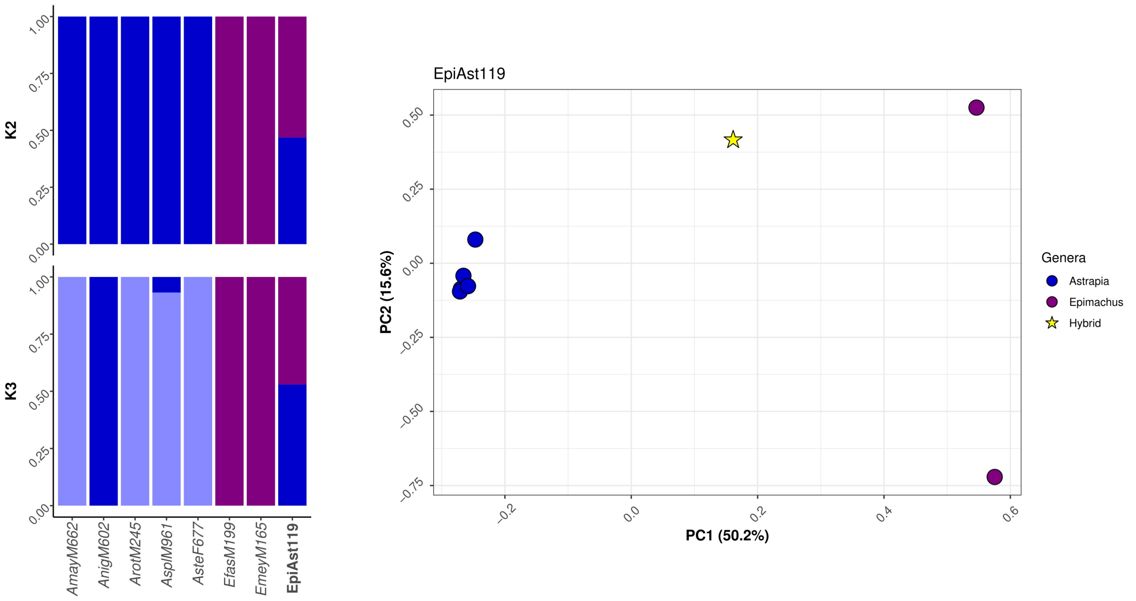

FigureS14. PCA and admixture analysis K=2..3 confirming parental genera implemented through PCAngsd and NGSAdmix. Hybrid is marked with a yellow star in the PCA plot. Samples marked with index 2 in table S2 were used in combination with the hybrid EpiAst119 to produce this plot.

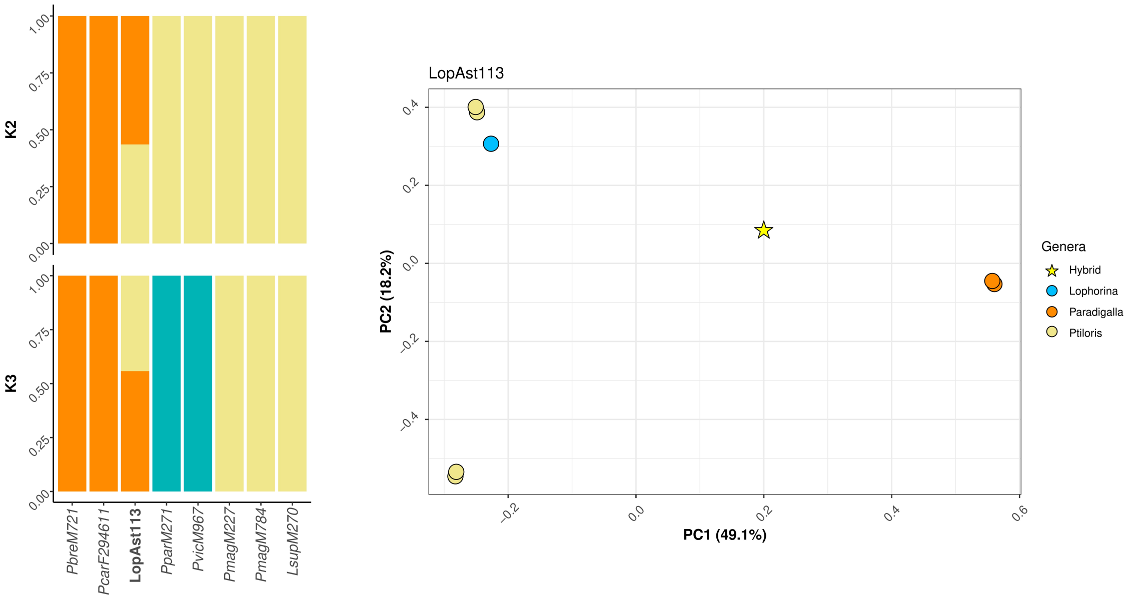

FigureS15. PCA and admixture analysis K=2..3 confirming parental genera implemented through PCAngsd and NGSAdmix. Hybrid is marked with a yellow star in the PCA plot. Samples marked with index 8 in table S2 were used in combination with the hybrid LopAst113 to produce this plot.

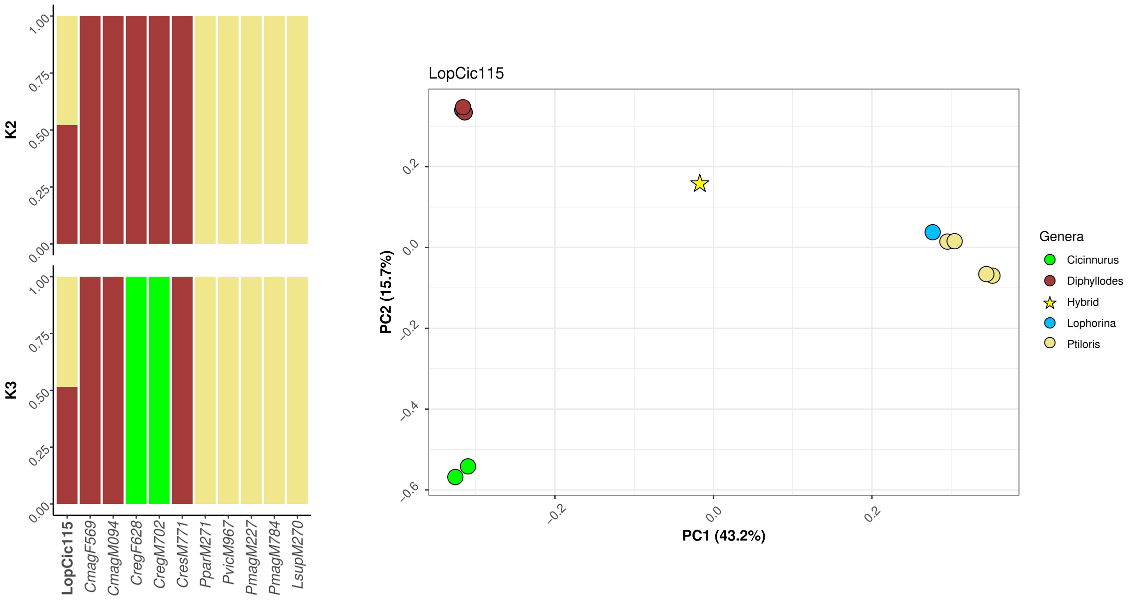

FigureS16. PCA and admixture analysis K=2..3 confirming parental genera implemented through PCAngsd and NGSAdmix. Hybrid is marked with a yellow star in the PCA plot. Samples marked with index 9 in table S2 were used in combination with the hybrid LopCic115 to produce this plot.

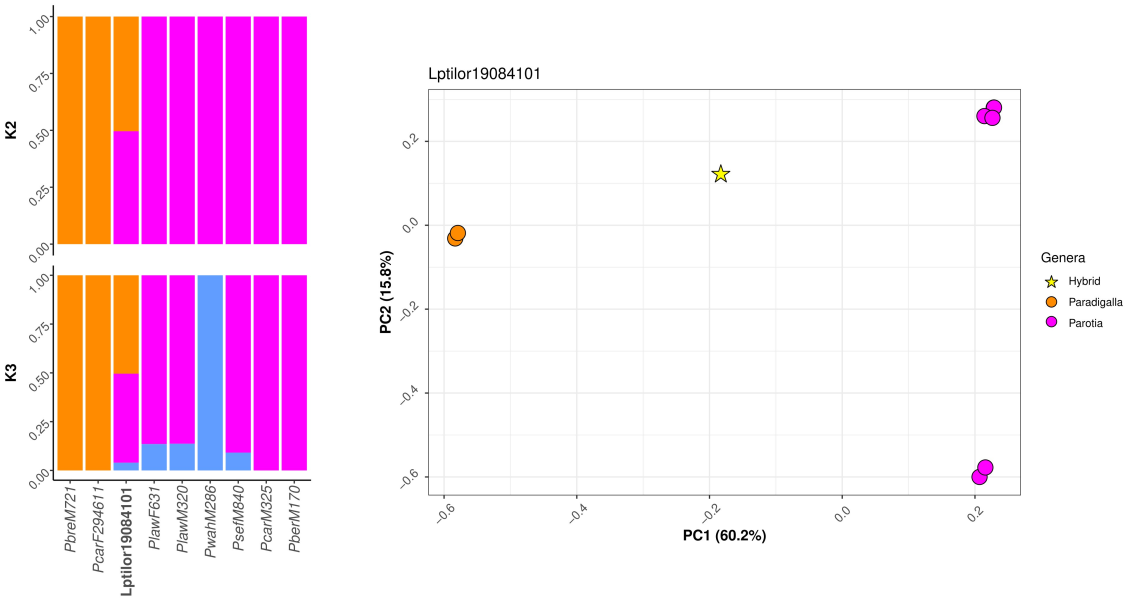

FigureS17. PCA and admixture analysis K=2..3 confirming parental genera implemented through PCAngsd and NGSAdmix. Hybrid is marked with a yellow star in the PCA plot. Samples marked with index 10 in table S2 were used in combination with the hybrid Lptilor19084101 to produce this plot.

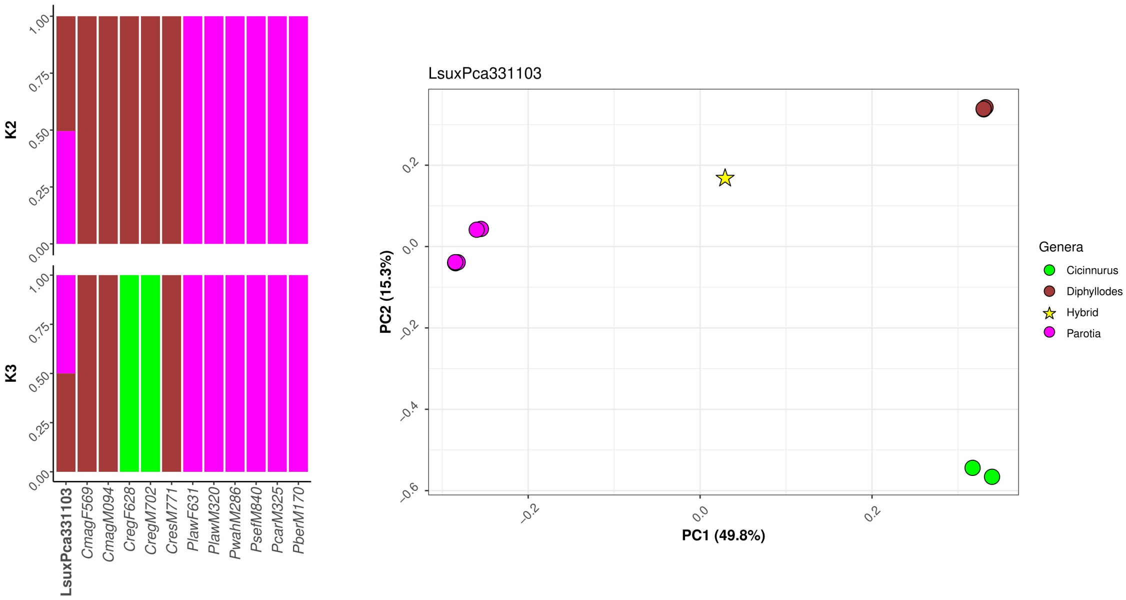

FigureS18. PCA and admixture analysis K=2..3 confirming parental genera implemented through PCAngsd and NGSAdmix. Hybrid is marked with a yellow star in the PCA plot. Samples marked with index 11 in table S2 were used in combination with the hybrid LsuxPca331103 to produce this plot.

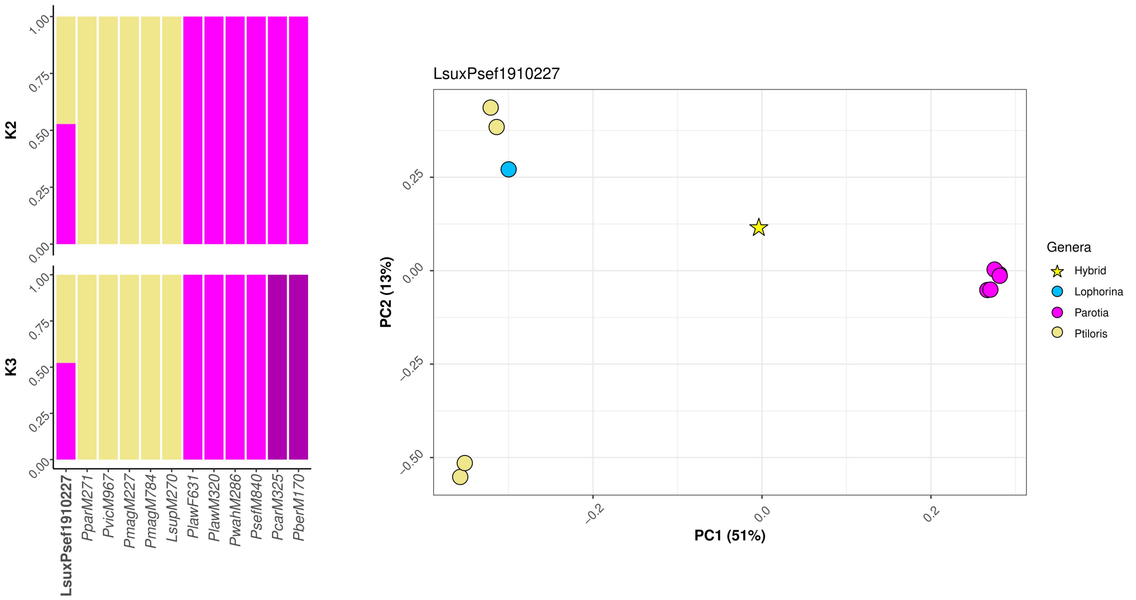

FigureS19. PCA and admixture analysis K=2..3 confirming parental genera implemented through PCAngsd and NGSAdmix. Hybrid is marked with a yellow star in the PCA plot. Samples marked with index 12 in table S2 were used in combination with the hybrid LsuxPsef1910227 to produce this plot.

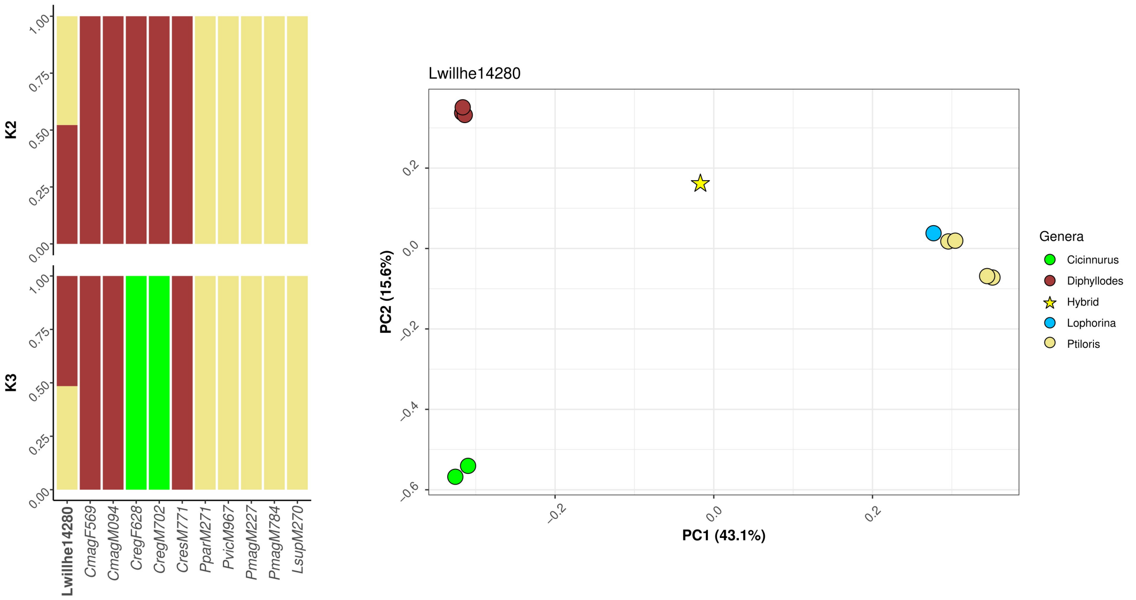

FigureS20. PCA and admixture analysis K=2..3 confirming parental genera implemented through PCAngsd and NGSAdmix. Hybrid is marked with a yellow star in the PCA plot. Samples marked with index 9 in table S2 were used in combination with the hybrid Lwillhe14280 to produce this plot.

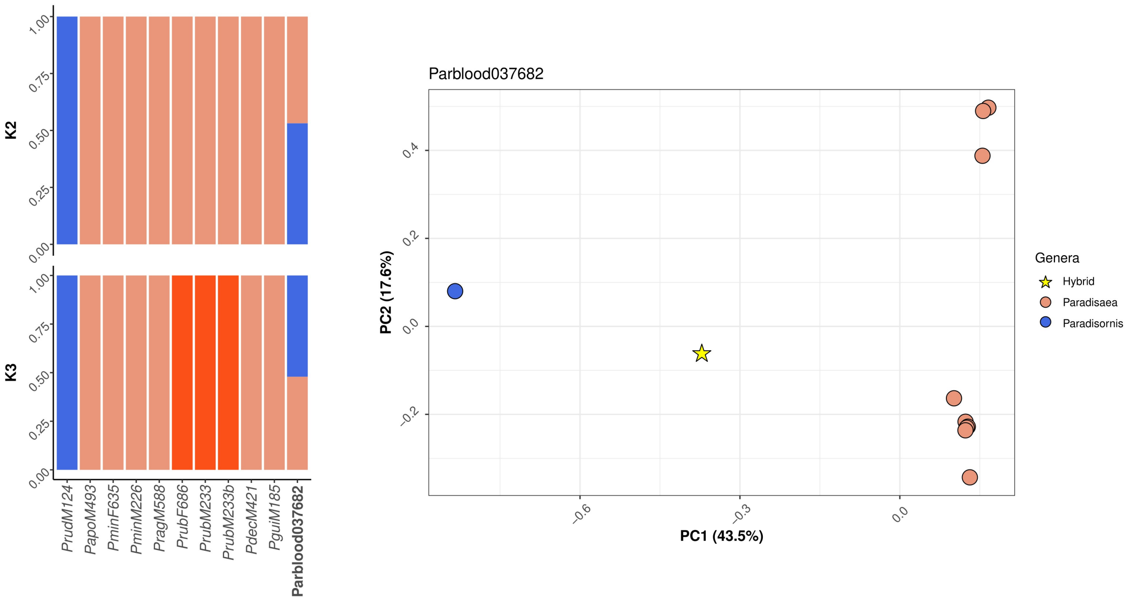

FigureS21. PCA and admixture analysis K=2..3 confirming parental genera implemented through PCAngsd and NGSAdmix. Hybrid is marked with a yellow star in the PCA plot. Samples marked with index 13 in table S2 were used in combination with the hybrid Parblood037682 to produce this plot.

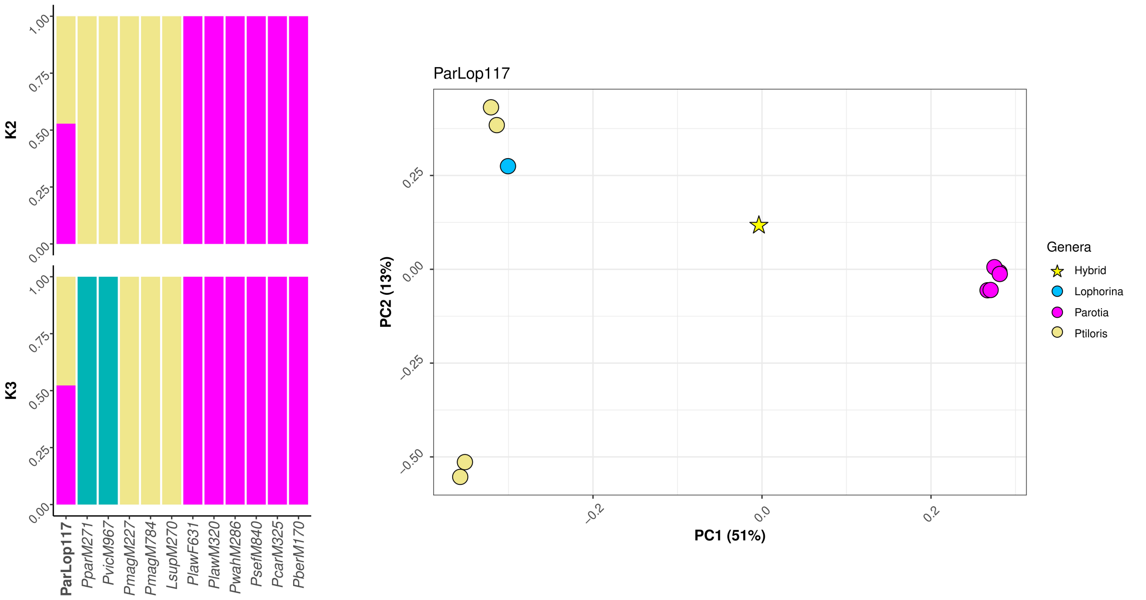

FigureS22. PCA and admixture analysis K=2..3 confirming parental genera implemented through PCAngsd and NGSAdmix. Hybrid is marked with a yellow star in the PCA plot. Samples marked with index 12 in table S2 were used in combination with the hybrid ParLop117 to produce this plot.

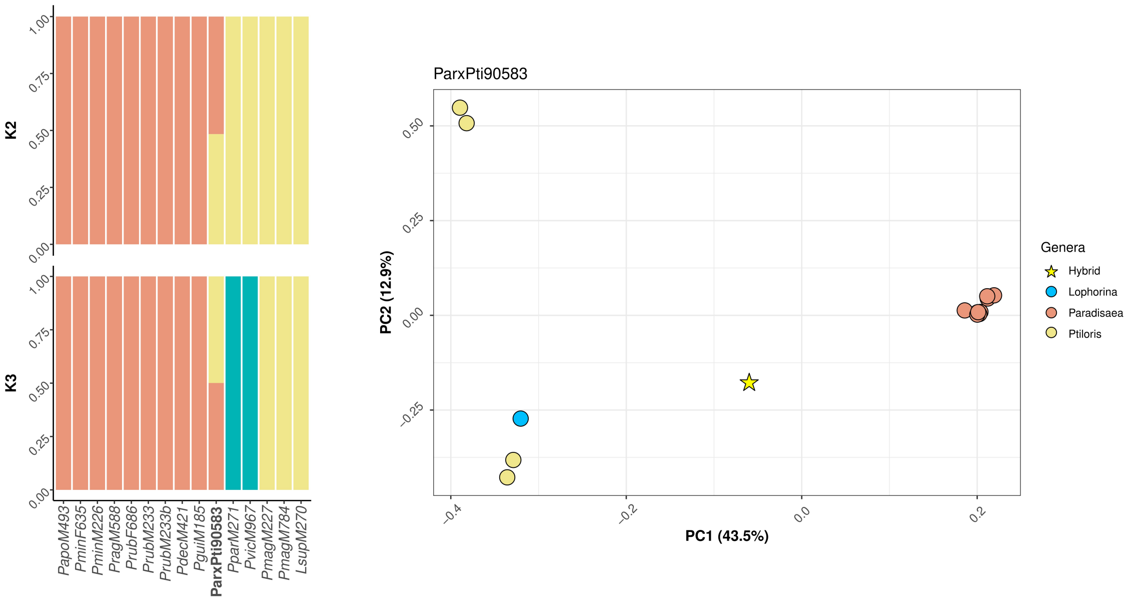

FigureS23. PCA and admixture analysis K=2..3 confirming parental genera implemented through PCAngsd and NGSAdmix. Hybrid is marked with a yellow star in the PCA plot. Samples marked with index 14 in table S2 were used in combination with the hybrid ParxPti90583 to produce this plot.

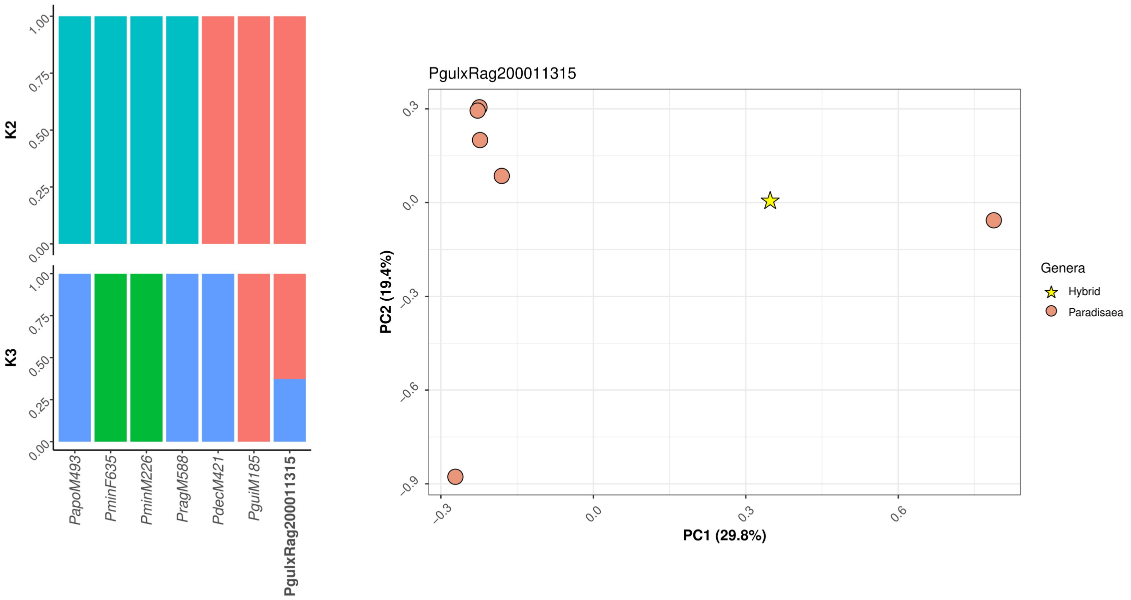

FigureS24. PCA and admixture analysis K=2..3 confirming parental genera implemented through PCAngsd and NGSAdmix. Hybrid is marked with a yellow star in the PCA plot. Samples marked with index 15 in table S2 were used in combination with the hybrid PgulxRag200011315 to produce this plot.

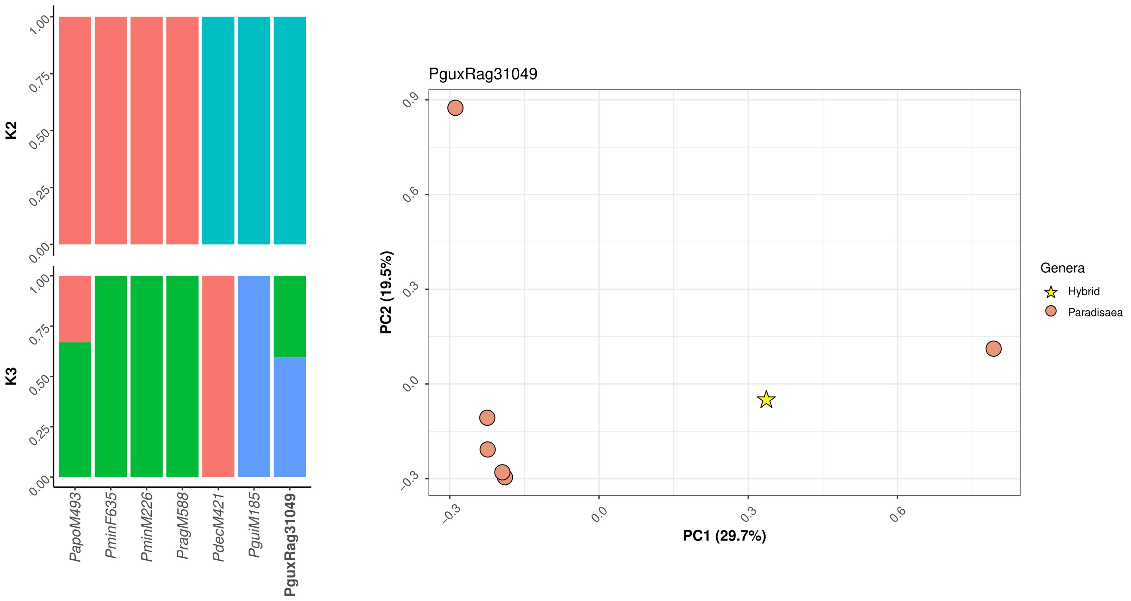

FigureS25. PCA and admixture analysis K=2..3 confirming parental genera implemented through PCAngsd and NGSAdmix. Hybrid is marked with a yellow star in the PCA plot. Samples marked with index 15 in table S2 were used in combination with the hybrid PguxRag31049 to produce this plot.

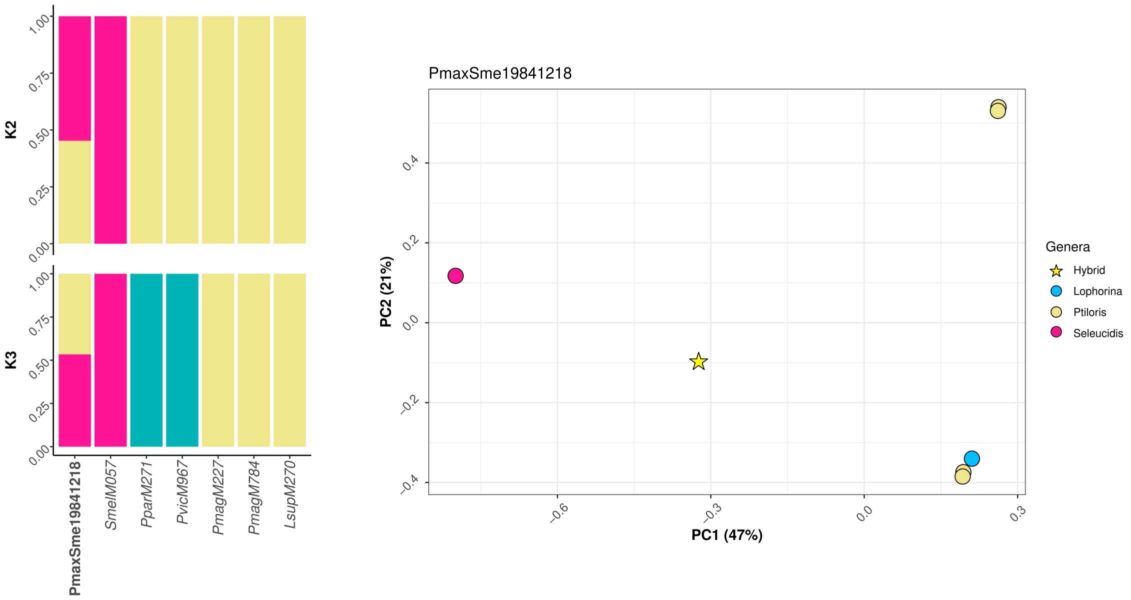

FigureS26. PCA and admixture analysis K=2..3 confirming parental genera implemented through PCAngsd and NGSAdmix. Hybrid is marked with a yellow star in the PCA plot. Samples marked with index 6 in table S2 were used in combination with the hybrid PmaxSme19841218 to produce this plot.

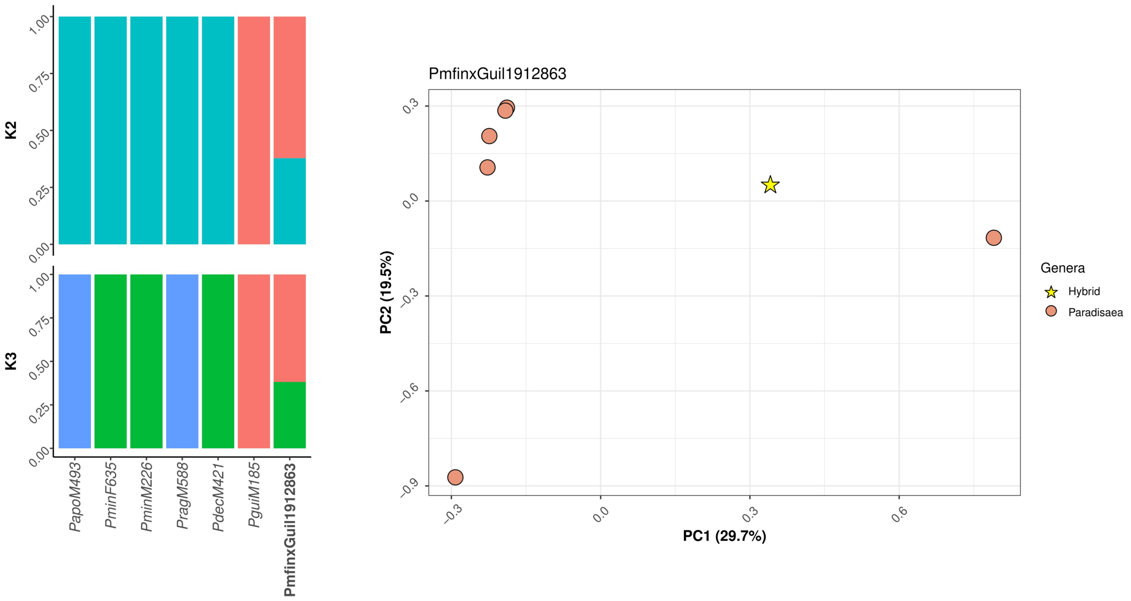

FigureS27. PCA and admixture analysis K=2..3 confirming parental genera implemented through PCAngsd and NGSAdmix. Hybrid is marked with a yellow star in the PCA plot. Samples marked with index 15 in table S2 were used in combination with the hybrid PmfinxGuil1912863 to produce this plot.

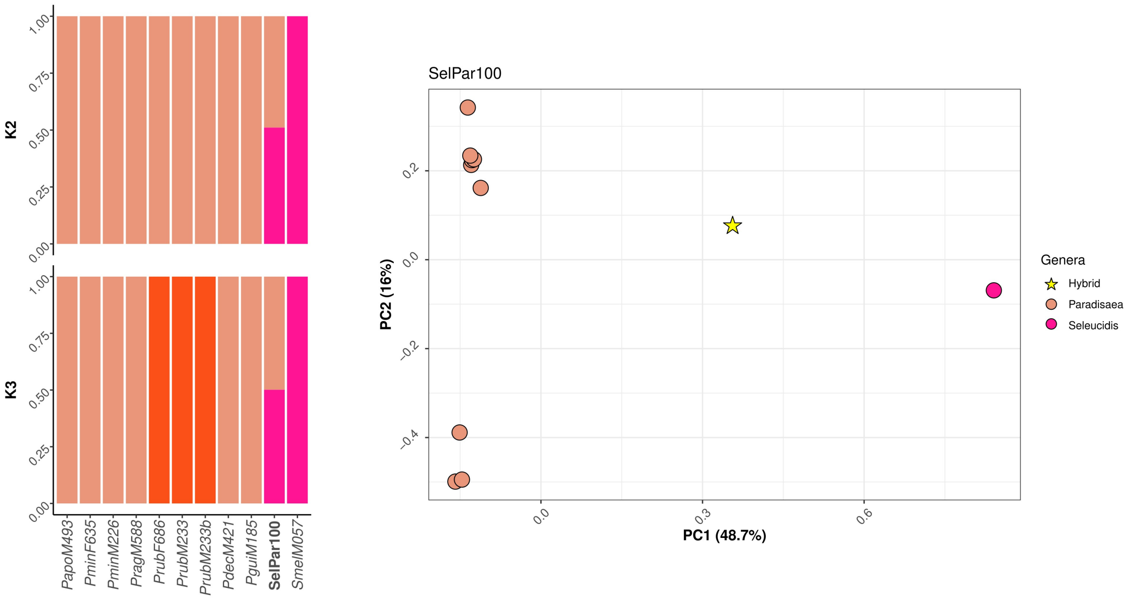

FigureS28. PCA and admixture analysis K=2..3 confirming parental genera implemented through PCAngsd and NGSAdmix. Hybrid is marked with a yellow star in the PCA plot. Samples marked with index 16 in table S2 were used in combination with the hybrid SelPar100 to produce this plot.

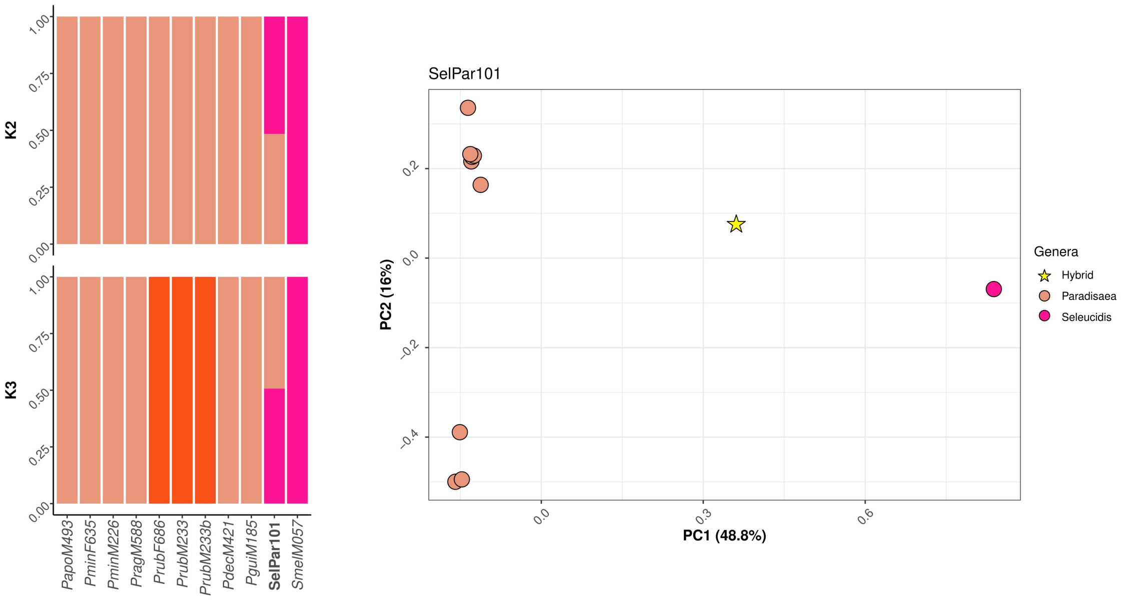

FigureS29. PCA and admixture analysis K=2..3 confirming parental genera implemented through PCAngsd and NGSAdmix. Hybrid is marked with a yellow star in the PCA plot. Samples marked with index 16 in table S2 were used in combination with the hybrid SelPar101 to produce this plot.

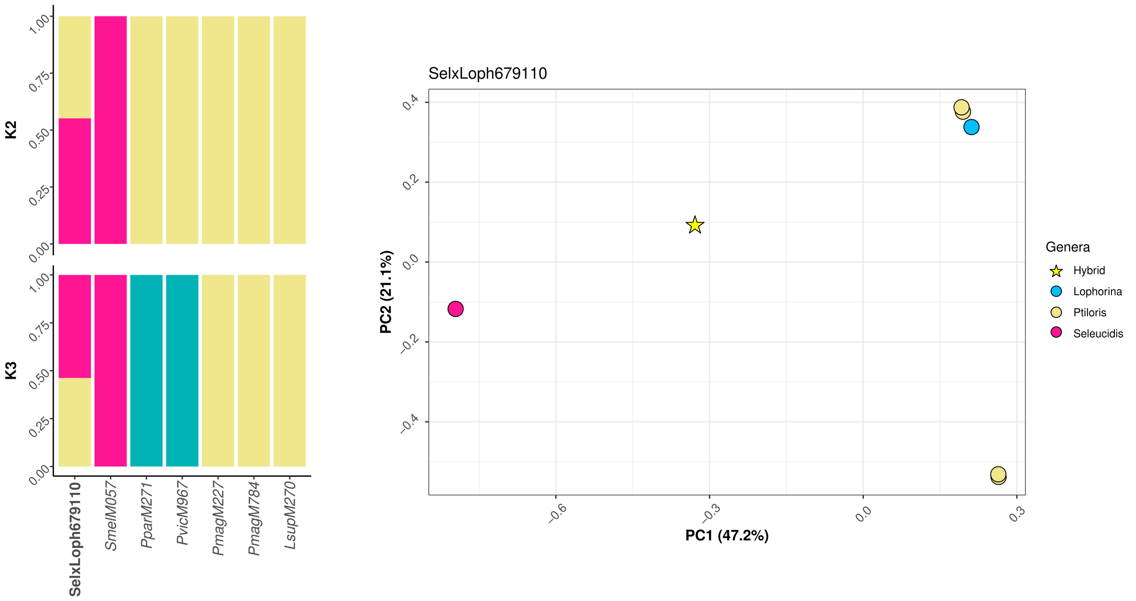

FigureS30. PCA and admixture analysis K=2..3 confirming parental genera implemented through PCAngsd and NGSAdmix. Hybrid is marked with a yellow star in the PCA plot. Samples marked with index 6 in table S2 were used in combination with the hybrid SelxLoph679110 to produce this plot.

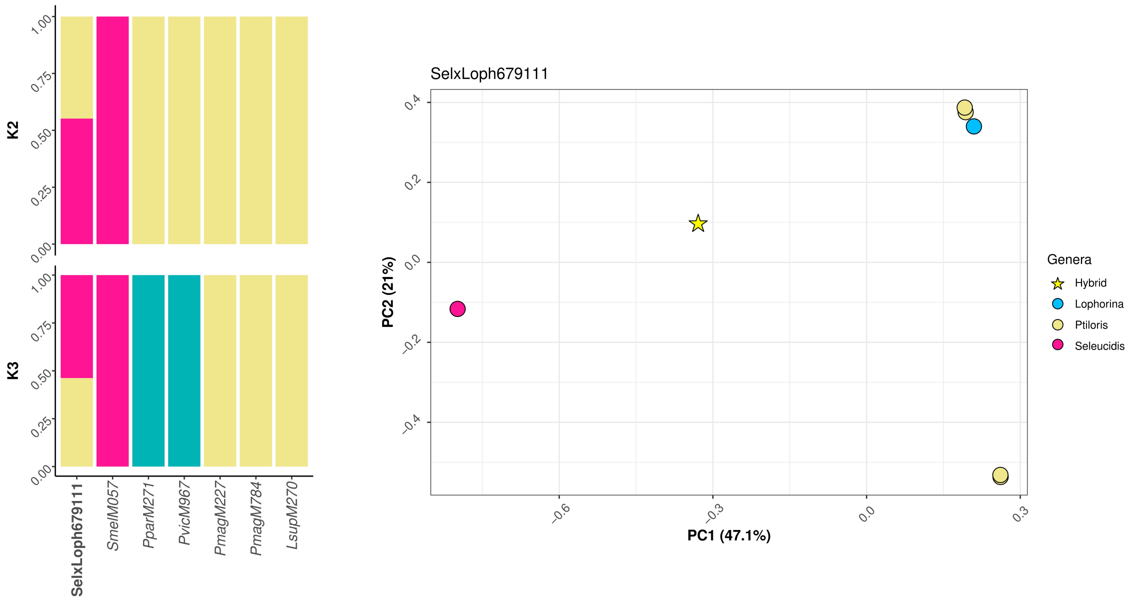

FigureS31. PCA and admixture analysis K=2..3 confirming parental genera implemented through PCAngsd and NGSAdmix. Hybrid is marked with a yellow star in the PCA plot. Samples marked with index 6 in table S2 were used in combination with the hybrid SelxLoph679111 to produce this plot.

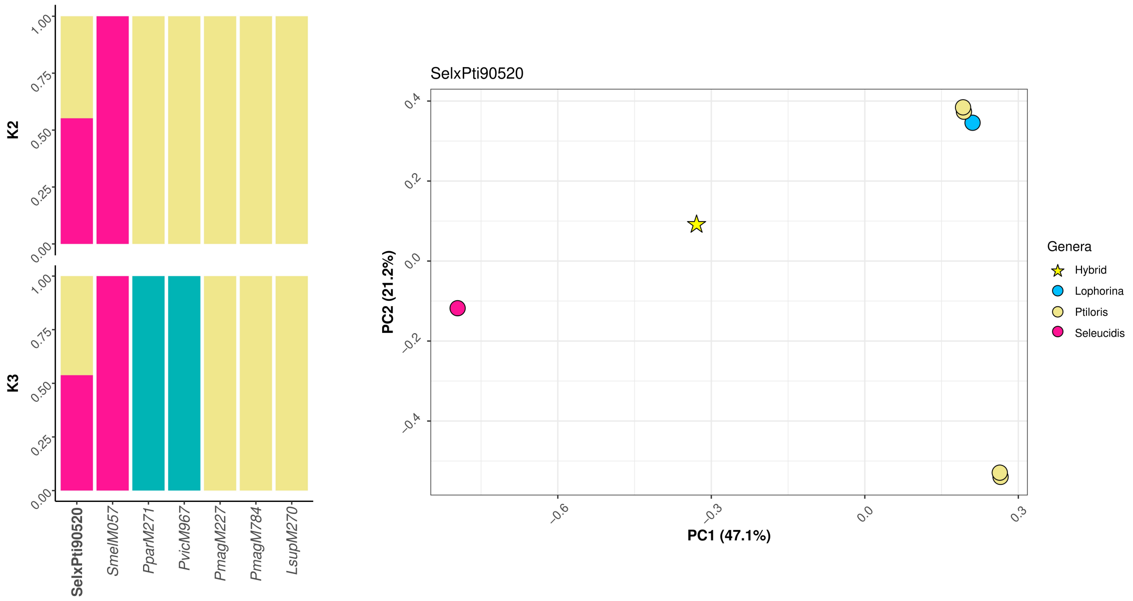

FigureS32. PCA and admixture analysis K=2..3 confirming parental genera implemented through PCAngsd and NGSAdmix. Hybrid is marked with a yellow star in the PCA plot. Samples marked with index 6 in table S2 were used in combination with the hybrid SelxPti90520 to produce this plot.

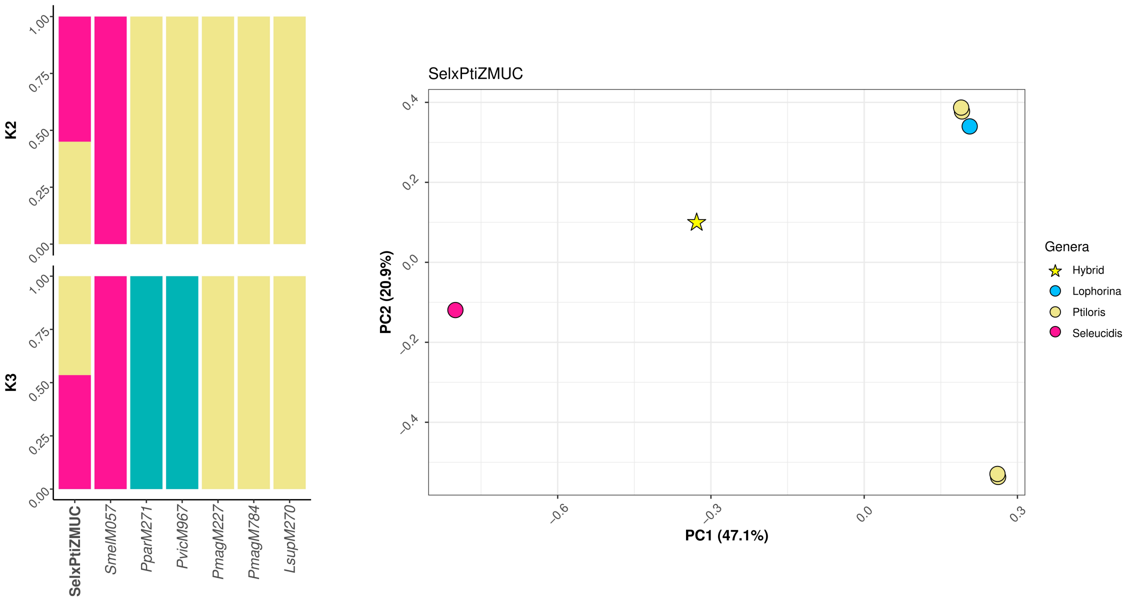

FigureS33. PCA and admixture analysis K=2..3 confirming parental genera implemented through PCAngsd and NGSAdmix. Hybrid is marked with a yellow star in the PCA plot. Samples marked with index 6 in table S2 were used in combination with the hybrid SelxPtiZMUC to produce this plot.

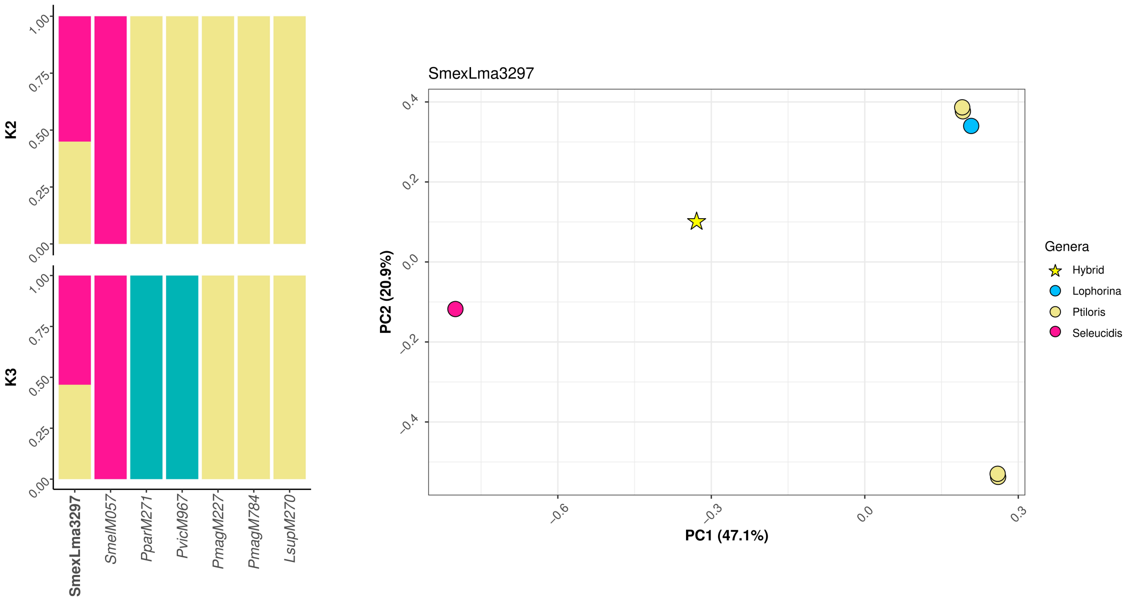

FigureS34. PCA and admixture analysis K=2..3 confirming parental genera implemented through PCAngsd and NGSAdmix. Hybrid is marked with a yellow star in the PCA plot. Samples marked with index 6 in table S2 were used in combination with the hybrid SmexLma3297 to produce this plot.

Ancestry Informative Markers

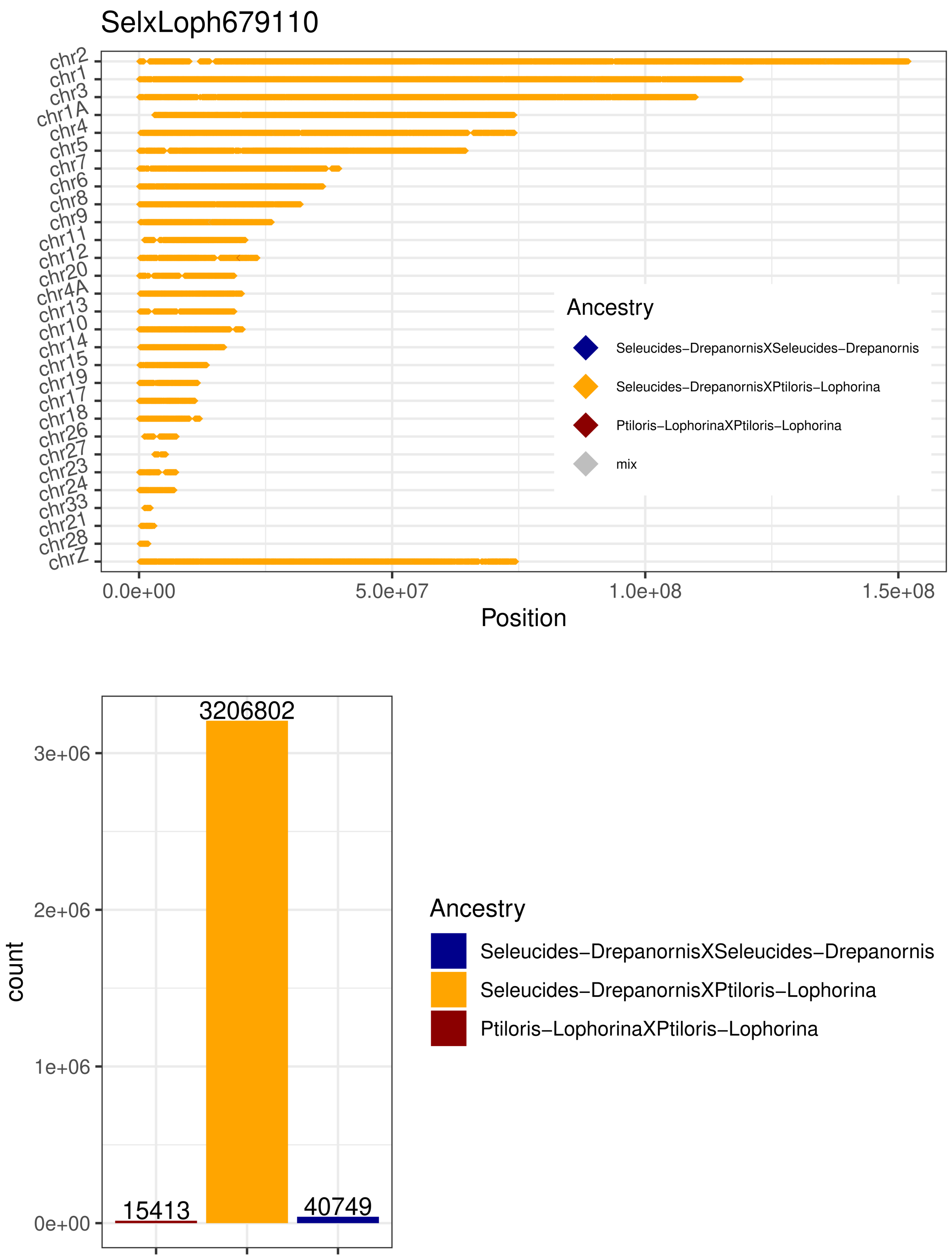

FigureS35. AIMs in bins of 100 positions along autosomes and Z chromosome in SelxLoph679110 indicating its F1-hybrid status. Counts of homozygous and heterozygous AIMs in SelxLoph679110 indicating its F1-hybrid status. Samples marked with index 19 in table S2 were used to produce this plot.

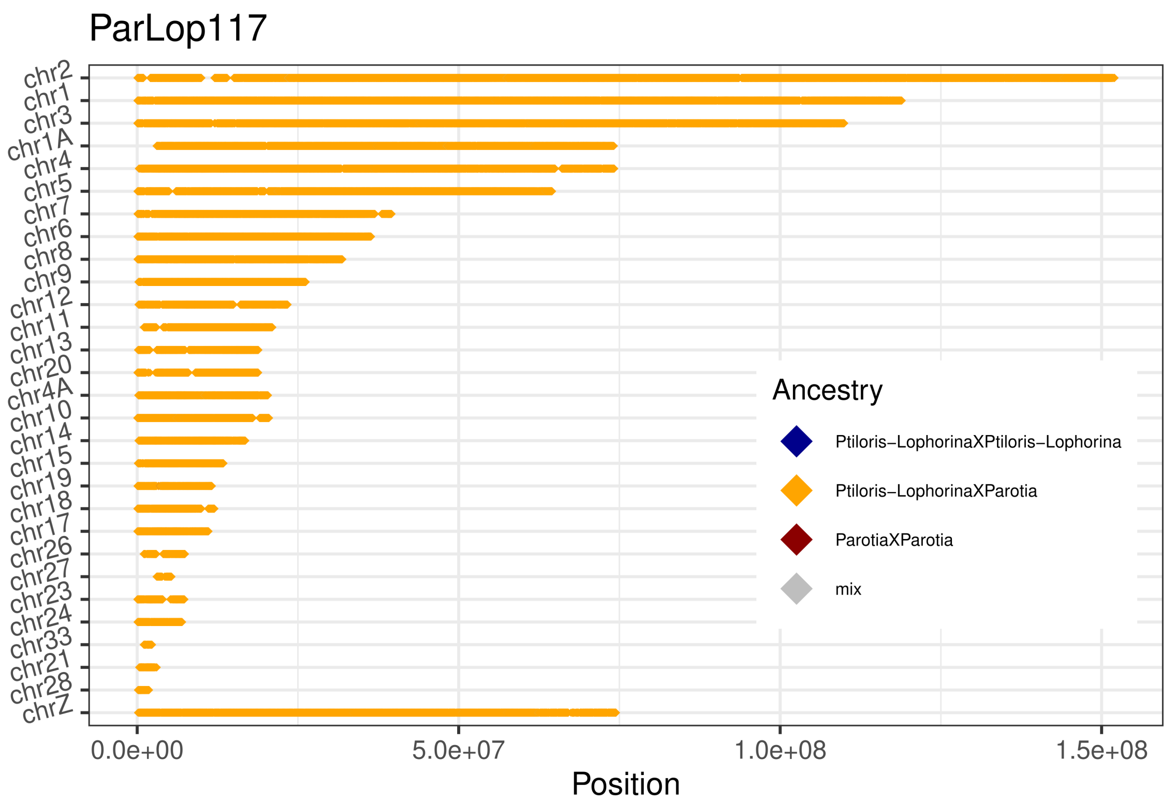

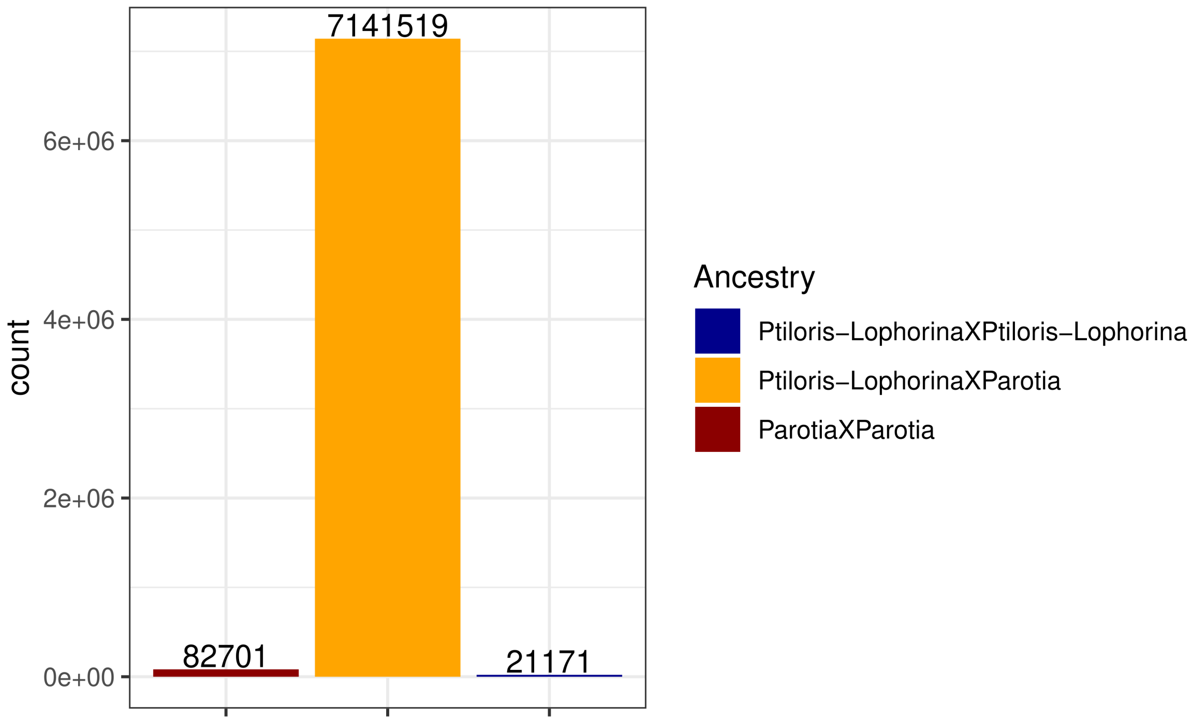

FigureS36. AIMs in bins of 100 positions along autosomes and Z chromosome in ParLop117 indicating its F1-hybrid status. Counts of homozygous and heterozygous AIMs in ParLop117 indicating its F1-hybrid status. Samples marked with index 12 in table S2 were used to produce this plot.

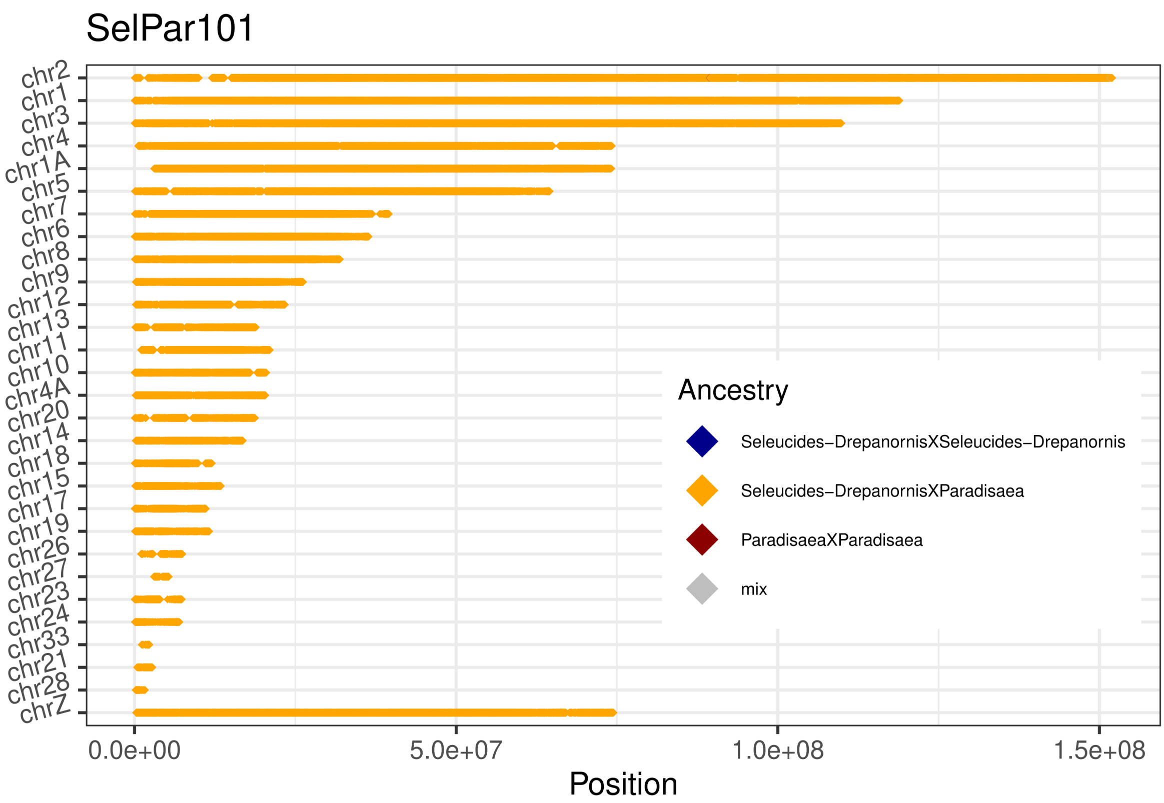

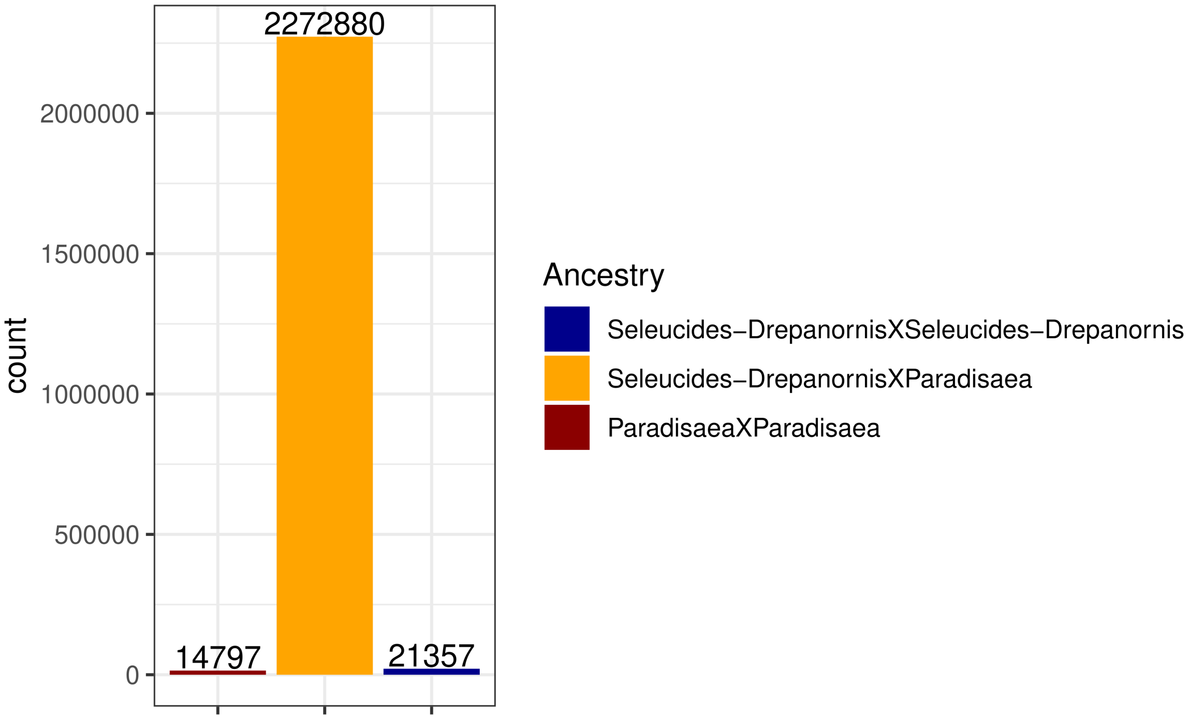

FigureS37. AIMs in bins of 100 positions along autosomes and Z chromosome in SelPar101 indicating its F1-hybrid status. Counts of homozygous and heterozygous AIMs in SelLop101 indicating its F1-hybrid status. Samples marked with index 20 in table S2 were used to produce this plot.

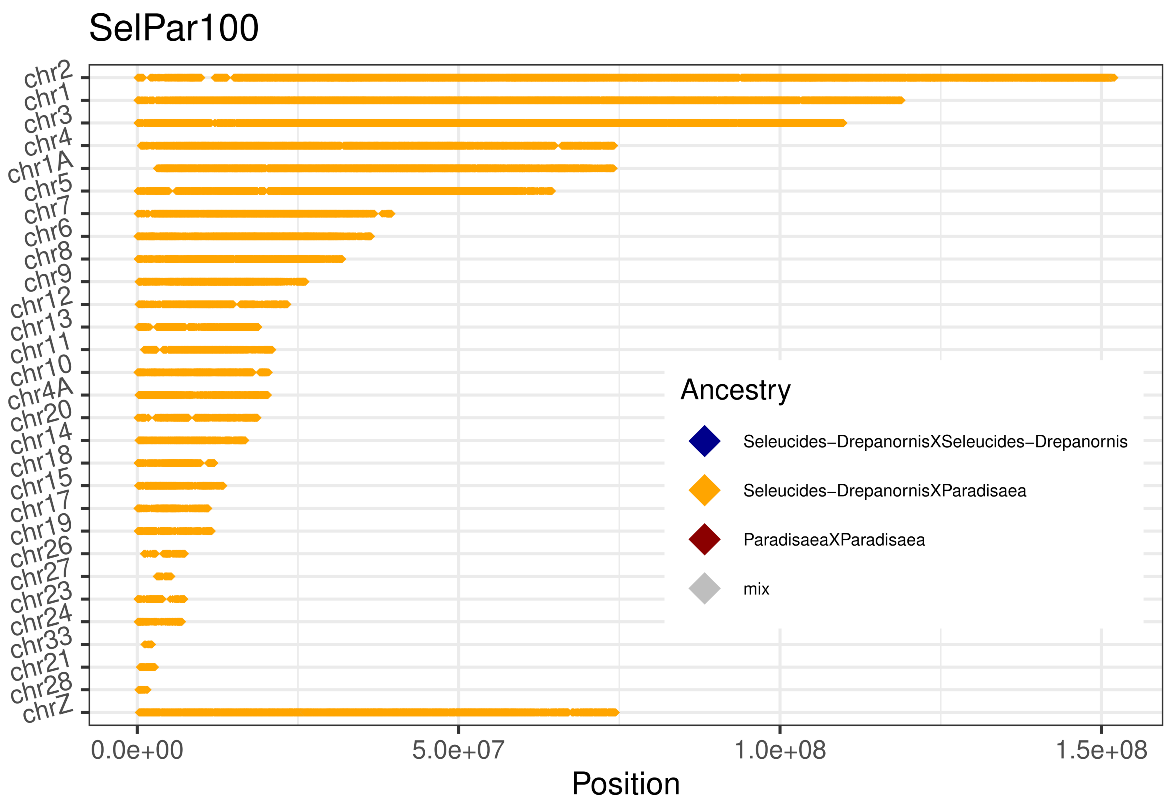

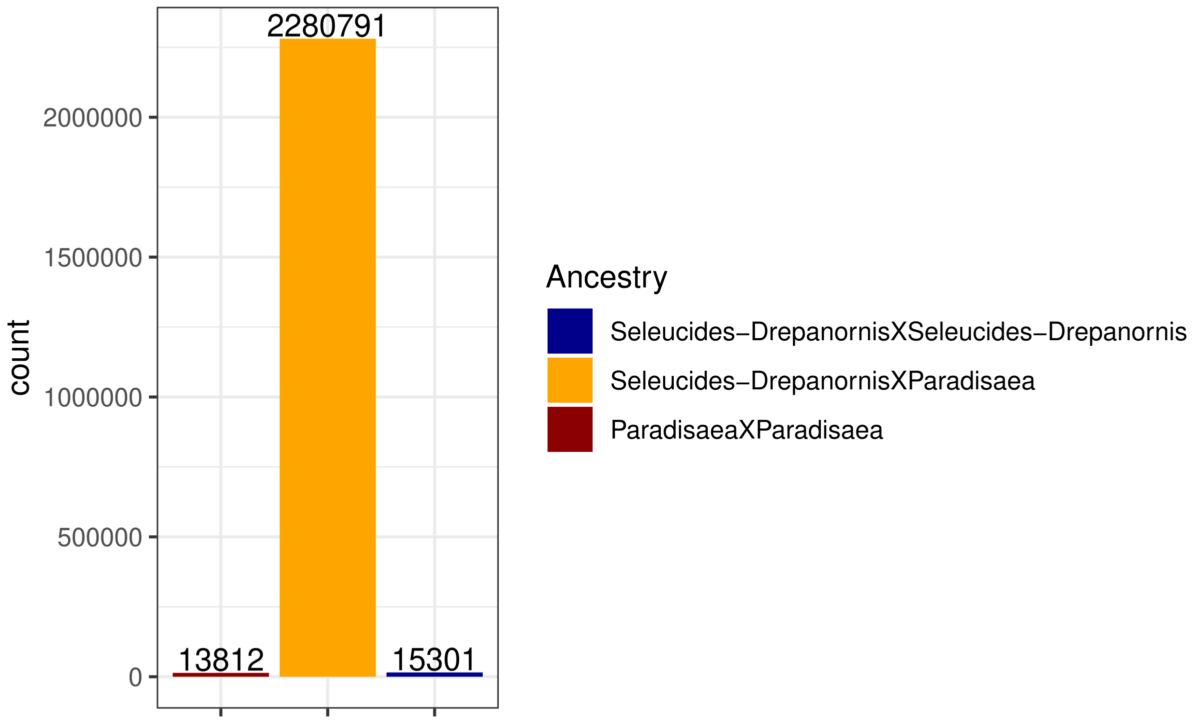

FigureS38. AIMs in bins of 100 positions along autosomes and Z chromosome in SelPar100 indicating its F1-hybrid status. Counts of homozygous and heterozygous AIMs in SelLop100 indicating its F1-hybrid status. Samples marked with index 20 in table S2 were used to produce this plot.

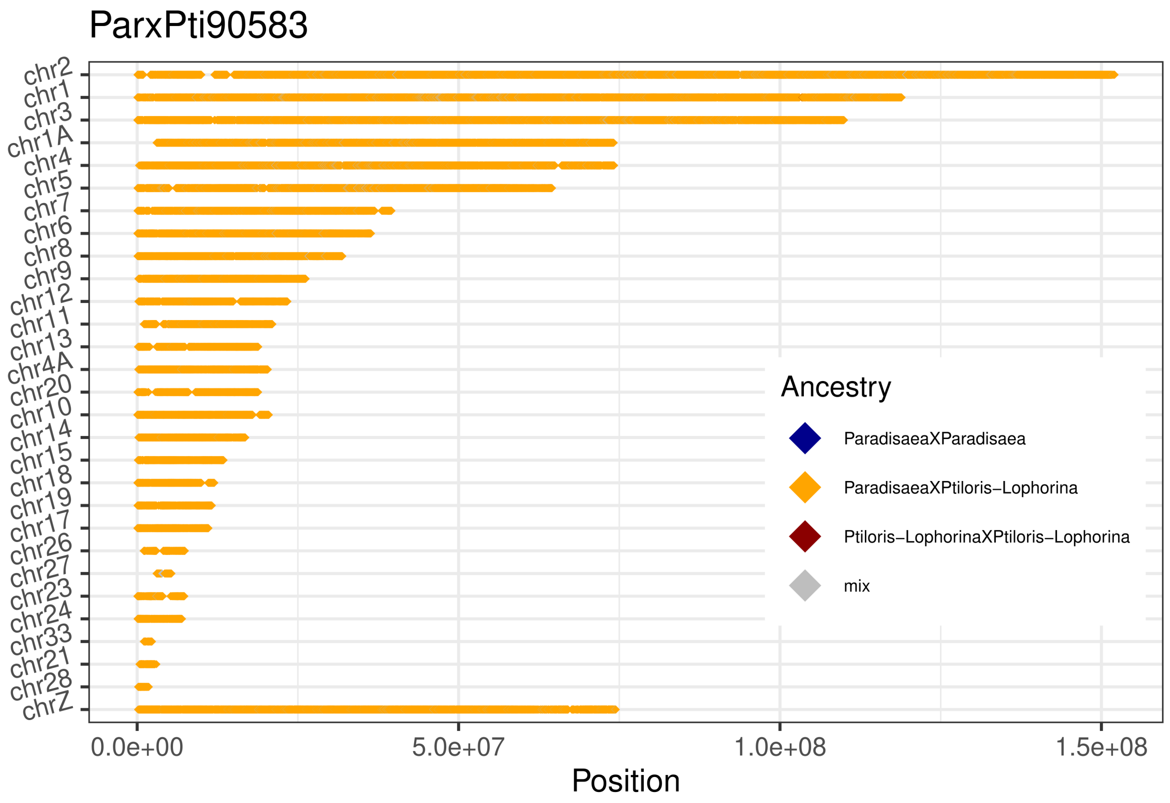

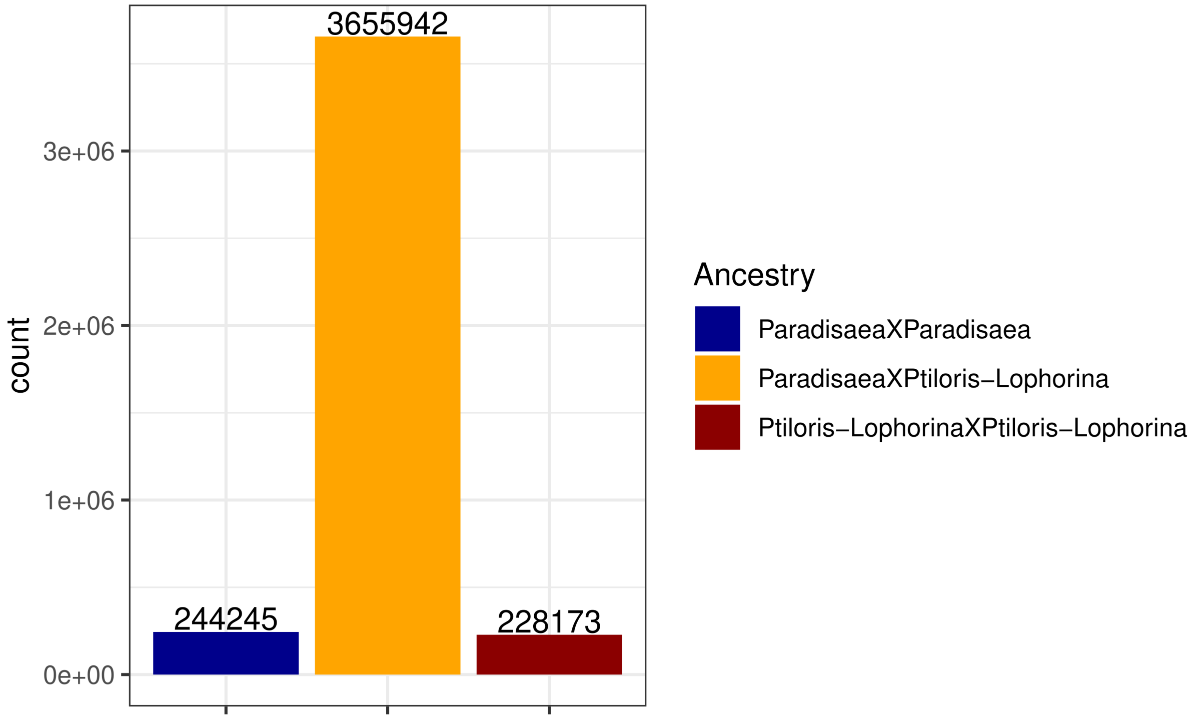

FigureS39. AIMs in bins of 100 positions along autosomes and Z chromosome in ParxPtil90583 indicating its F1-hybrid status. Counts of homozygous and heterozygous AIMs in ParxPtil90583 indicating its F1-hybrid status. Samples marked with index 14 in table S2 were used to produce this plot.

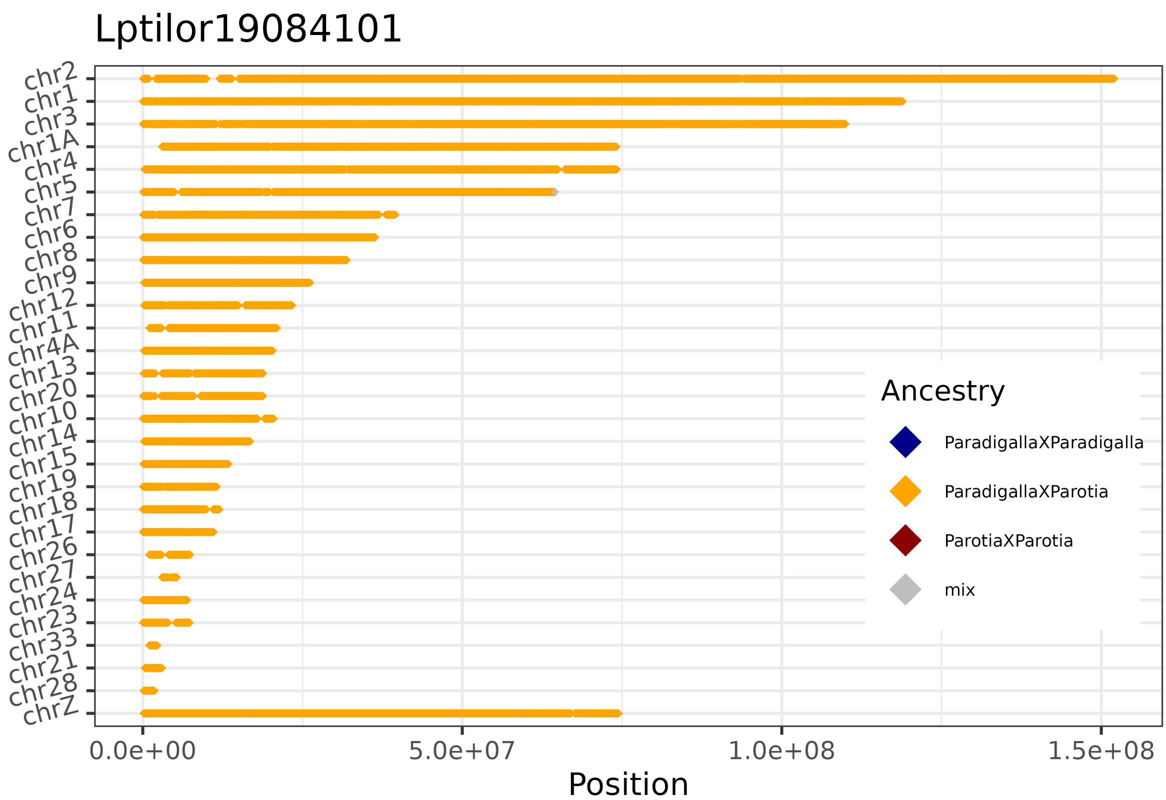

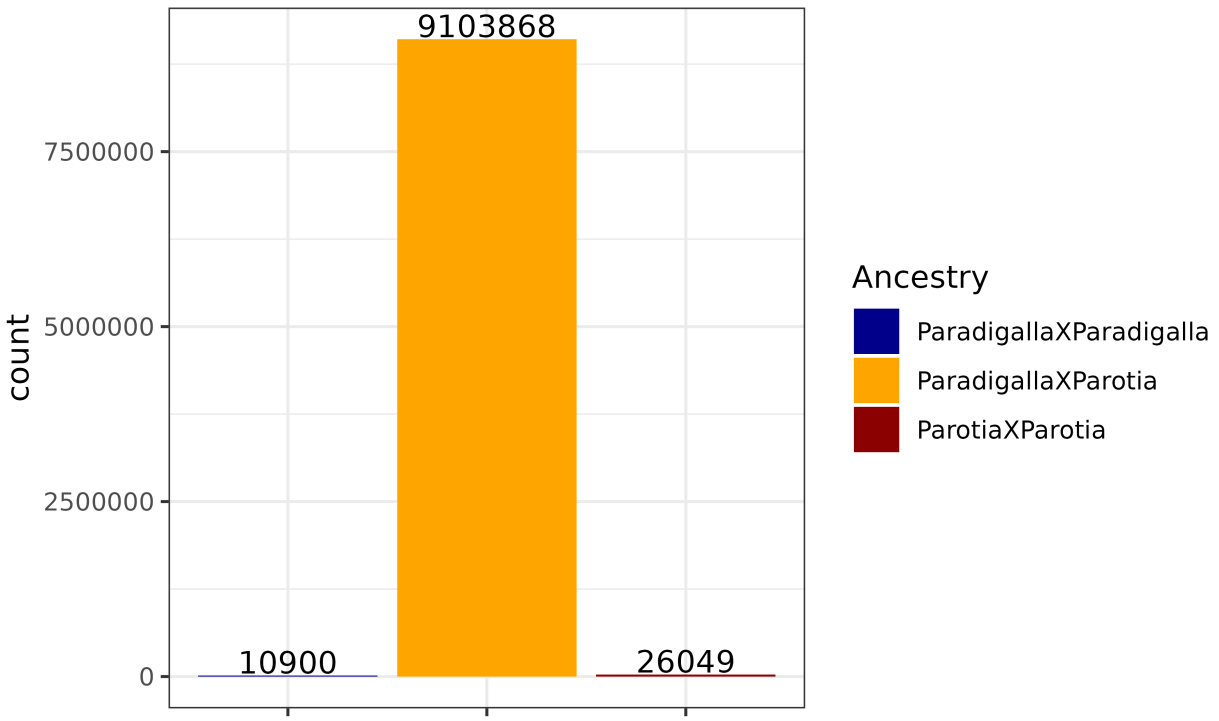

FigureS40. AIMs in bins of 100 positions along autosomes and Z chromosome in Lptilor19084101 indicating its F1-hybrid status. Counts of homozygous and heterozygous AIMs in Lptilor19084101 indicating its F1-hybrid status. Samples marked with index 10 in table S2 were used to produce this plot.

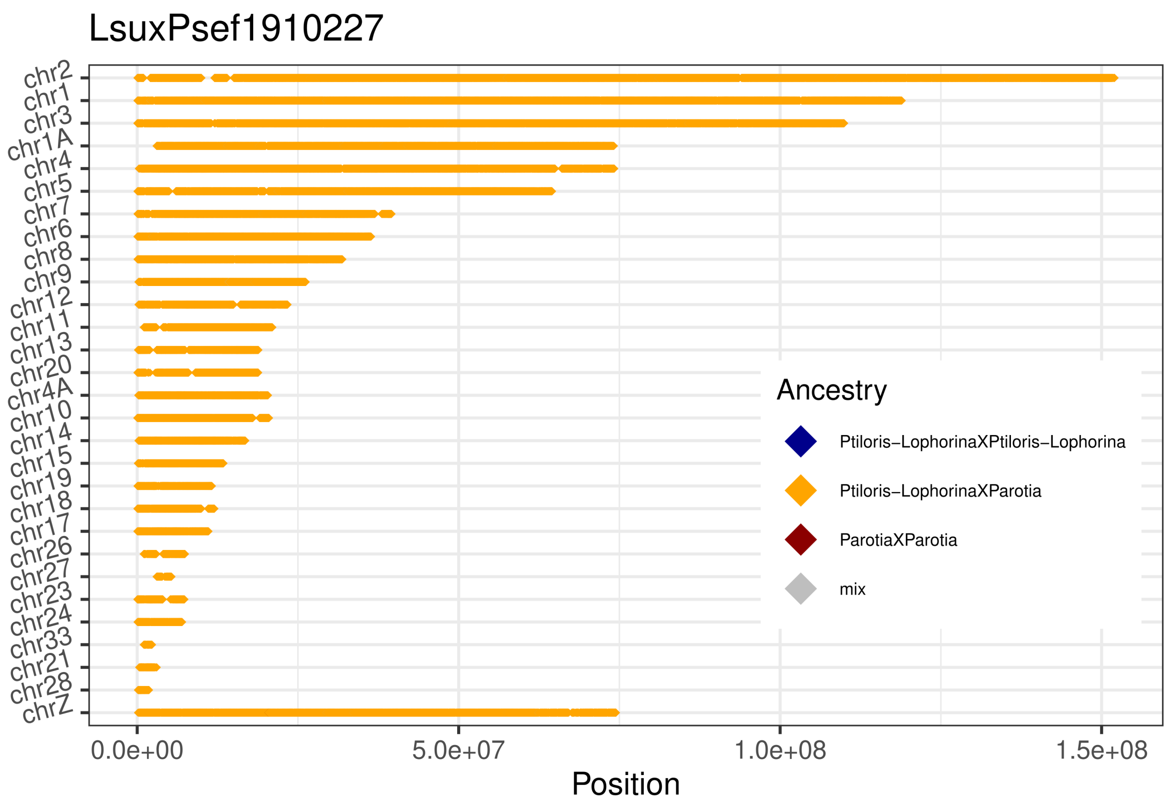

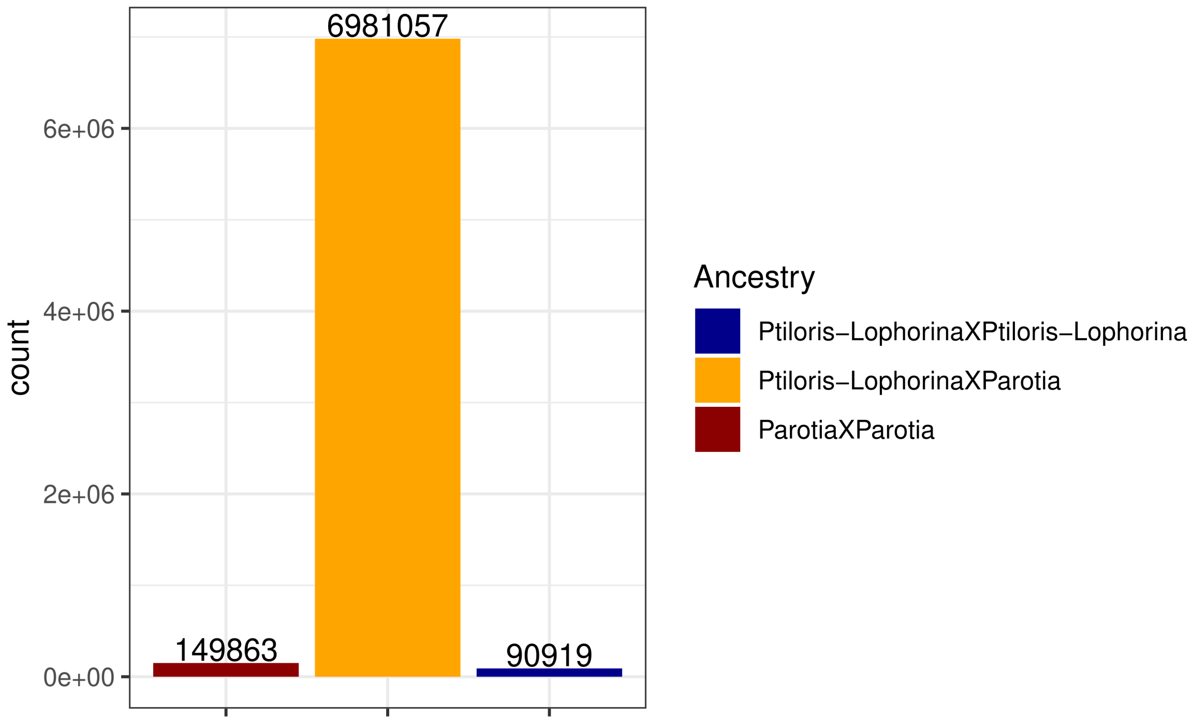

FigureS41. AIMs in bins of 100 positions along autosomes and Z chromosome in LsuxPsef1910227 indicating its F1-hybrid status. Counts of homozygous and heterozygous AIMs in LsuxPsef1910227 indicating its F1-hybrid status. Samples marked with index 12 in table S2 were used to produce this plot.

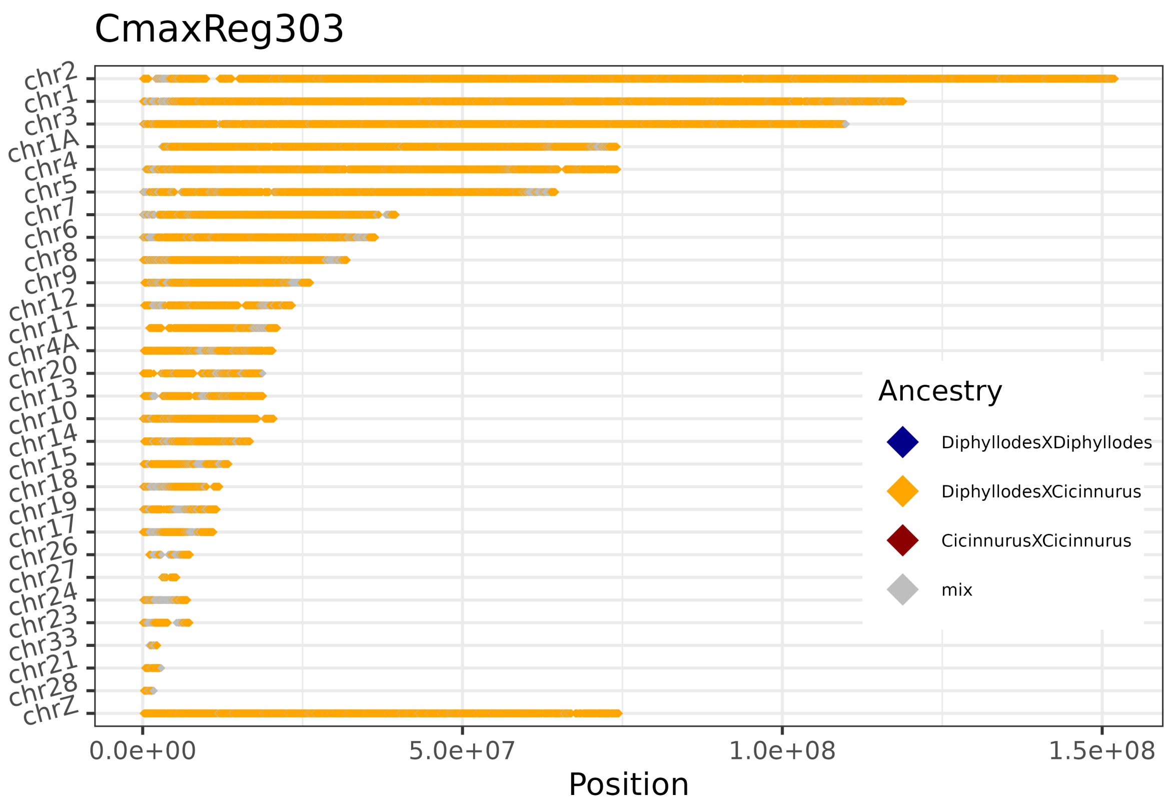

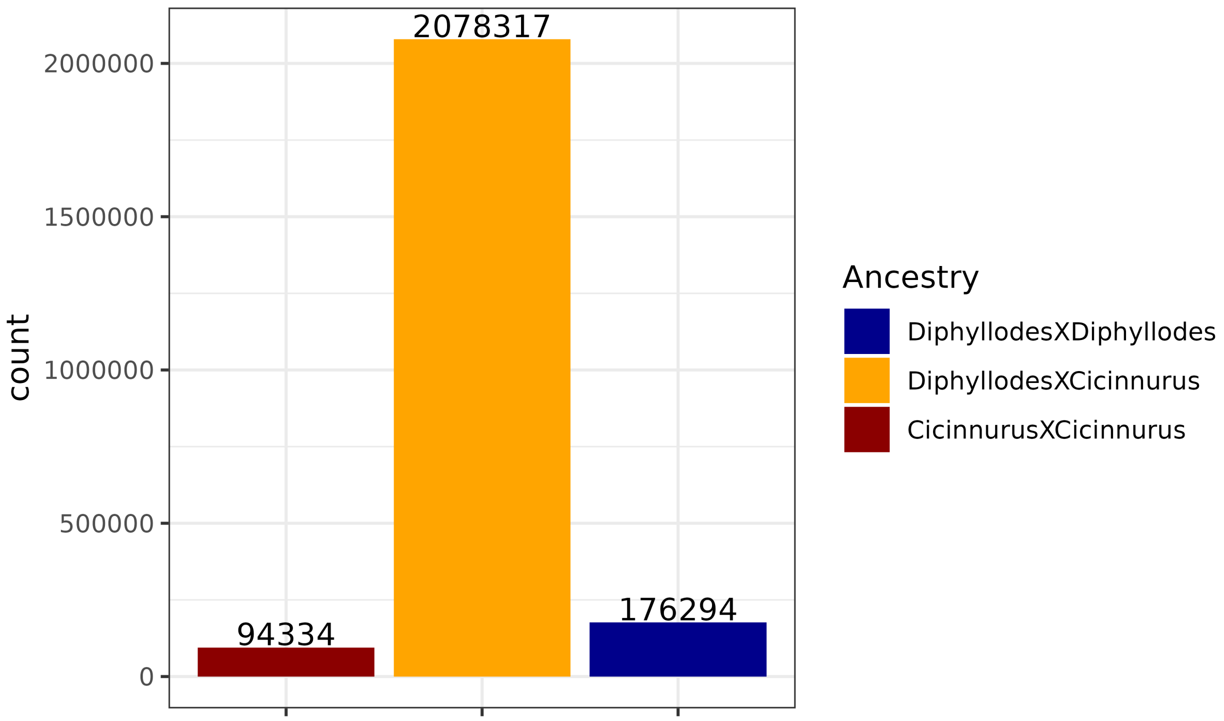

FigureS42. AIMs in bins of 100 positions along autosomes and Z chromosome in CmaxReg303 indicating its F1-hybrid status. Counts of homozygous and heterozygous AIMs in CmaxReg303 indicating its F1-hybrid status. Samples marked with index 3 in table S2 were used to produce this plot.

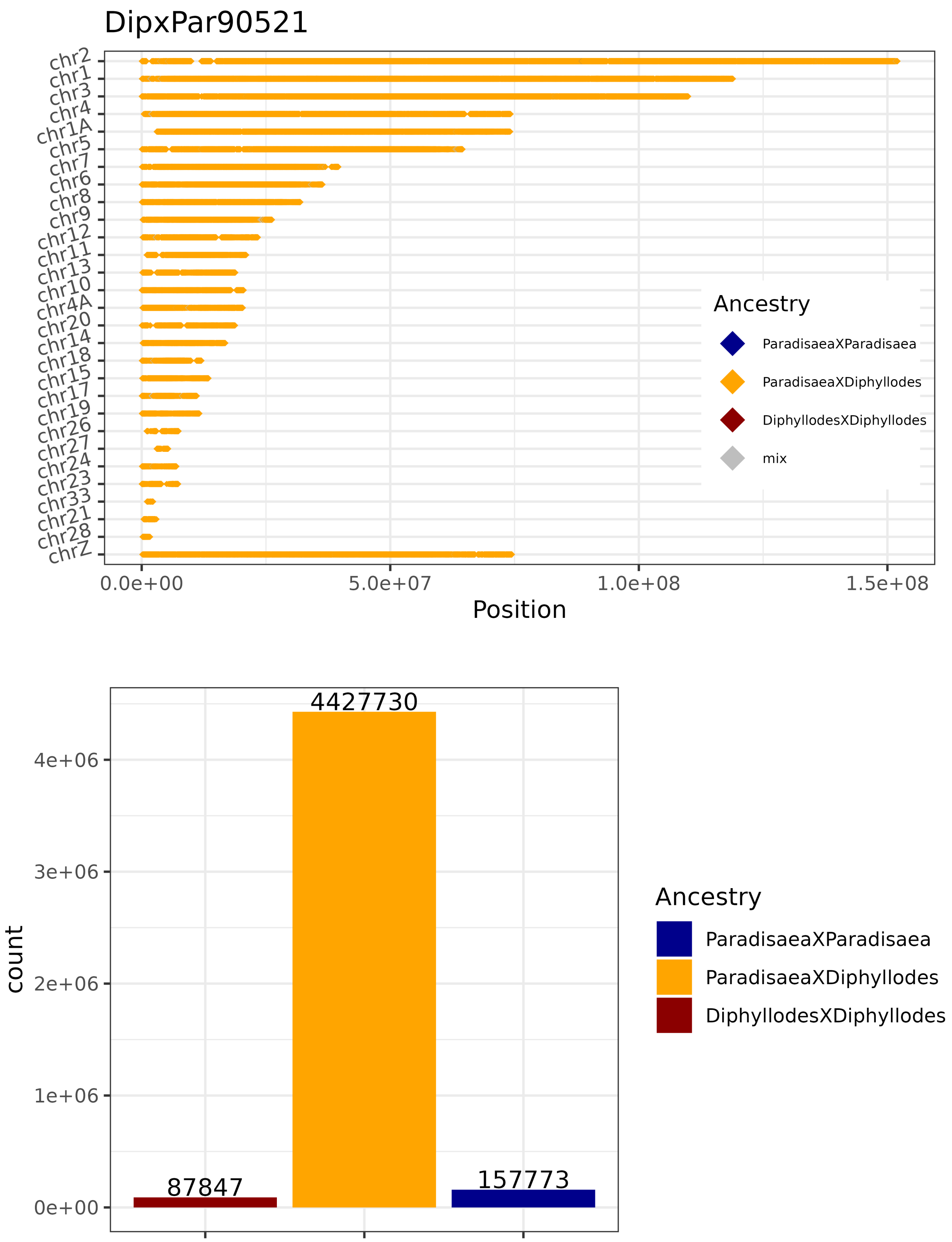

FigureS43. AIMs in bins of 100 positions along autosomes and Z chromosome in DipxPar90521 indicating its F1-hybrid status. Counts of homozygous and heterozygous AIMs in DipxPar90521 indicating its F1-hybrid status. Samples marked with index 3 in table S2 were used to produce this plot.

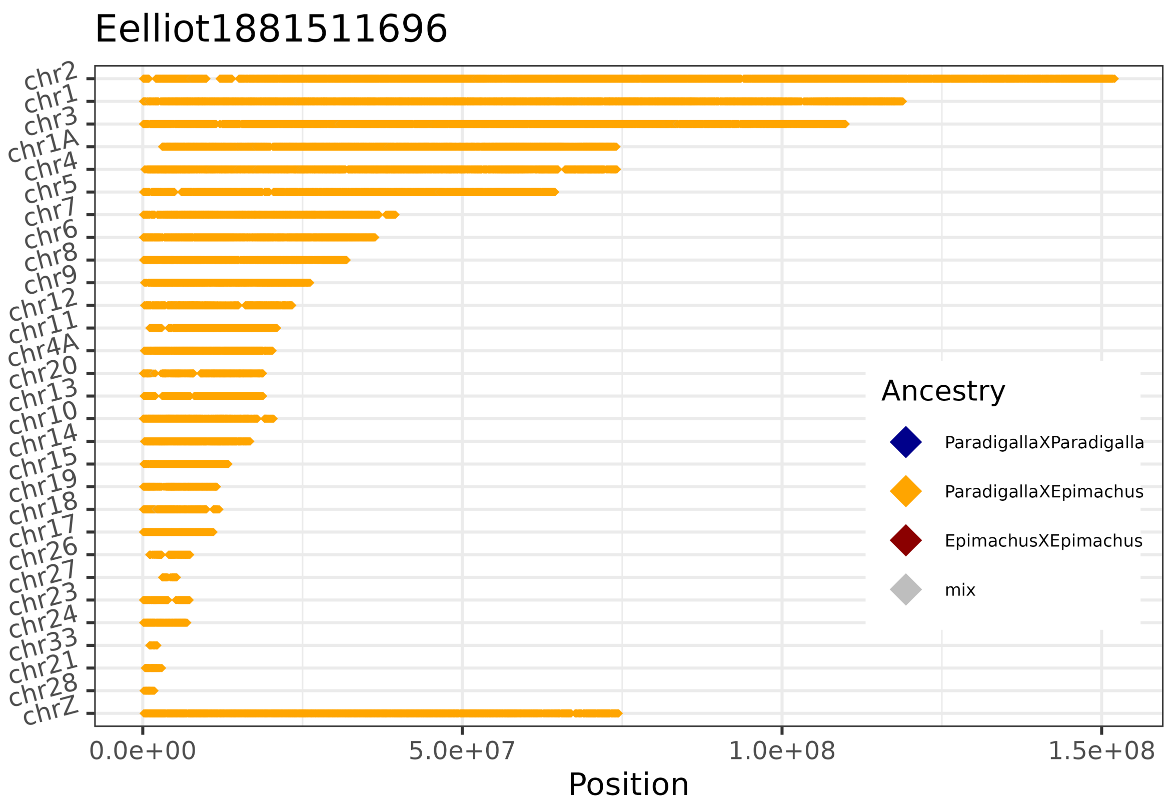

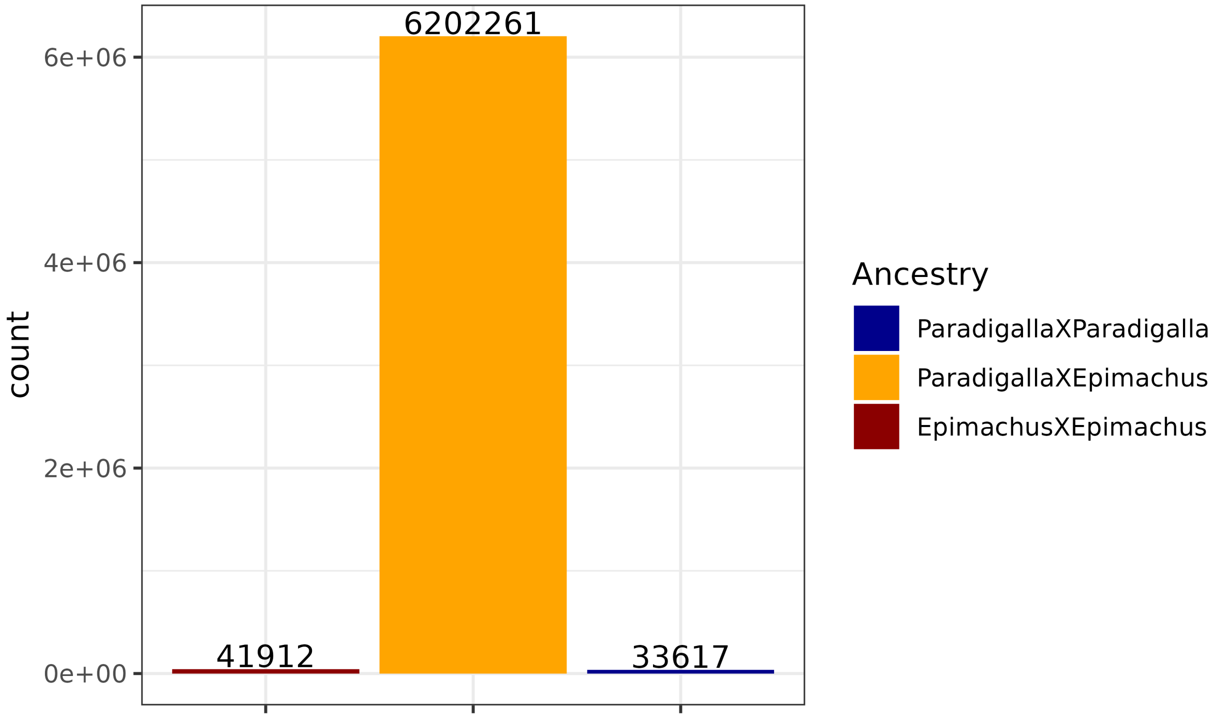

FigureS44. AIMs in bins of 100 positions along autosomes and Z chromosome in Eelliot1881511696 indicating its F1-hybrid status. Counts of homozygous and heterozygous AIMs in Eelliot1881511696 indicating its F1-hybrid status. Samples marked with index 4 in table S2 were used to produce this plot.

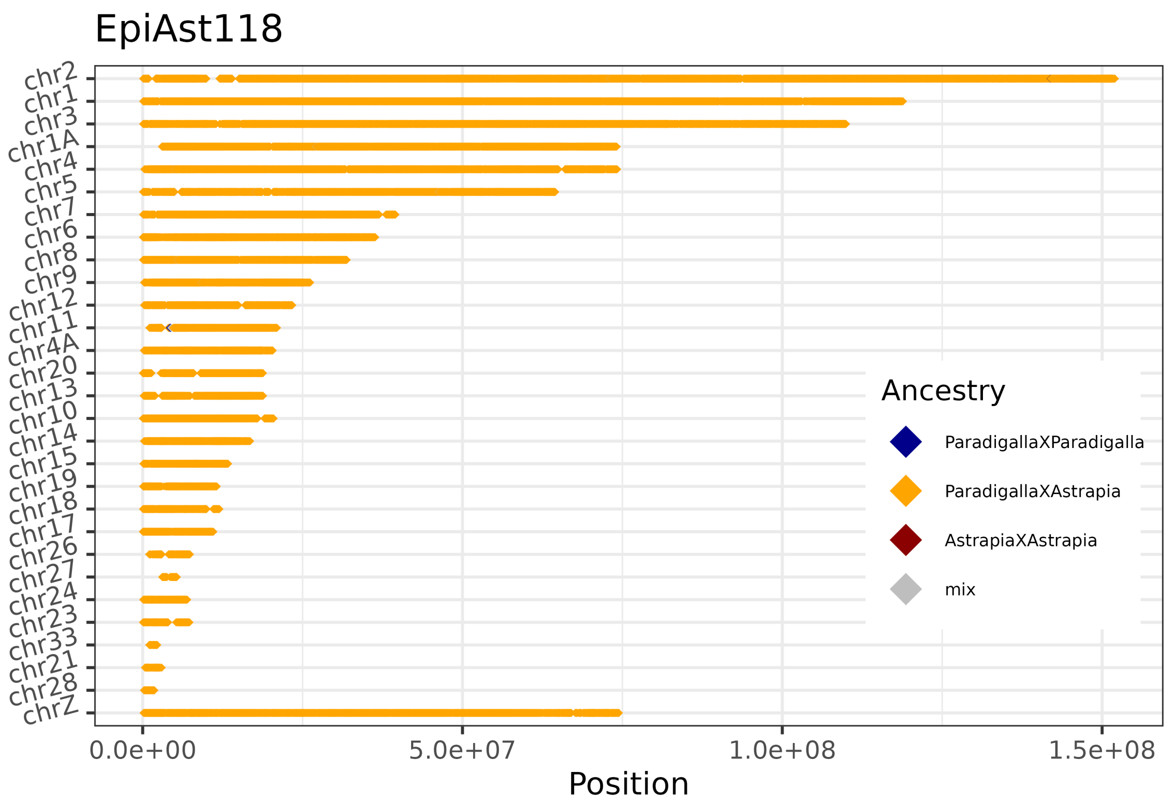

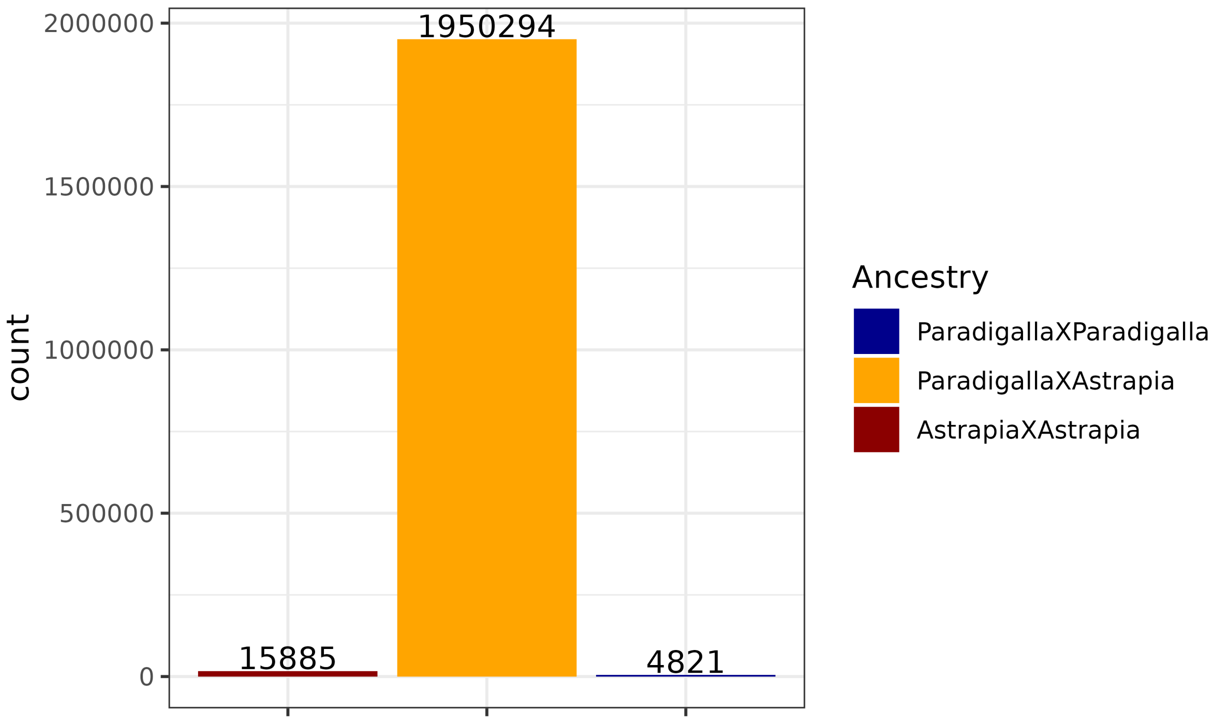

FigureS45. AIMs in bins of 100 positions along autosomes and Z chromosome in EpiAst118 indicating its F1-hybrid status. Counts of homozygous and heterozygous AIMs in EpiAst118 indicating its F1-hybrid status. Samples marked with index 1 in table S2 were used to produce this plot.

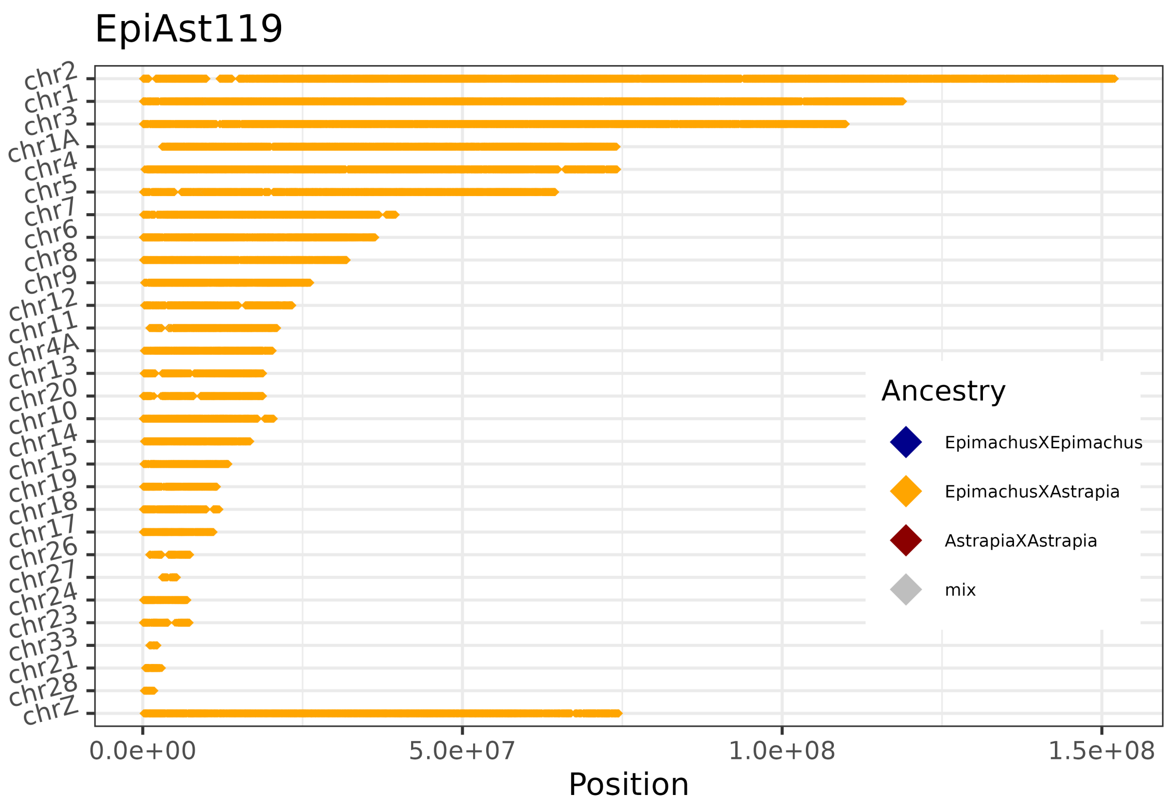

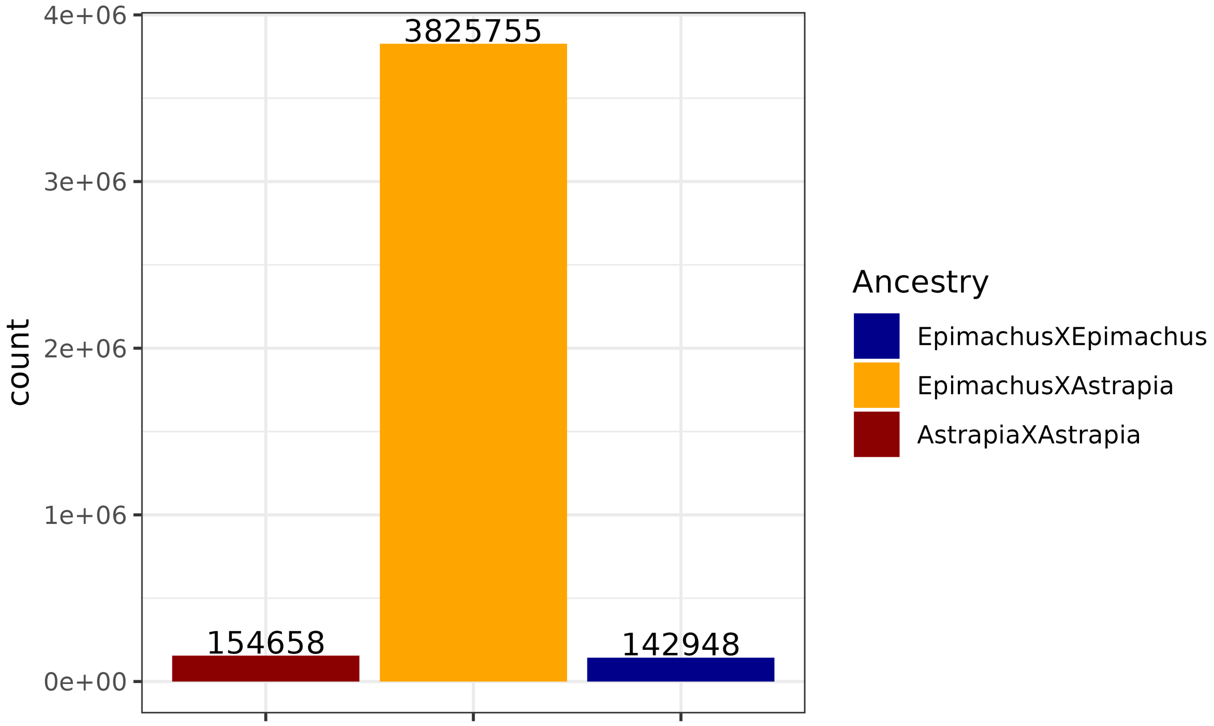

FigureS46. AIMs in bins of 100 positions along autosomes and Z chromosome in EpiAst119 indicating its F1-hybrid status. Counts of homozygous and heterozygous AIMs in EpiAst119 indicating its F1-hybrid status. Samples marked with index 2 in table S2 were used to produce this plot.

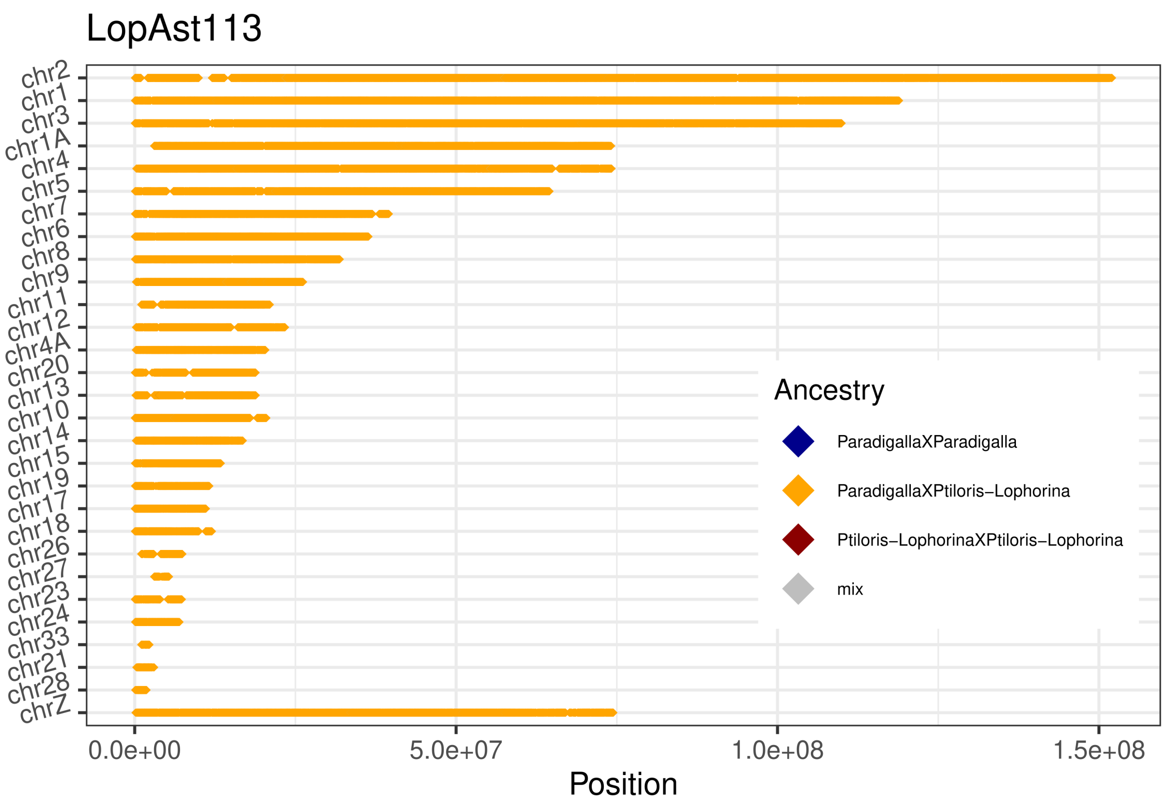

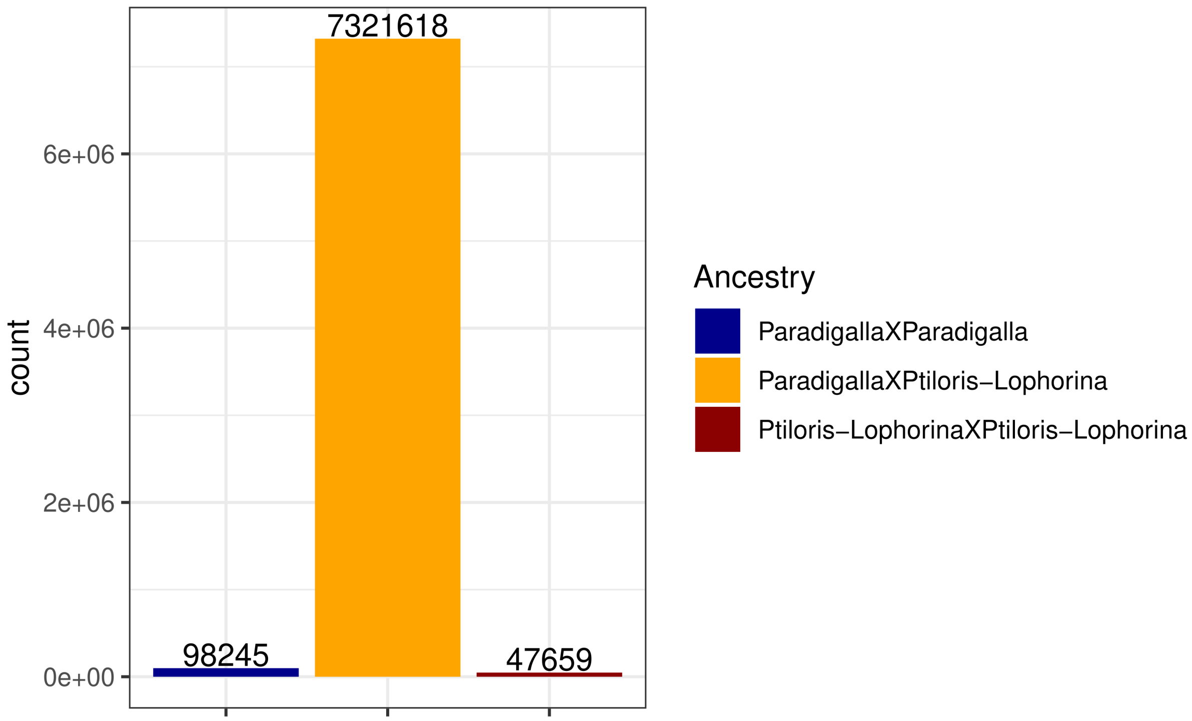

FigureS47. AIMs in bins of 100 positions along autosomes and Z chromosome in LopAst113 indicating its F1-hybrid status. Counts of homozygous and heterozygous AIMs in LopAst113 indicating its F1-hybrid status. Samples marked with index 8 in table S2 were used to produce this plot.

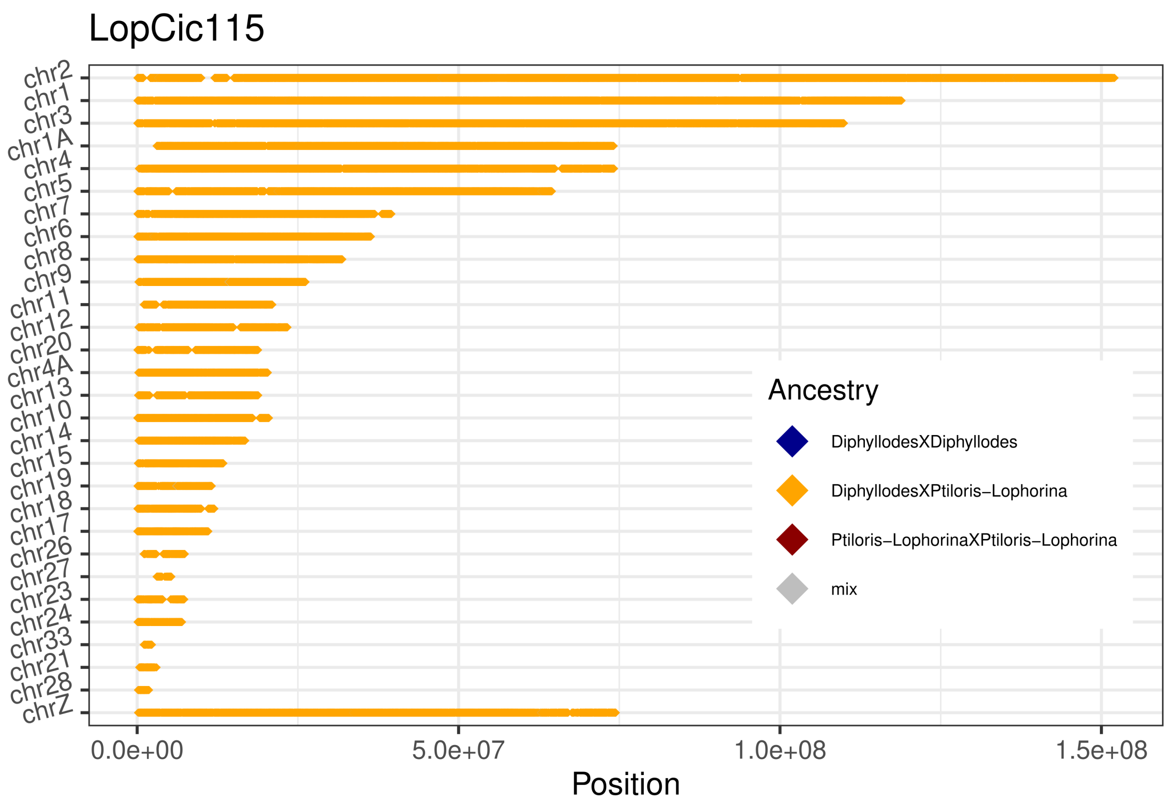

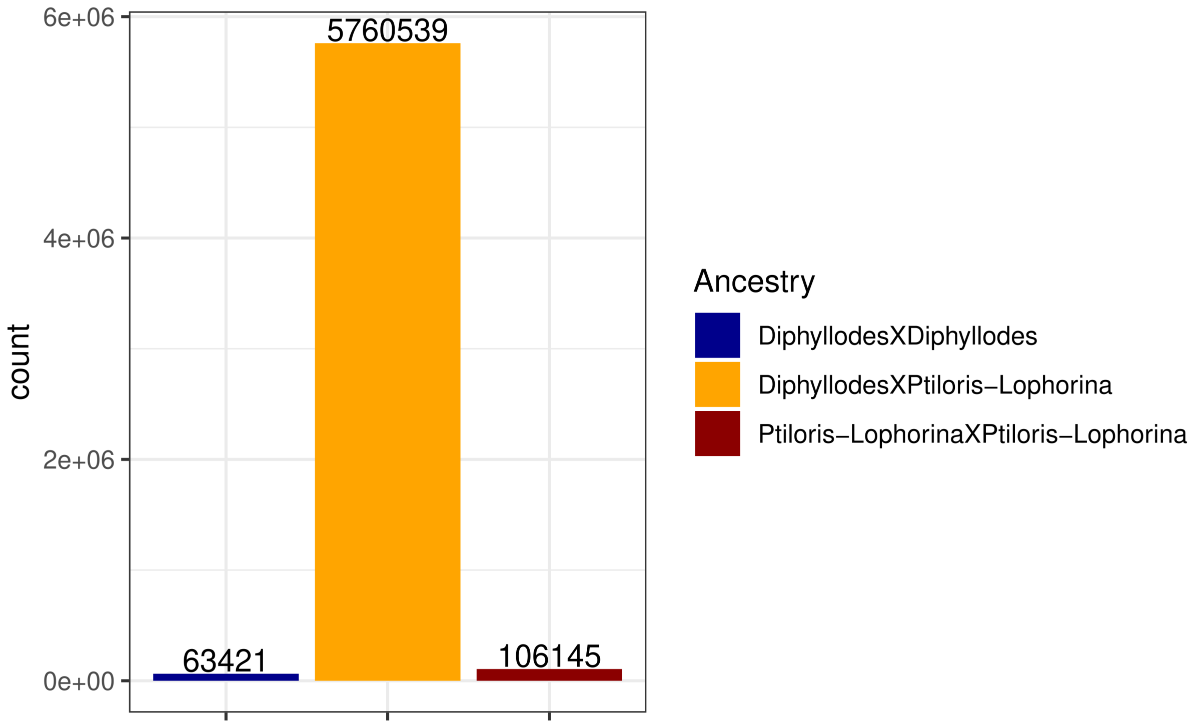

FigureS48. AIMs in bins of 100 positions along autosomes and Z chromosome in LopCic115 indicating its F1-hybrid status. Counts of homozygous and heterozygous AIMs in LopCic115 indicating its F1-hybrid status. Samples marked with index 9 in table S2 were used to produce this plot.

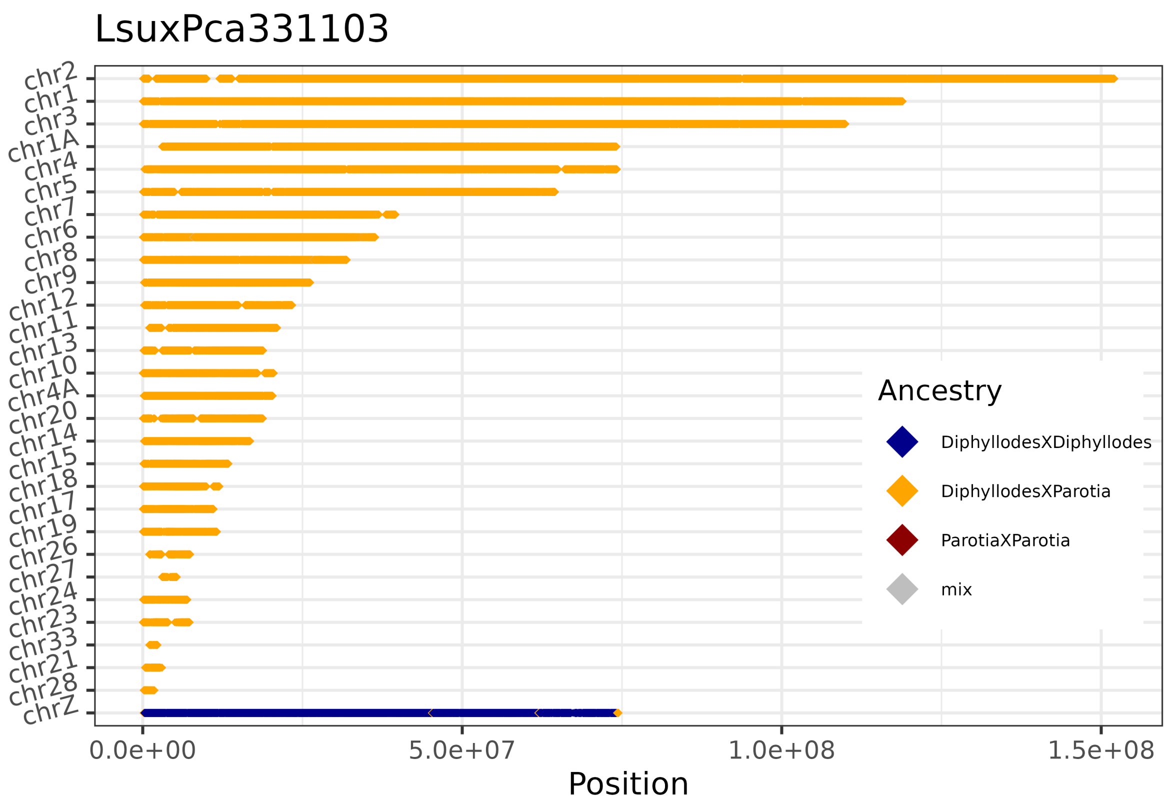

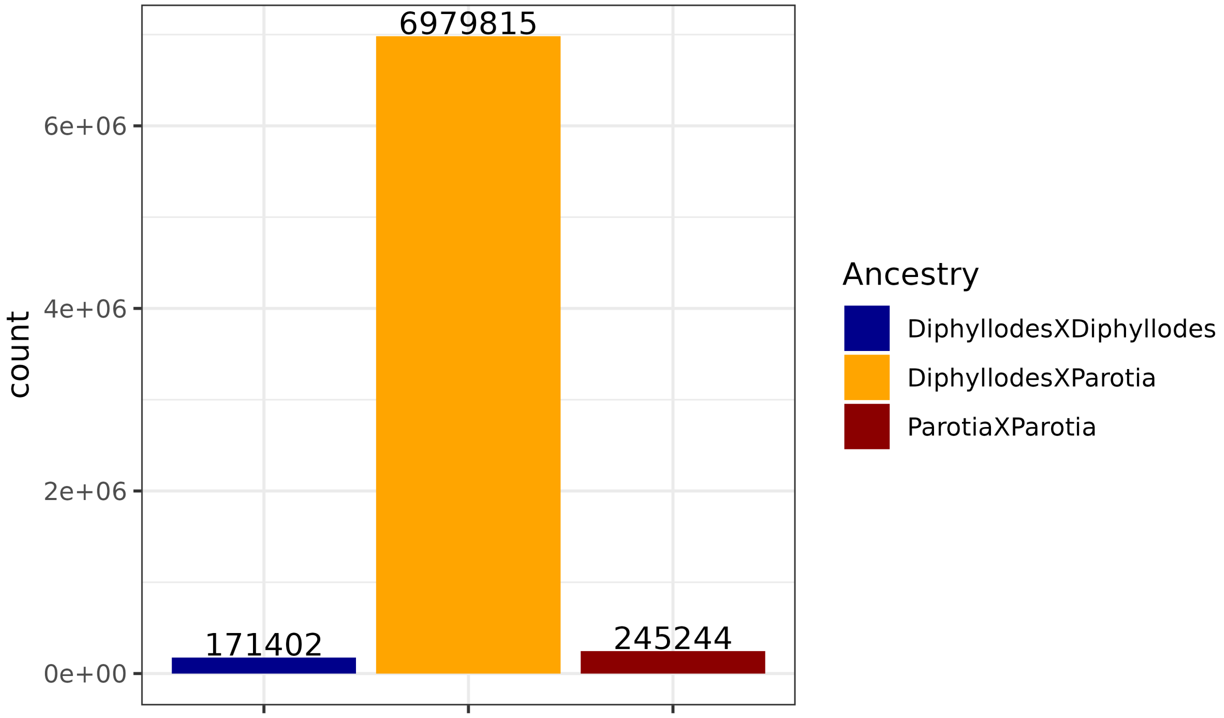

FigureS49. AIMs in bins of 100 positions along autosomes and Z chromosome in LsuxPca331103 indicating its F1-hybrid status. Counts of homozygous and heterozygous AIMs in LsuxPca331103 indicating its F1-hybrid status. Samples marked with index 11 in table S2 were used to produce this plot.

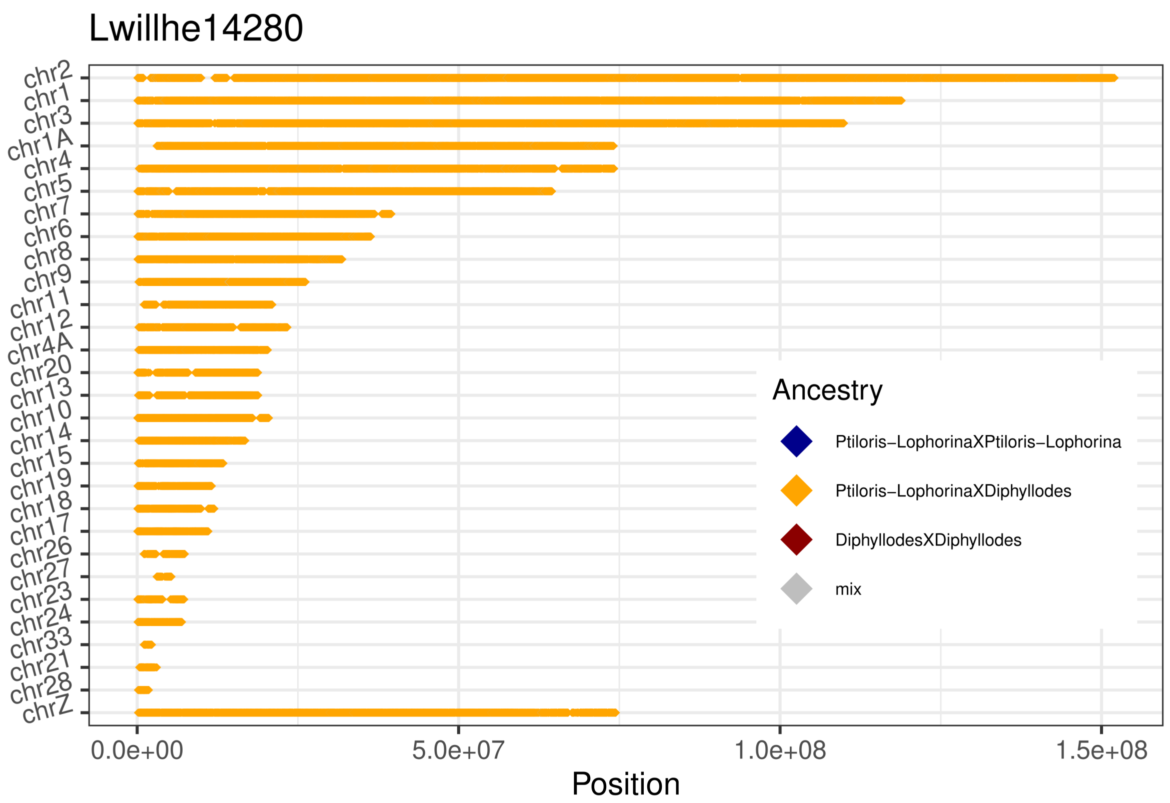

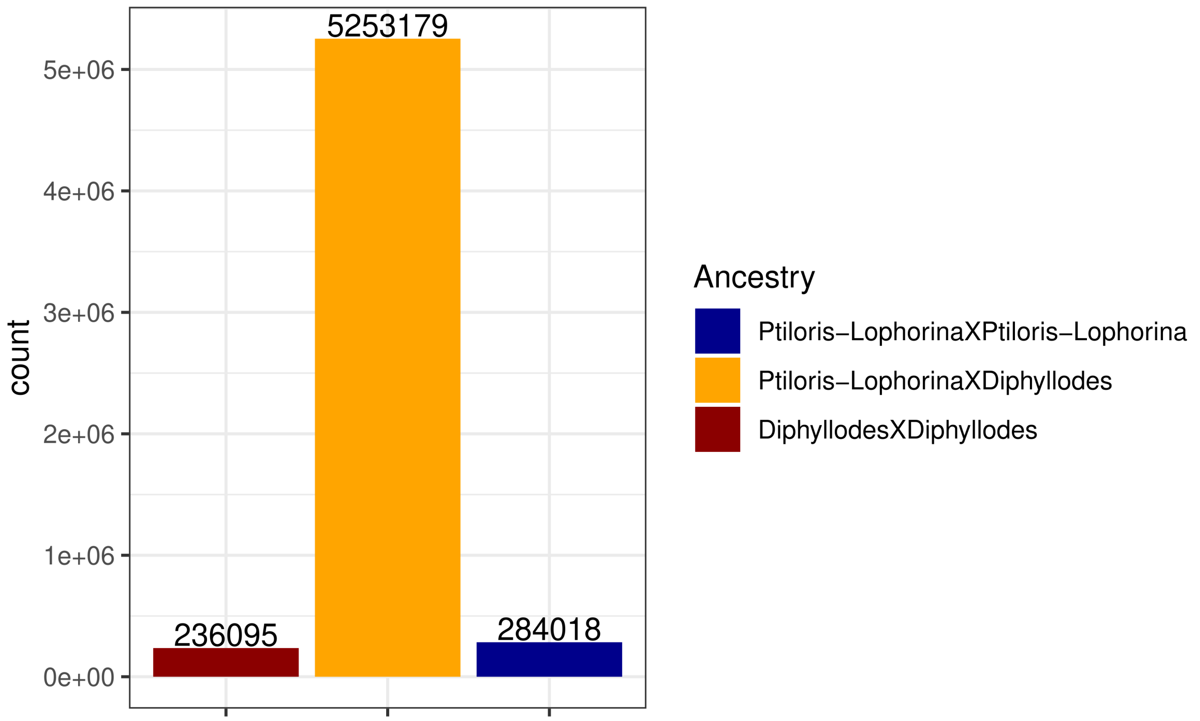

FigureS50. AIMs in bins of 100 positions along autosomes and Z chromosome in Lwillhe14280 indicating its F1-hybrid status. Counts of homozygous and heterozygous AIMs in Lwillhe14280 indicating its F1-hybrid status. Samples marked with index 9 in table S2 were used to produce this plot.

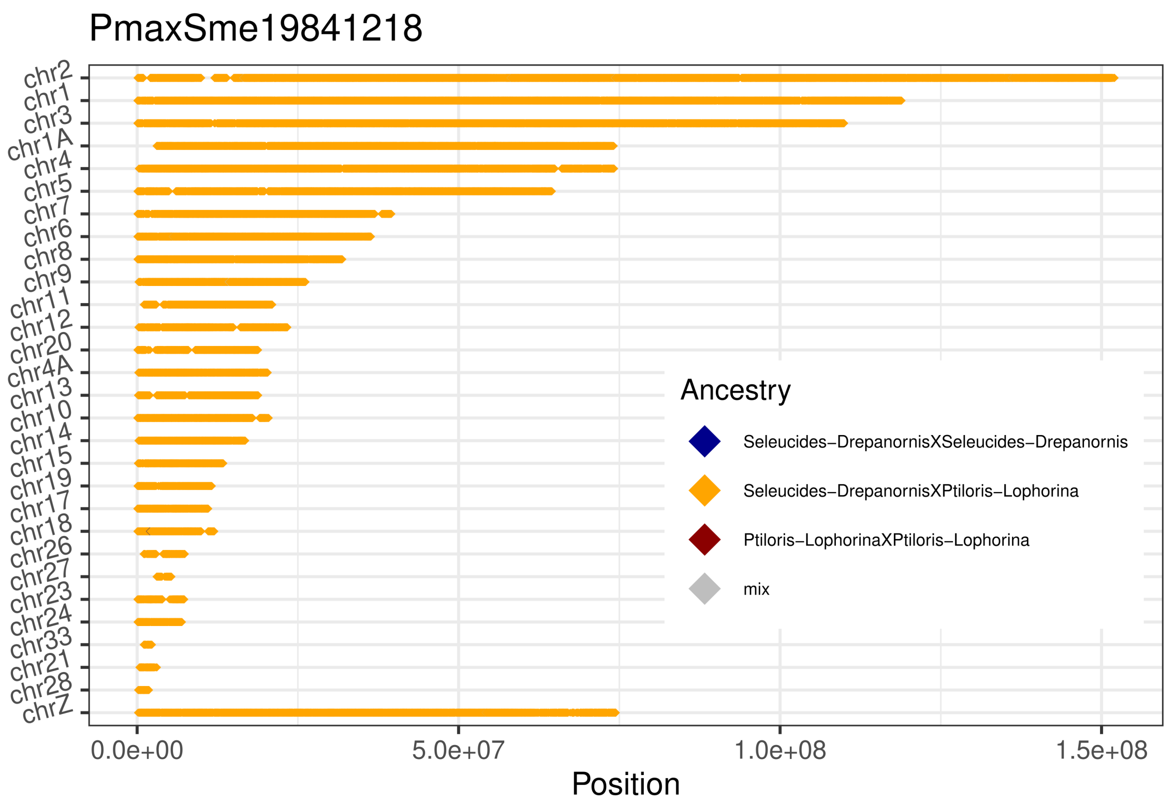

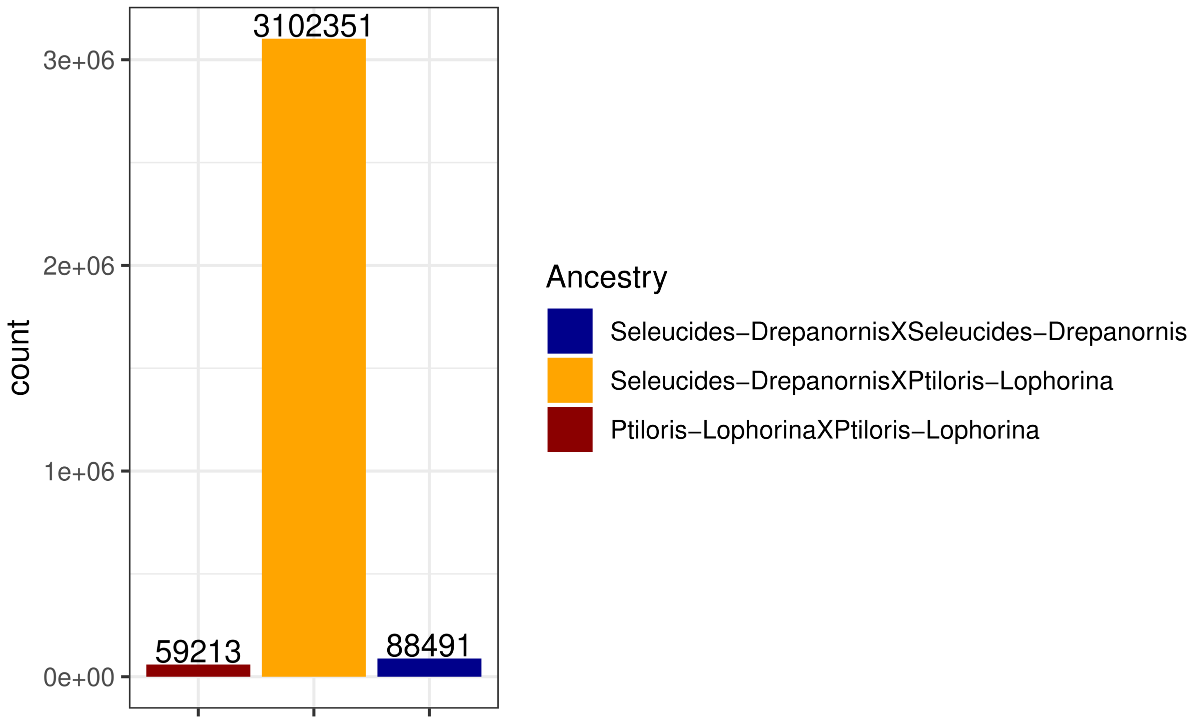

FigureS51. AIMs in bins of 100 positions along autosomes and Z chromosome in PmaxSme19841218 indicating its F1-hybrid status. Counts of homozygous and heterozygous AIMs in PmaxSme19841218 indicating its F1-hybrid status. Samples marked with index 19 in table S2 were used to produce this plot.

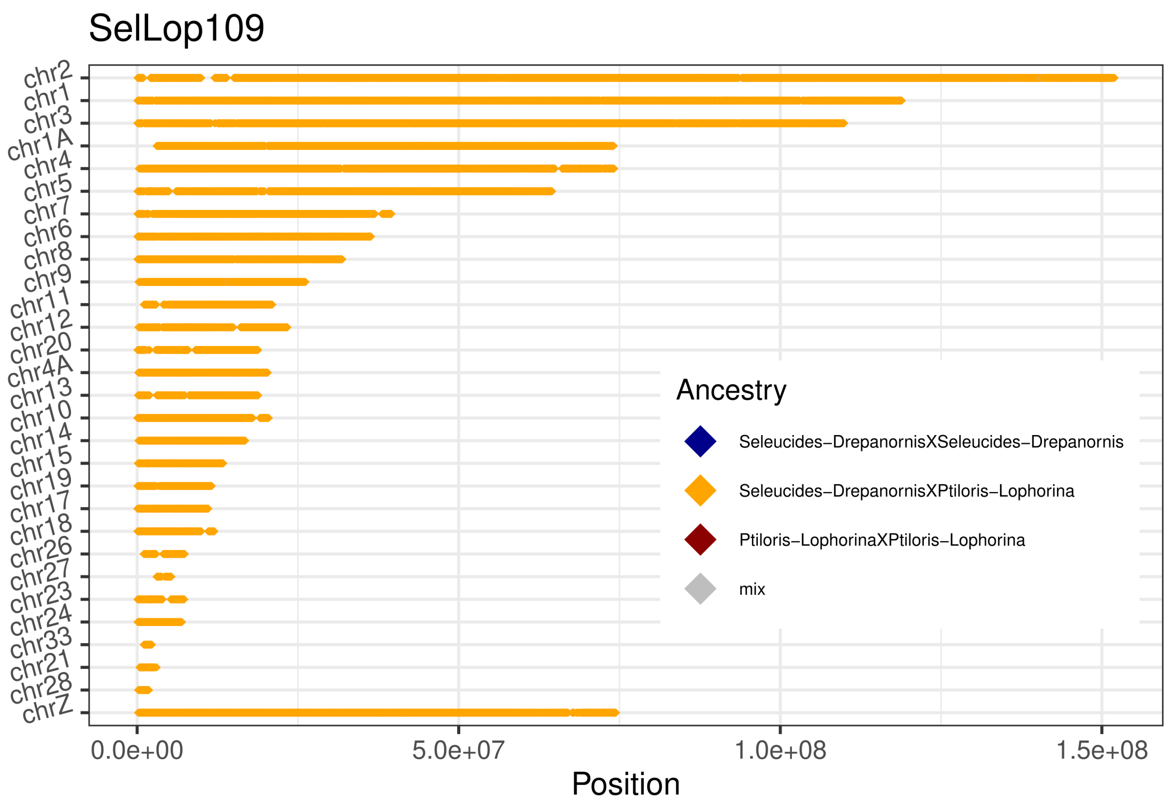

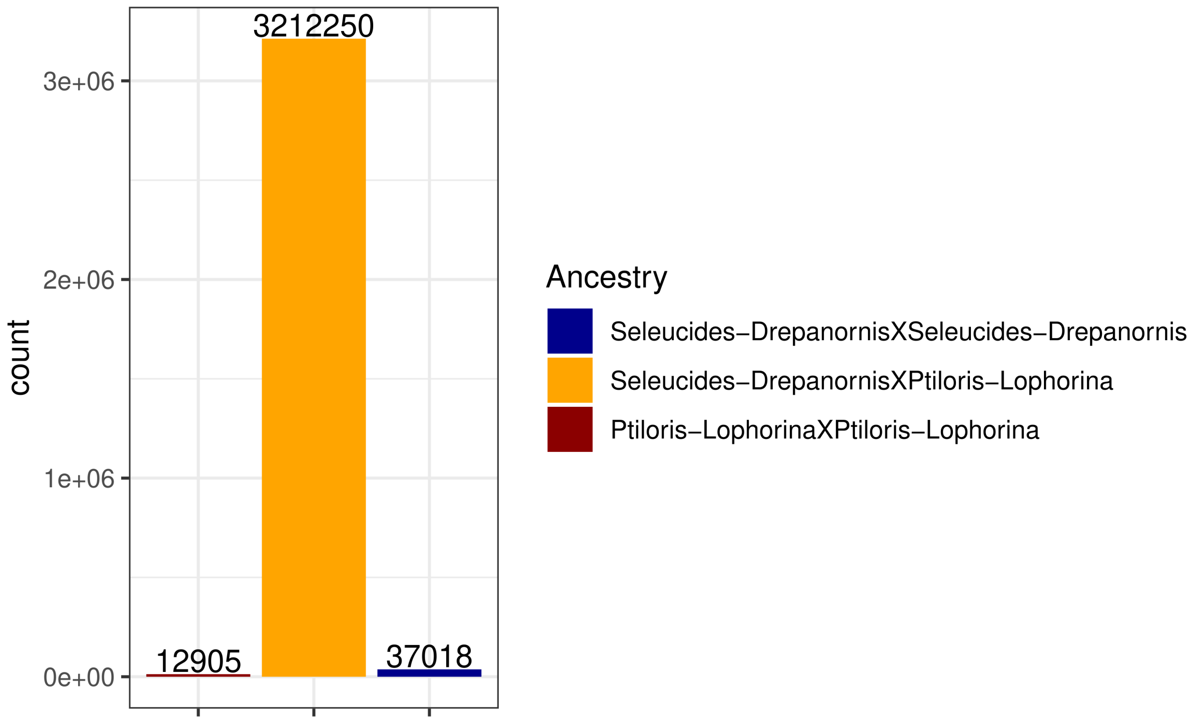

FigureS52. AIMs in bins of 100 positions along autosomes and Z chromosome in SelLop109 indicating its F1-hybrid status. Counts of homozygous and heterozygous AIMs in SelLop109 indicating its F1-hybrid status. Samples marked with index 19 in table S2 were used to produce this plot.

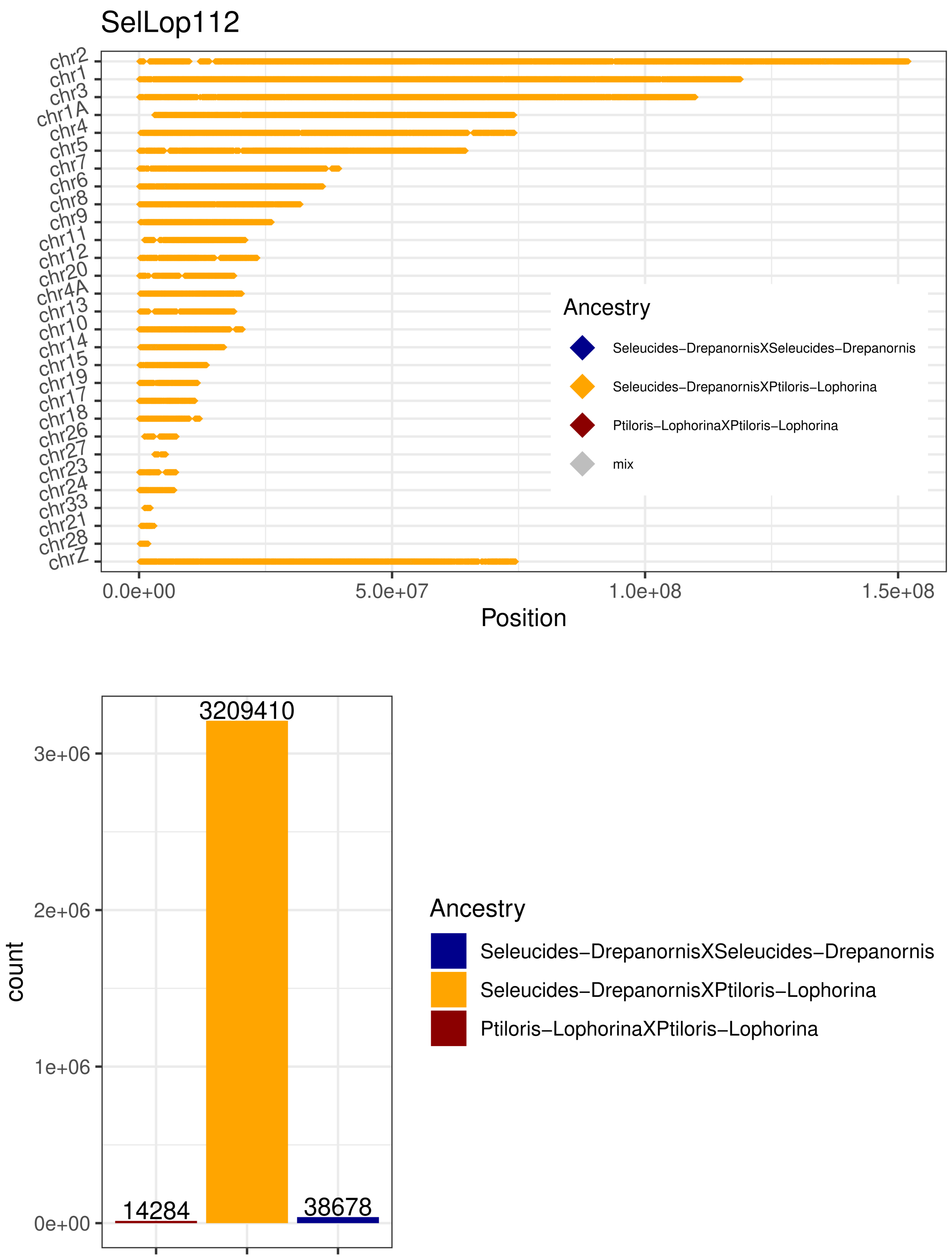

FigureS53. AIMs in bins of 100 positions along autosomes and Z chromosome in SelLop112 indicating its F1-hybrid status. Counts of homozygous and heterozygous AIMs in SelLop112 indicating its F1-hybrid status. Samples marked with index 19 in table S2 were used to produce this plot.

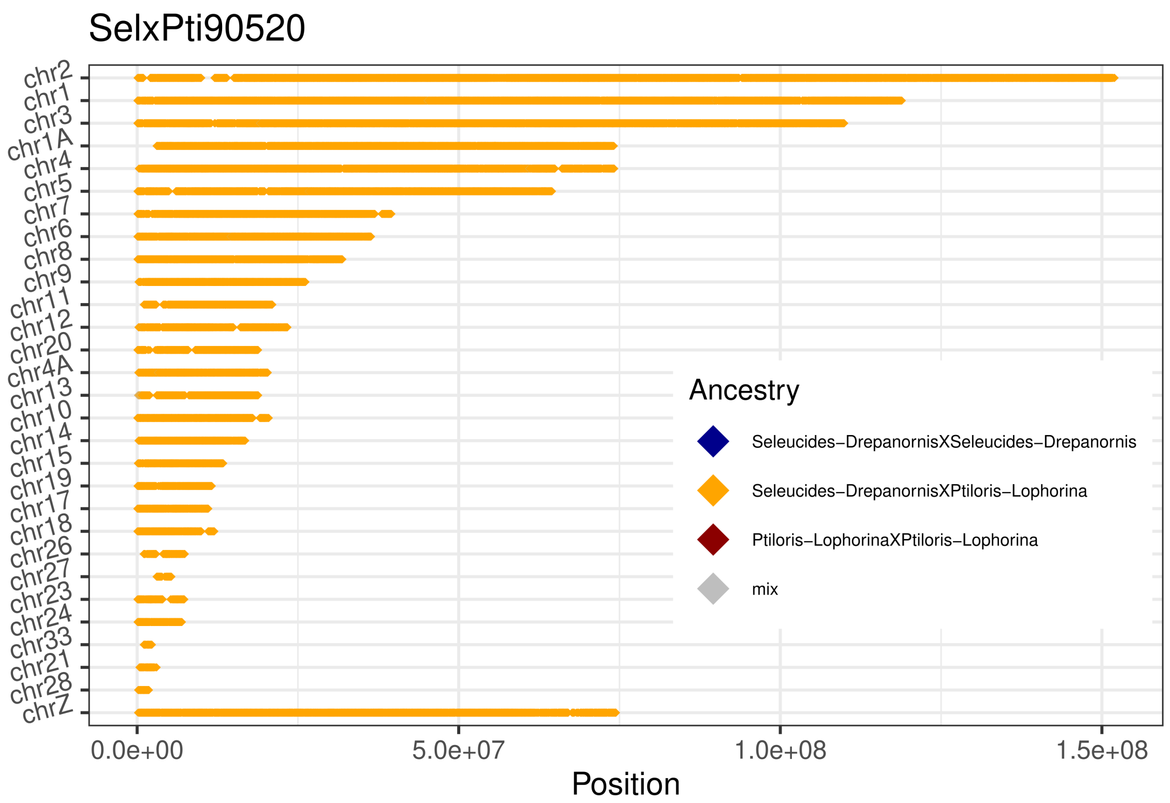

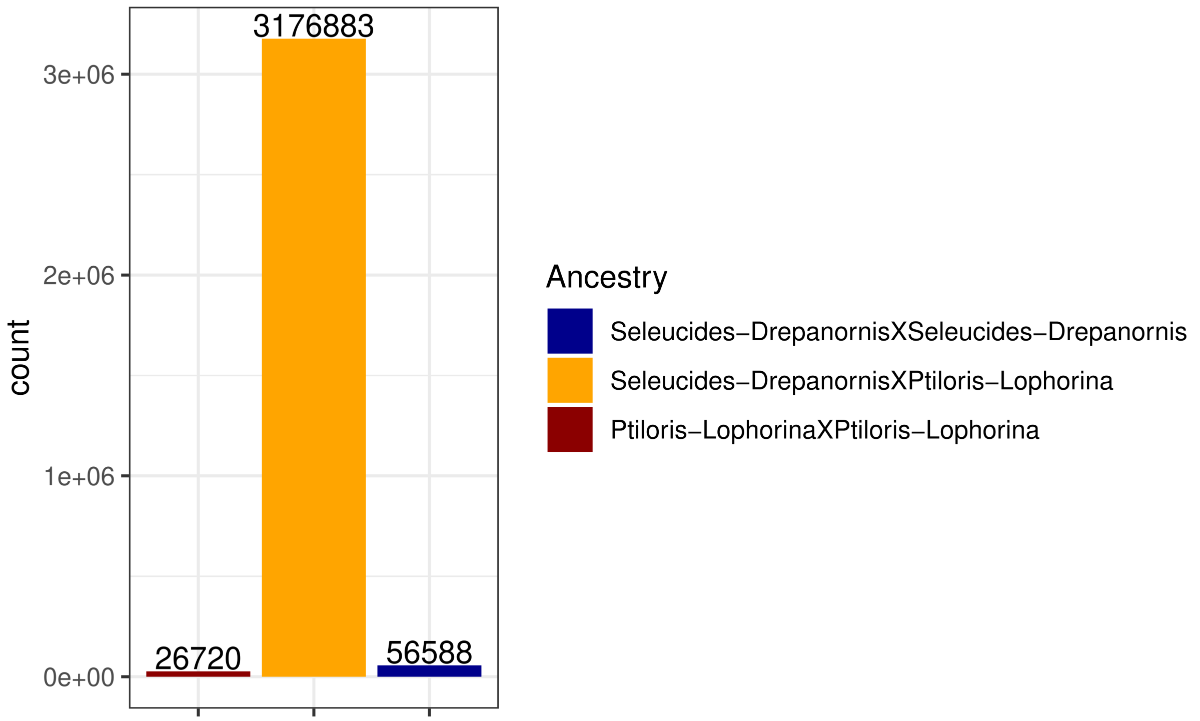

FigureS54. AIMs in bins of 100 positions along autosomes and Z chromosome in SelxPti90520 indicating its F1-hybrid status. Counts of homozygous and heterozygous AIMs in SelxPti90520 indicating its F1-hybrid status.Samples marked with index 19 in table S2 were used to produce this plot.

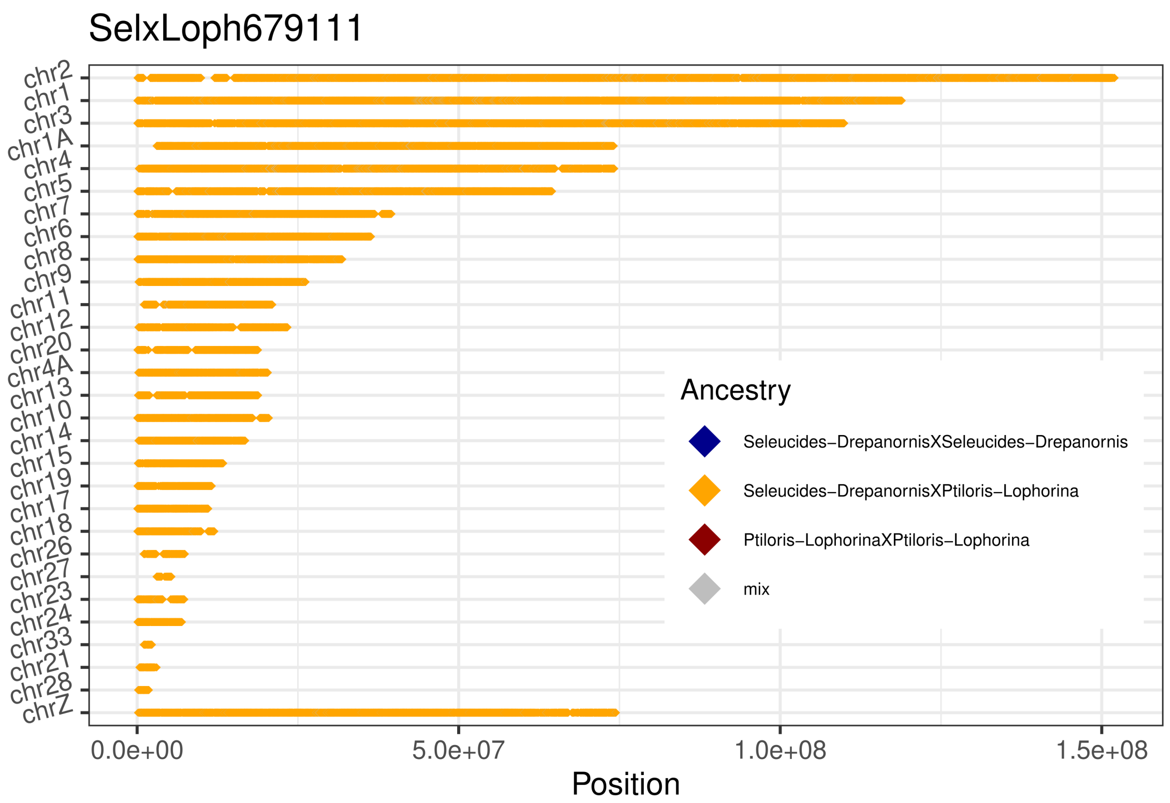

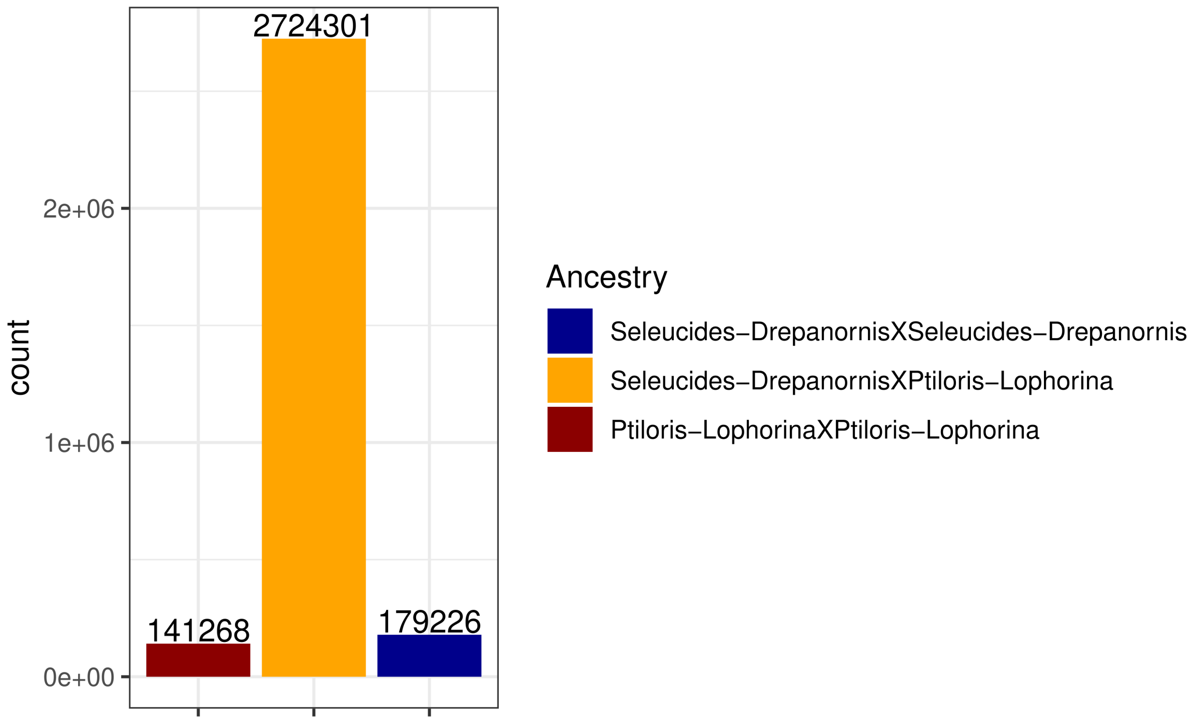

FigureS55. AIMs in bins of 100 positions along autosomes and Z chromosome in SelxLoph679111 indicating its F1-hybrid status. Counts of homozygous and heterozygous AIMs in SelxLoph679111 indicating its F1-hybrid status. Samples marked with index 19 in table S2 were used to produce this plot.

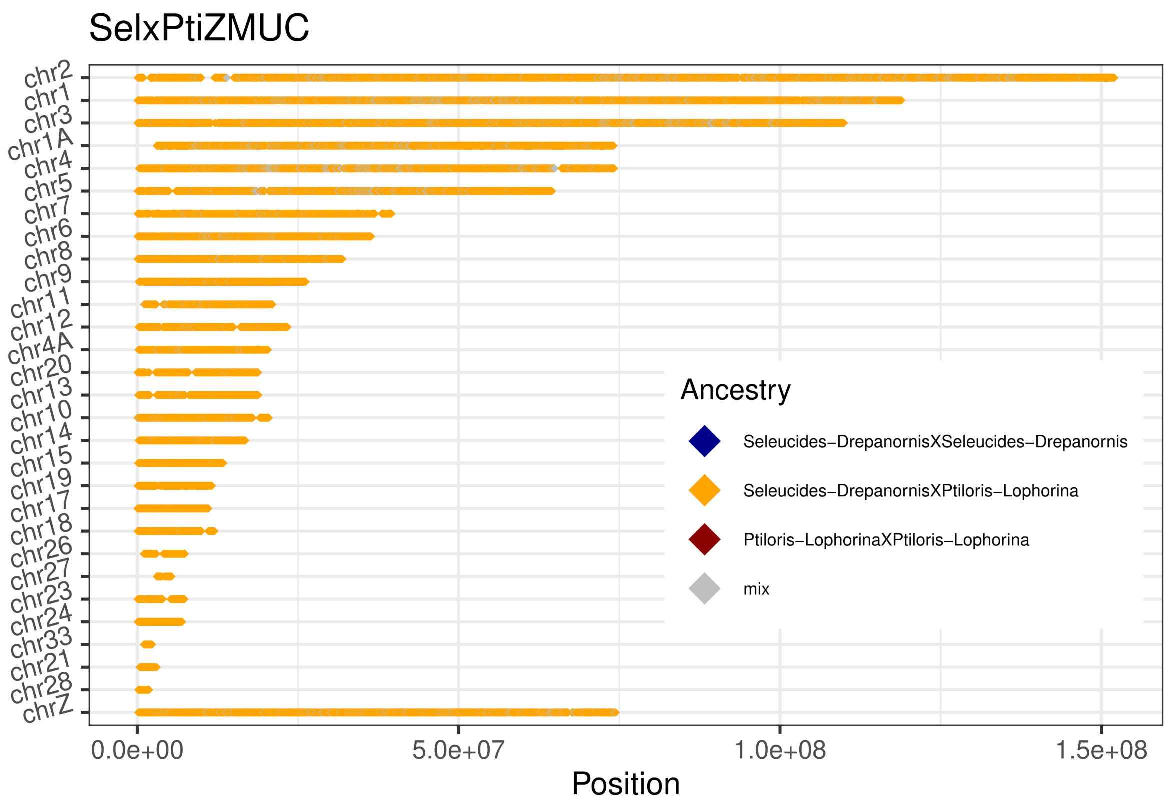

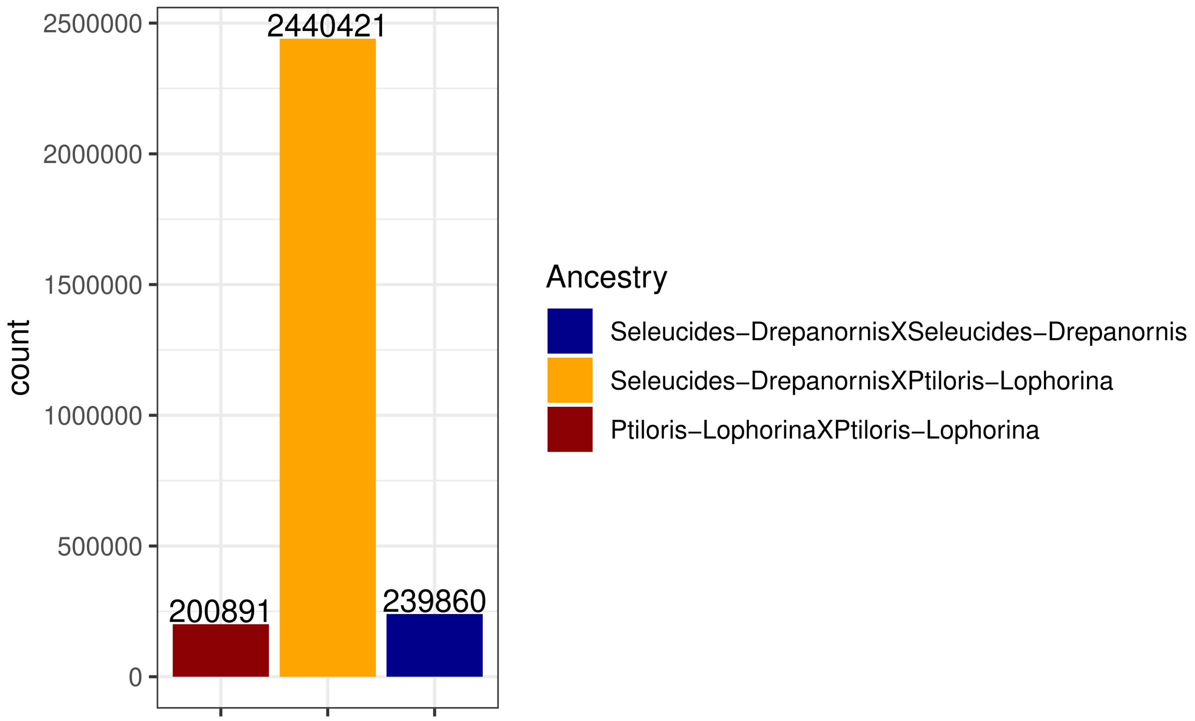

FigureS56. AIMs in bins of 100 positions along autosomes and Z chromosome in SelxPtiZMUC indicating its F1-hybrid status. Counts of homozygous and heterozygous AIMs in SelxPtiZMUC indicating its F1-hybrid status. Samples marked with index 19 in table S2 were used to produce this plot.

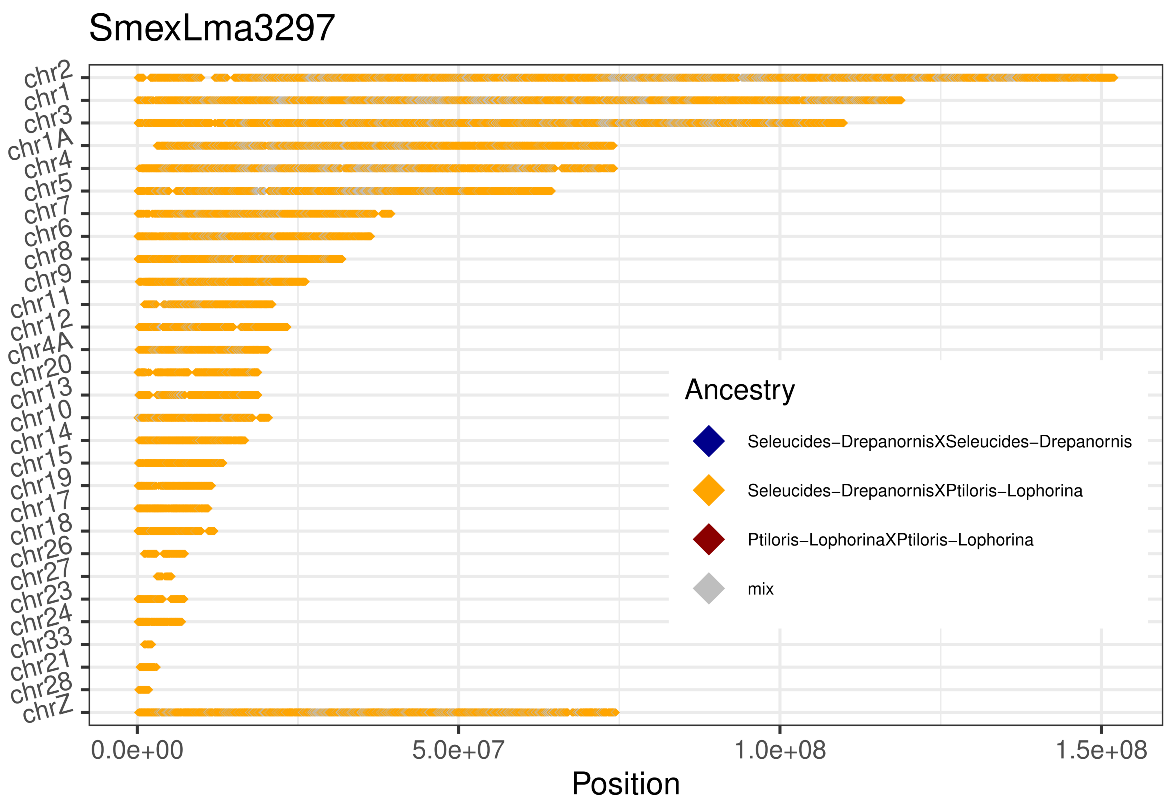

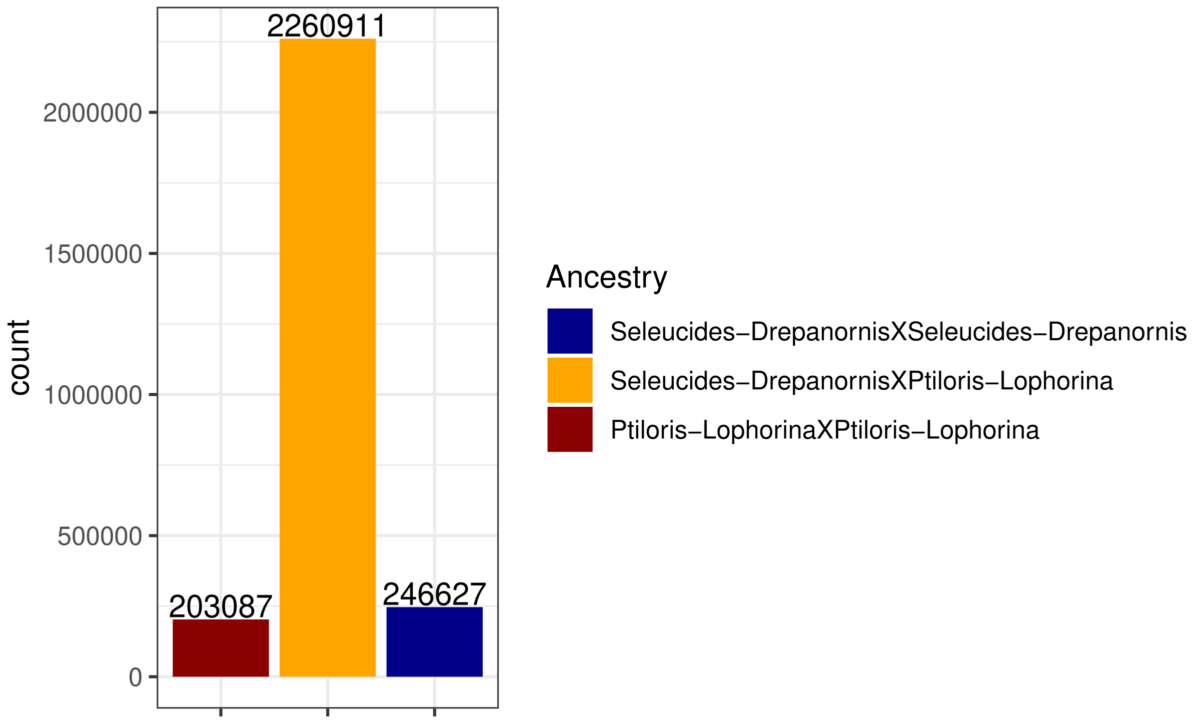

FigureS57. AIMs in bins of 100 positions along autosomes and Z chromosome in SmexLma3297 indicating its F1-hybrid status. Counts of homozygous and heterozygous AIMs in SmexLma3297 indicating its F1-hybrid status. Samples marked with index 19 in table S2 were used to produce this plot.

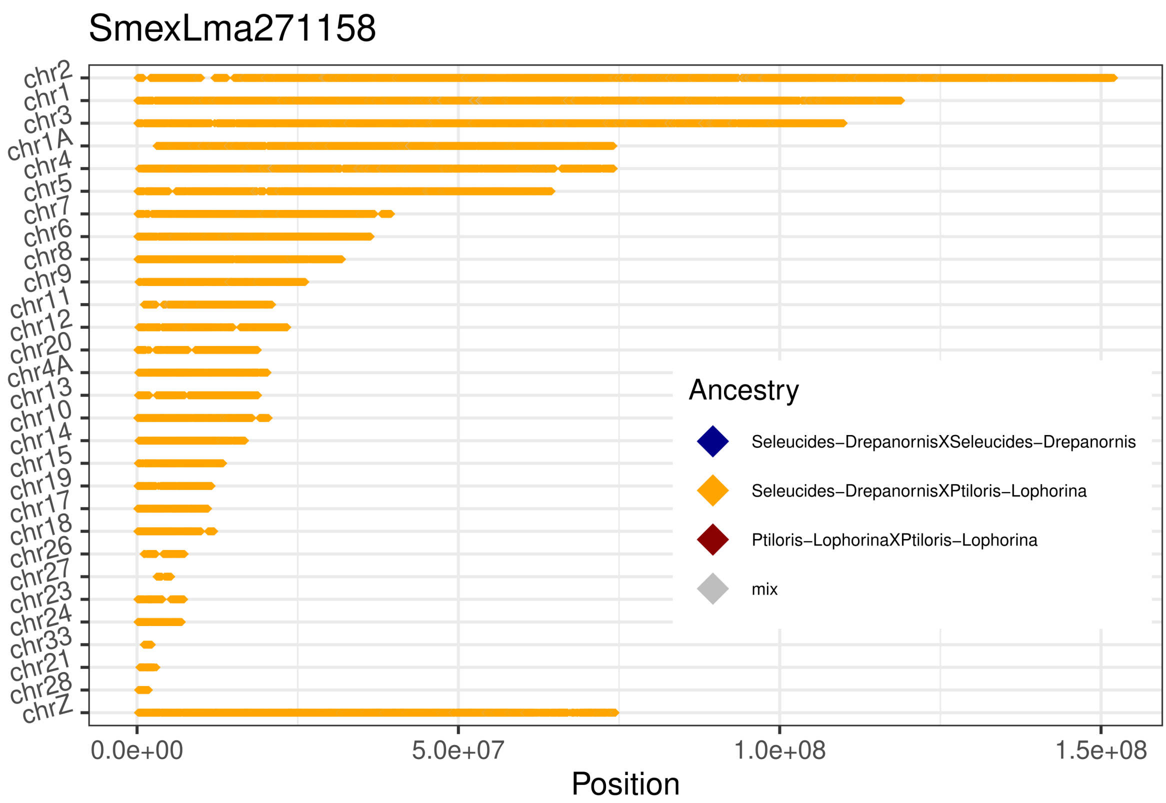

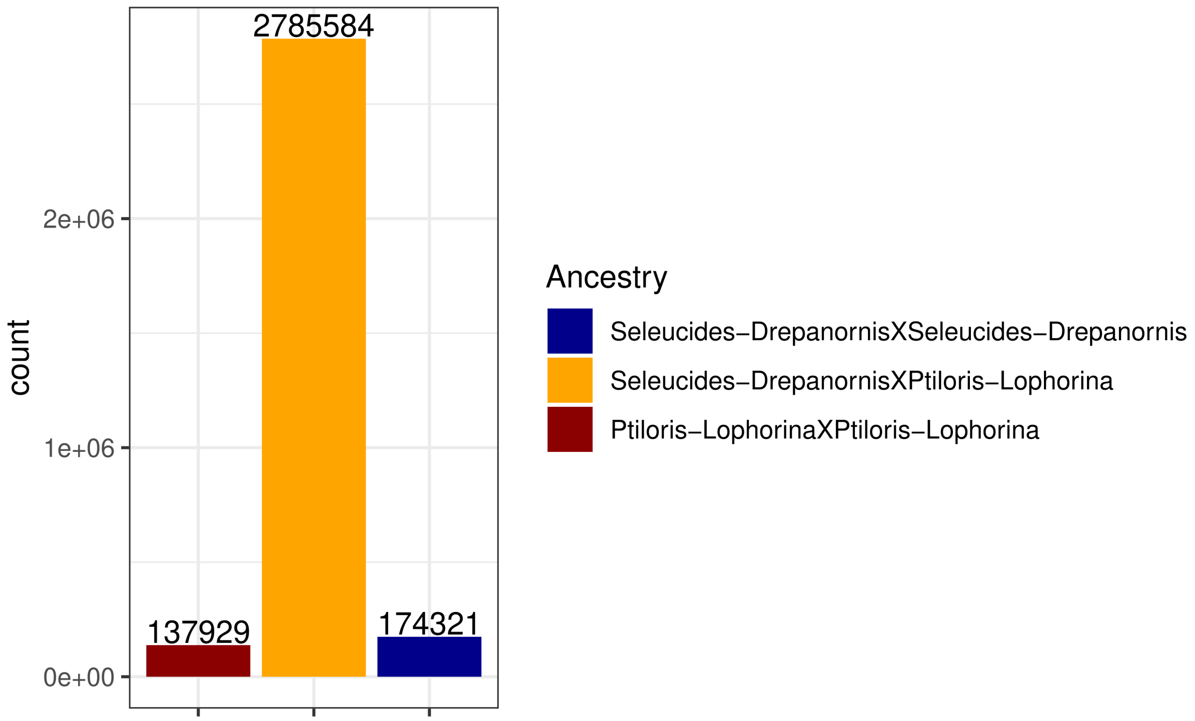

FigureS58. AIMs in bins of 100 positions along autosomes and Z chromosome in SmexLma271158 indicating its F1-hybrid status. Counts of homozygous and heterozygous AIMs in SmexLma271158 indicating its F1-hybrid status. Samples marked with index 19 in table S2 were used to produce this plot.

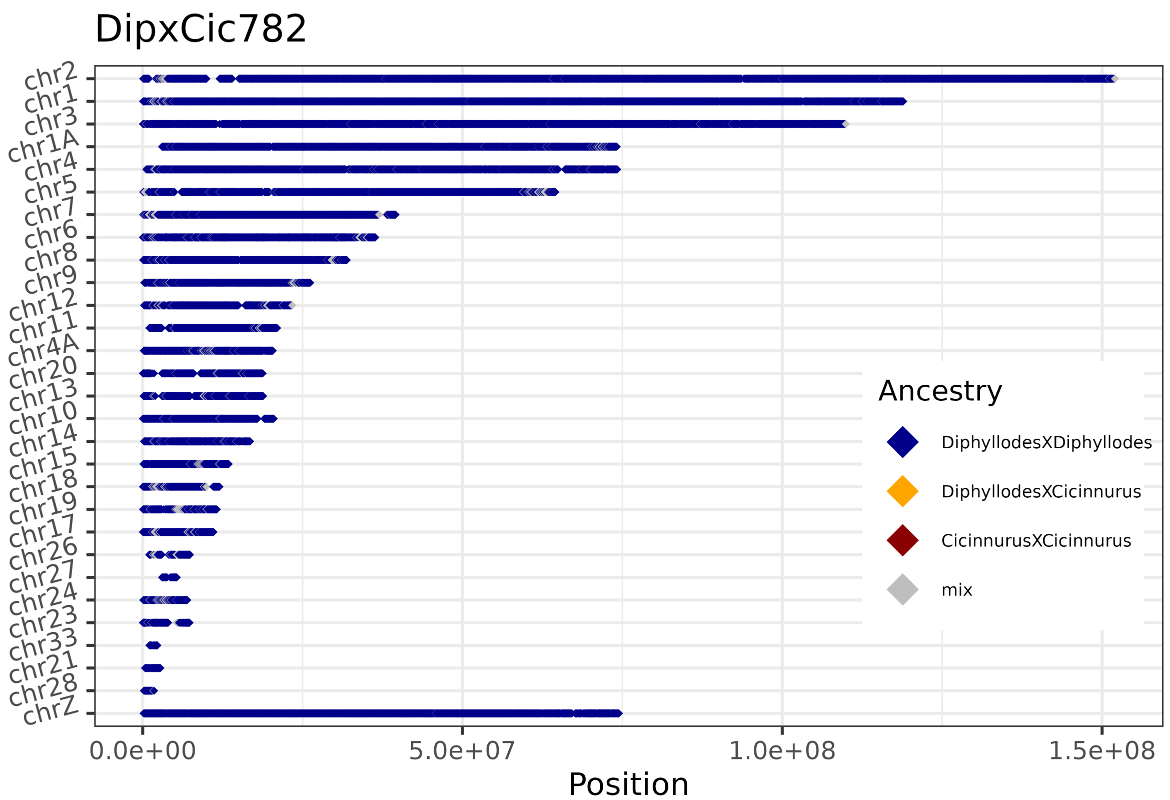

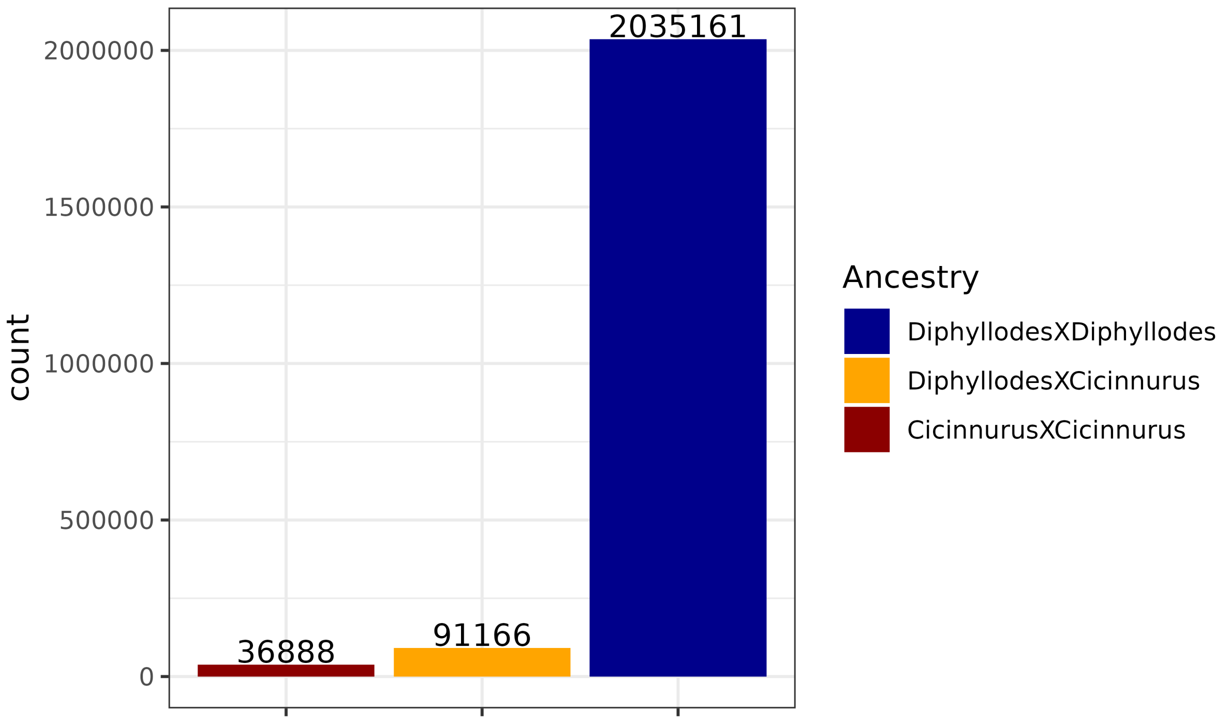

FigureS59. AIMs in bins of 100 positions along autosomes and Z chromosome in DipxCic782 indicating the sample to be a misidentified conspecific *Diphyllodes*. Counts of homozygous and heterozygous AIMs in DipxCic782 indicating the sample to be a misidentified *Diphyllodes*. Samples marked with index 3 in table S2 were used to produce this plot.

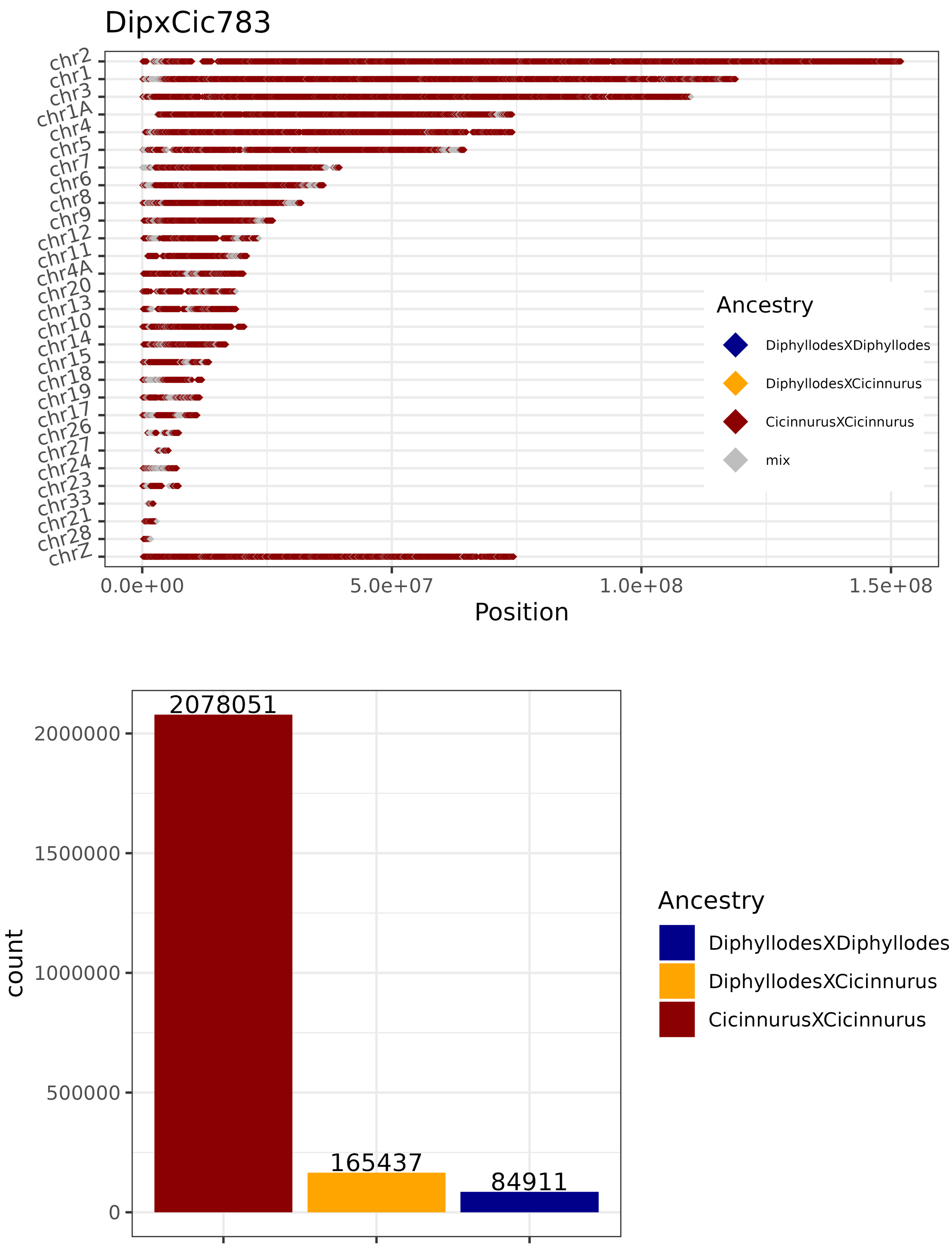

FigureS60. AIMs in bins of 100 positions along autosomes and Z chromosome in DipxCic783 indicating the sample to be a misidentified conspecific *Cicinnurus*. Counts of homozygous and heterozygous AIMs in DipxCic783 indicating the sample to be a misidentified *Cicinnurus*. Samples marked with index 3 in table S2 were used to produce this plot.

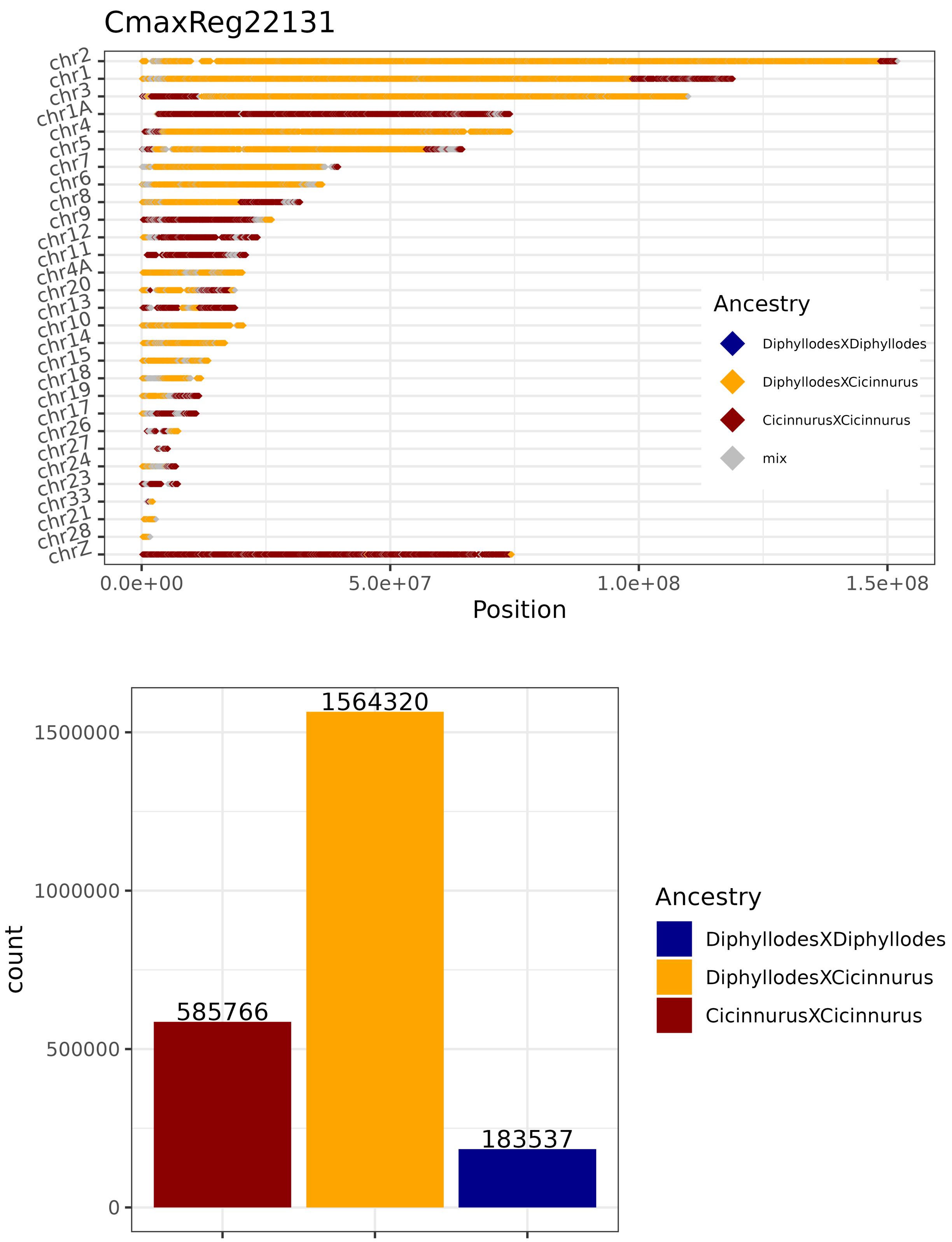

FigureS61. AIMs in bins of 100 positions along autosomes and Z chromosome in CmaxReg22131 indicating contemporary introgression. Counts of homozygous and heterozygous AIMs in CmaxReg22131 indicating it not beeing a F1-hybrid. Samples marked with index 3 in table S2 were used to produce this plot.

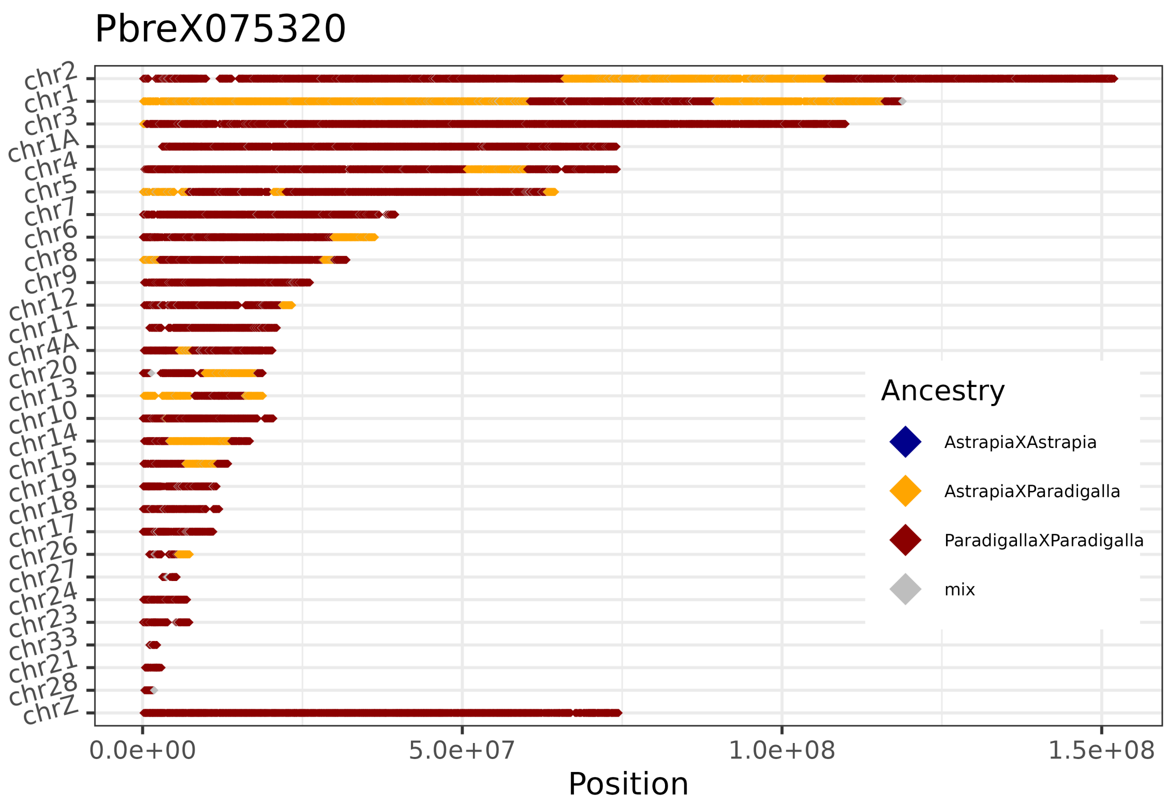

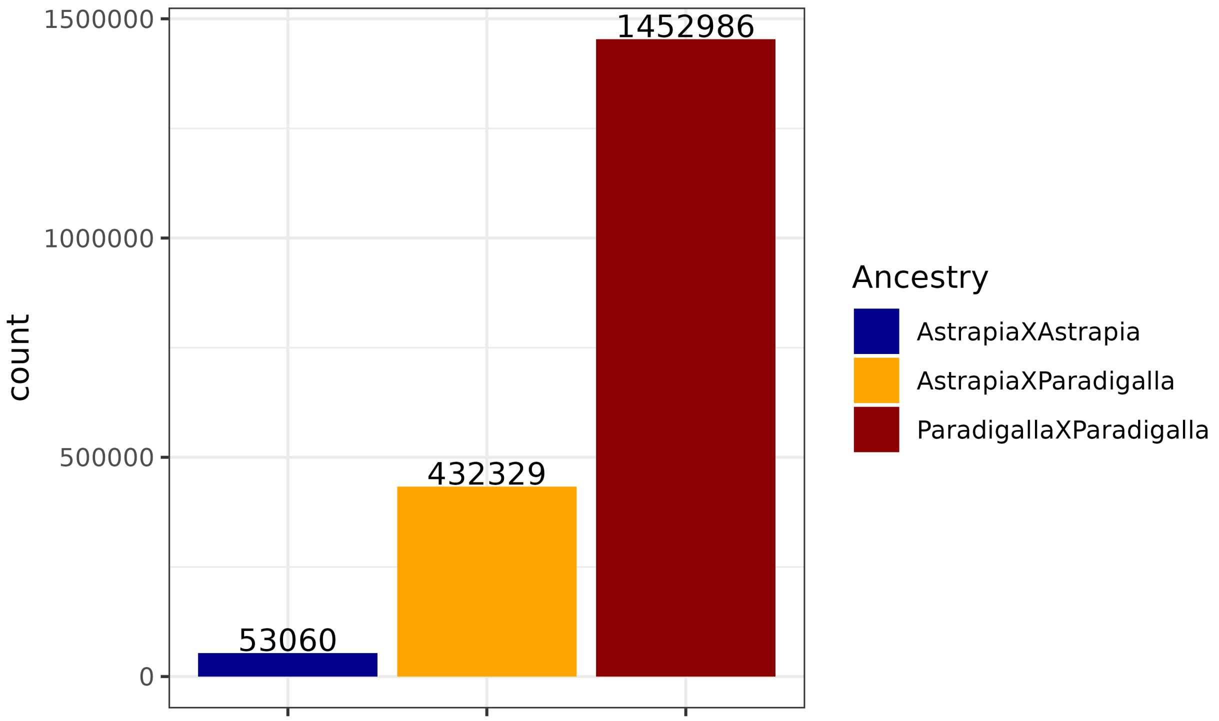

FigureS62. AIMs in bins of 100 positions along autosomes and Z chromosome in PbreX075320 indicating contemporary introgression. Counts of homozygous and heterozygous AIMs in PbreX075320 indicating it not beeing a F1-hybrid. Samples marked with index 1 in table S2 were used to produce this plot.

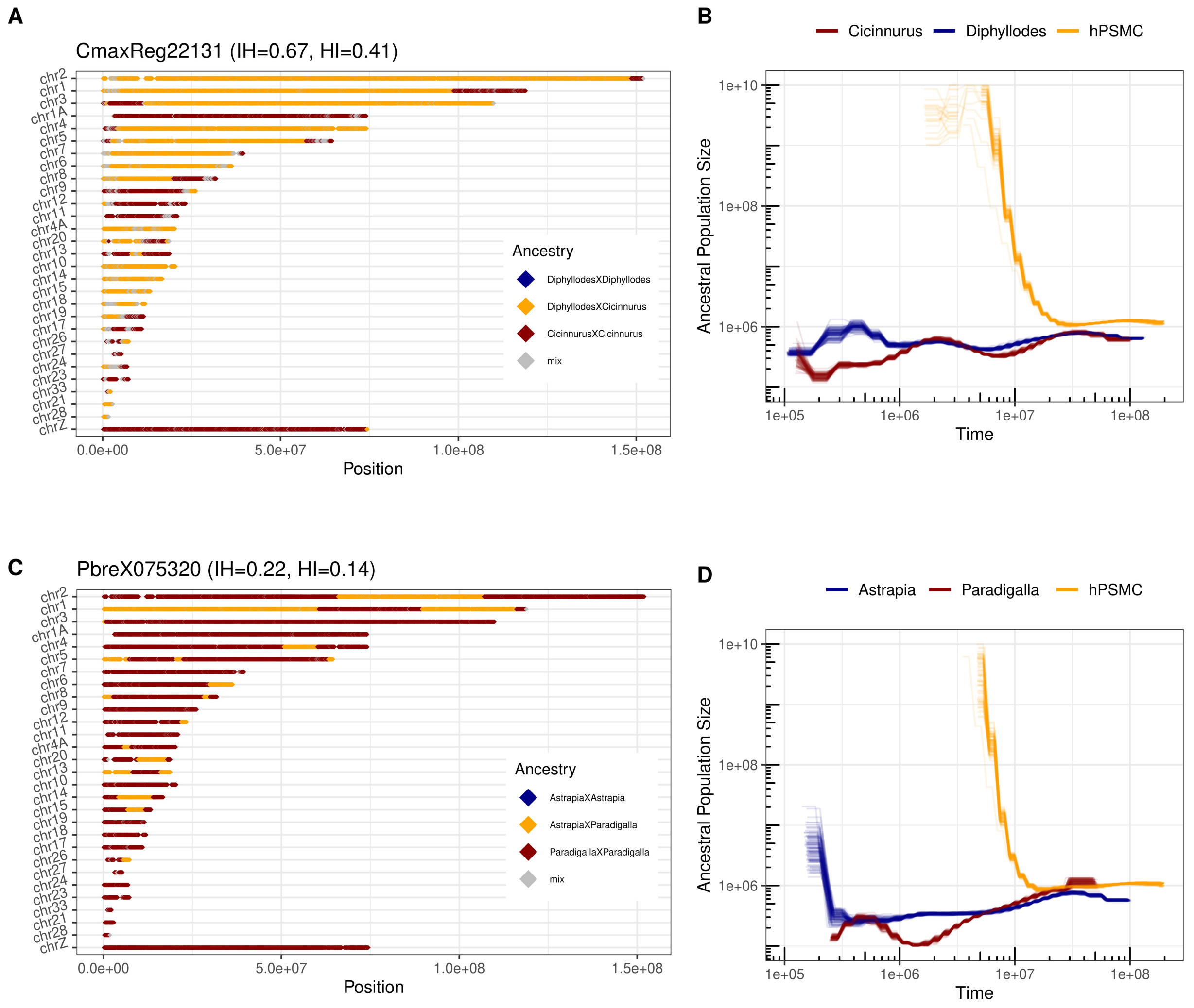

FigureS63. A) AIMs in bins of 100 positions along autosomes and the Z chromosome in CmaxReg22131 indicating it has experienced recent introgression. Interspecific heterozygosity (IH) and Hybrid index (HI). Samples marked with index 3 in table S2 were used to produce this plot. B) hPSMC curve of 100 bootstraps for an artificial F1-hybrid between Diphyllodes and Cicinnurus indicating that the initial end of gene flow occurred more than 10 Mya (Millions of years ago). PSMC curves of demography of Diphyllodes and Cicinnurus included as well, generated for samples indexed with 21 in table S2. Plots were generated using a mutation rate of 1.4e-9 and a generation time of 8 years. C) AIMs in bins of 100 positions along autosomes and the Z chromosome in PbreX075320 indicating it has experienced recent introgression. Interspecific heterozygosity (IH) and Hybrid index (HI). Samples marked with index 3 in table S2 were used to produce this plot. D) hPSMC curve of 100 bootstraps for an artificial F1-hybrid indicating that the initial end of gene flow between Astrapia and Paradigalla occurred around 10 Mya. PSMC curves of demography of Astrapia and Paradigalla included as well, generated for samples indexed with 22 in table S2. Plots were generated using a mutation rate of 1.4e-9 and a generation time of 8 years.

Reinvestigation of hybrids with different parental genera combinations

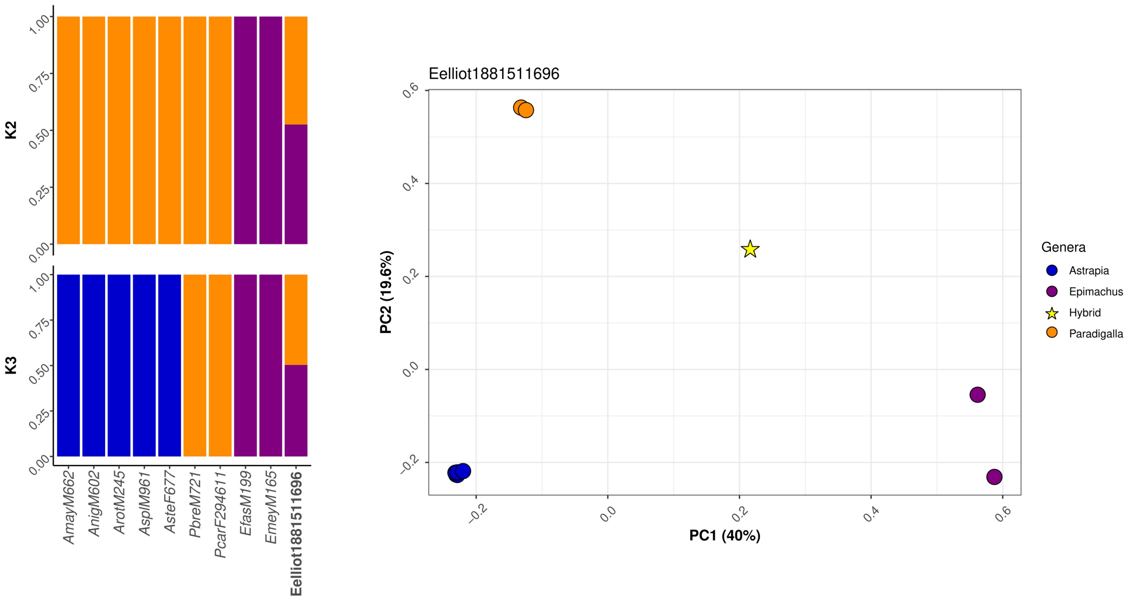

FigureS64. PCA and admixture analysis K=2..3 parental genera implemented through PCAngsd and NGSAdmix. Hybrid is marked with a yellow star in the PCA plot. Eelliot1881511696 was assessed to be a hybrid between *Astrapia* and *Epimachus* but these plots indicate the parental genera to be *Epimachus* and *Paradigalla*. Samples marked with index 17 in table S2 were used in combination with the hybrid Eelliot1881511696 to produce this plot.

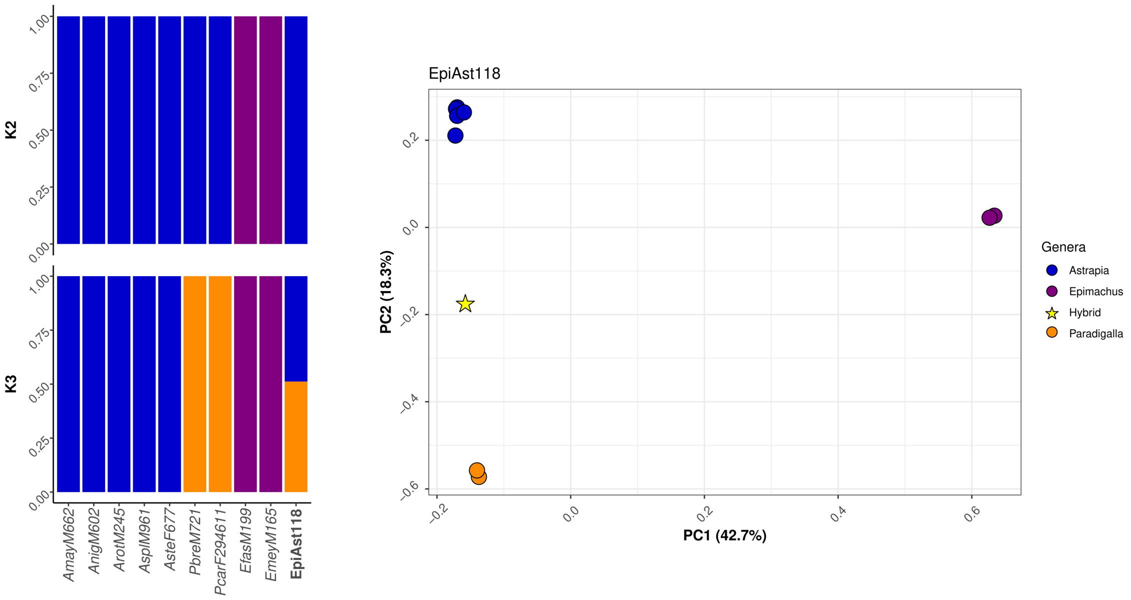

FigureS65 PCA and admixture analysis K=2..3 parental genera implemented through PCAngsd and NGSAdmix. Hybrid is marked with a yellow star in the PCA plot. EpiAst118 was assessed to be a hybrid between *Paradigalla* and *Epimachus* but these plots indicate the parental genera to be *Astrapia* and *Paradigalla*. Samples marked with index 17 in table S2 were used in combination with the hybrid EpiAst118 to produce this plot.

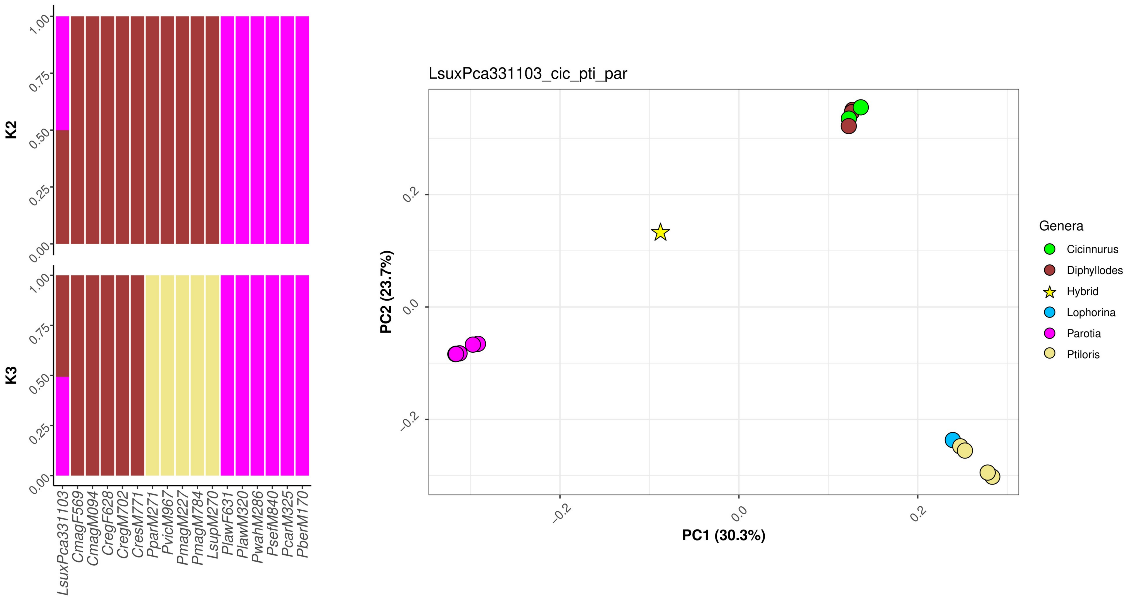

FigureS66. PCA and admixture analysis K=2..3 parental genera implemented through PCAngsd and NGSAdmix. Hybrid is marked with a yellow star in the PCA plot. LsuxPca331103 was assessed to be a hybrid between *Parotia* and *Lophorina* but these plots indicate the parental genera to be *Parotia* and *Diphyllodes/Cicinnurs*. Samples marked with index 18 in table S2 were used in combination with the hybrid LsuxPca331103 to produce this plot.

Supplementary tables

Table S1. Summary of all hybrids investigated and the result of our assessment. Table containing: Sample ID, Depth of coverage, Voucher name, Collection year*, Original description, Vernacular name, Morphological assessment, Source, Sex, mtDNA identity, PCAngsd identity, NGSadmix identity, Morphological assessment=genetic, and Hybrid level. *A large proportion of bird-of-paradise hybrids i Natural History collections are trade skins. This makes of impossible to locate the exact collection year or the localities where they have been collected. However, the vast majority of these trade skins are with certainty collected during the 19th century.

Table S2. Reference genomes and per analyses subsets. Summary of all samples used as reference material from Blom (et al. in review) obtained from the European Nucleotide Archive (PRJEB64275). Table containing: Sample ID, Genus, Species, Voucher, tissue type, Sex, and Subset indices*. *The column “Subset indices” contains indices indicating which samples were used as the reference material to run the analyses. In the figure texts the group index is given so it that it is clear what samples have been used to produce each figure. Samples used in the mitochondrial phylogeny are noted with the index “mt”, subset indices between 1-21 were used for the PCA, admixture, AIMs analyses as well as the triangle plot, subsets with indices 22 and 23 were used for the hPSMC analyses.

Museum code key:

AM = Australian Museum, Sydney
AMNH = American Museum of Natural History, New York
ANWC = Australian National Wildlife Collection, Canberra
BMNH (NHM) = Natural History Museum, London
KU = Kansas university, Kansas
MNHN = Muséum national d'Histoire naturelle, Paris
NRM = Swedish Museum of Natural History, Stockholm
MZB = Museum für Naturkunde, Berlin.
RMNH = Naturalis Biodiversity Center, Leiden
SNSD = Senckenberg Naturhistorische Sammlungen Dresden, Dresden
YPM = Yale Peabody Museum
ZMA = Naturalis Biodiversity Center, Leiden
ZMUC = Zoological Museum, University of Copenhagen
